# Plasmodesmal closure elicits stress responses

**DOI:** 10.1038/s44319-026-00789-2

**Published:** 2026-05-02

**Authors:** Estee E Tee, Andrew Breakspear, Diana Papp, Hannah R Thomas, Catherine Walker, Annalisa Bellandi, Christine Faulkner

**Affiliations:** 1 Cell and Developmental Biology, John Innes Centre, Norwich Research Park, Norwich, NR4 7UH UK; 2 Present Address: Department of Metabolism, Digestion and Reproduction, Imperial College London, London, W12 0NN UK; 3 Present Address: Department of Horticulture, Zijingang Campus, Zhejiang University, Hangzhou, China; 4 Present Address: Laboratory of Biochemistry, Wageningen University, 6708WE Wageningen, Netherlands

**Keywords:** Microbiology, Virology & Host Pathogen Interaction, Plant Biology, Signal Transduction

## Abstract

Plant cells are connected to their neighbors via plasmodesmata facilitating the exchange of nutrients and signaling molecules. During immune responses, plasmodesmata close, but how this contributes towards a full immune response is unknown. To investigate this, we develop two transgenic lines which allow to induce plasmodesmal closure independently of immune elicitors, using the over-active CALLOSE SYNTHASE3 allele *icals3m* and the C-terminus of PDLP1 to drive callose deposition at plasmodesmata. Induction of plasmodesmal closure increases the expression of stress responsive genes, salicylic acid accumulation and resistance to *Pseudomonas syringae* DC3000. More homogeneous plasmodesmal closure using *icals3m* also leads to the accumulation of starch and sugars, decreases leaf growth, as well as hypersusceptibility to *Botrytis cinerea*. Based on the profile of responses, we conclude that plasmodesmal closure activates stress signaling, raising questions about the signals mediating this response and whether these responses occur in all circumstances when plasmodesmata close.

## Synopsis

Induction of plasmodesmal closure independent of a physiological elicitor is sufficient to induce stress signaling.

- Induced plasmodesmal closure activates the expression of stress responsive genes, salicylic acid accumulation, and enhances resistance to *Pseudomonas syringae* DC3000.
- Induced plasmodesmal closure leads to accumulation of starch and sugars, decreased leaf growth and hypersusceptibility to *Botrytis cinerea*.
- The signals that activate stress responses downstream of plasmodesmal closure remain to be identified.

## Abstract (web summary)

Induction of plasmodesmal closure independent of a physiological elicitor is sufficient to induce stress signaling.

## Graphical abstract

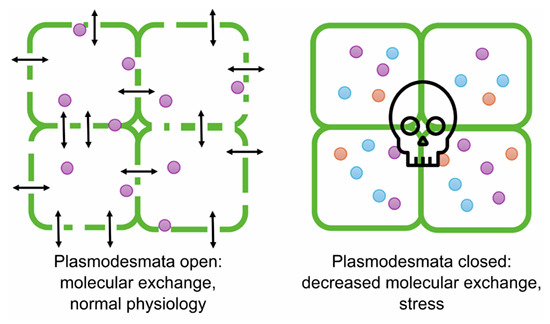

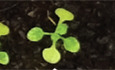

## Introduction

Plants are multicellular organisms, with almost every plant cell cytoplasmically connected to its neighbors. While most plant cells are equipped with machinery to respond autonomously to a range of stress signals, optimal responses involve the regulation of cytoplasmic connectivity between cells. This is particularly evident in immune signaling; most cells produce the receptors that perceive microbial threats and can activate appropriate responses, but the degree of connectivity to neighboring cells is a critical part of the full immune response (Lee et al, 2011; Faulkner et al, 2013). Indeed, if plants cannot regulate the cytoplasmic connectivity between cells, they are more susceptible to infection by a range of pathogenic microbes. Despite this observation, we don’t yet understand how cell-to-cell connectivity contributes to an overall immune response.

Cytoplasmic connectivity between plant cells is established via membrane-lined bridges called plasmodesmata. Plasmodesmata are dynamic structures and the oscillation in their aperture, i.e., closing and opening, allows control over the flux of soluble molecules between cells. We broadly assume that many molecules that move through plasmodesmata, including hormones, RNAs, and proteins, are information carriers and that aperture changes thus impact cell-to-cell communication. Indeed, if an immune response involves the production of soluble defense-associated molecules, their passage through plasmodesmata could transmit critical information to neighboring (or distant) naïve cells.

Plant immune responses include a decrease in plasmodesmal aperture, which reduces cell-to-cell movement of molecules (as recently reviewed by Wang et al, 2021b; German et al, 2023; Alazem and Burch-Smith, 2024). Plasmodesmal aperture is regulated via callose deposition and degradation at the plasmodesmal neck by callose synthases (CalS) and β-glucanases, respectively (Levy et al, 2007; Vatén et al, 2011). The decrease in plasmodesmal aperture in response to stress is a process activated by signaling cascades mediated by specific machinery. For example, LYSM-CONTAINING GPI-ANCHORED PROTEIN 2 (LYM2; Faulkner et al, 2013) and CALMODULIN-LIKE 41 (CML41; Xu et al, 2017) specifically mediate plasmodesmal responses to the microbial elicitors chitin and flg22, respectively, and PLASMODESMATA LOCATED PROTEIN 5 (PDLP5) integrates these responses, as well as plasmodesmal responses triggered by the defense hormone salicylic acid (SA; Wang et al, 2013; Tee et al, 2023b). The observation that *lym2* mutants cannot close their plasmodesmata in response to chitin but execute canonical chitin-triggered mitogen-activated protein kinase (MAPK) activation and apoplastic ROS production (Faulkner et al, 2013) indicates that plasmodesmal signaling cascades can act independently of other immune responses. This independence of response suggests that whether plasmodesmata are open or closed might be informed by another layer of cellular regulation.

It has been observed that plasmodesmal permeability decreases upon PAMP perception within 30 min (Xu et al, 2017) and is still detected after 24 h (Lim et al, 2016; Li et al, 2021). Both PAMP perception and pathogen infection initiate a wide array of defense responses making it challenging to identify the specific contribution of plasmodesmal closure to immunity. Further, in the context of an infection, many pathogens deploy effectors targeting plasmodesmata that suppress plasmodesmal closure (e.g., Aung et al, 2020; Tomczynska et al, 2020; Li et al, 2021; Ohtsu et al, 2024), further complicating the analysis of the role of plasmodesmata in host immune execution in an infection context.

Whether plasmodesmata can close or not during immune responses determines whether a full defense response can be executed but untangling how plasmodesmal closure contributes to overall immunity is a complex problem. To begin to address this we have simplified the question and asked what cellular responses are triggered by plasmodesmal closure, and how closing plasmodesmata affects elements of an immune response. We generated two genetic tools with which we can induce plasmodesmal closure independently of an immune or stress elicitor. By identifying what responses are trigged by plasmodesmal closure in both tools, we found that plasmodesmal closure itself instigates a subset of stress responses including transcriptional reprogramming and salicylic acid (SA) production. This raises further questions regarding how this contributes to the orchestration of immune responses, how plasmodesmal closure triggers stress responses and whether these stress responses occur during the broad range of physiological processes during which plasmodesmata close.

## Results and discussion

### Genetic tools induce plasmodesmal closure independent of a physiological elicitor

To manipulate plasmodesmal closure independently of external signals we generated two transgenic lines in which estradiol application can induce callose deposition at plasmodesmata. First, we exploited the *icals3m* overactive *CalS3* allele (Vatén et al, 2011) that enhances callose deposition at plasmodesmata; *icals3m* has been used extensively as a tool to understand symplastic connection in development (e.g., Paterlini et al, 2021; Ross-Elliott et al, 2017; Sevilem et al, 2012; Wu et al, 2016; Yadav et al, 2014) but less is known about its effect on immunity. As a callose synthase, this enzyme acts terminally in the plasmodesmal-associated callose deposition pathway. Secondly, we utilized the observation that a synthetic construct of the fluorescent protein mCherry fused between the PDLP1 signal peptide, and the PDLP1 transmembrane domain and cytoplasmic tail, also promotes callose deposition at plasmodesmata (Caillaud et al, 2014). We fused these two transgenes independently to the *XVE* chimeric transcription activator and *LexA* operator (Zuo et al, 2000) and transformed them into *Arabidopsis thaliana* Col-0 to create two different inducible tools, named LexA::icals3m and LexA::PD-Plug, respectively. To identify functional transgenic lines, we performed qPCR, copy number analysis and/or live imaging (Appendix Table S1; Appendix Fig. S1).

**Figure 1:**
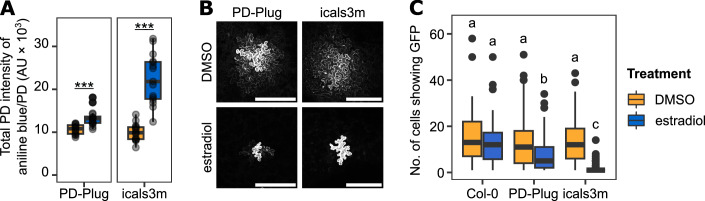
Characterization of plasmodesmal status in induced LexA::PD-Plug and LexA::icals3m tissue. (**A**) Quantification of aniline blue-stained plasmodesmata-associated callose in 5-week-old plants of LexA::PD-Plug (PD-Plug) or LexA::icals3m (icals3m) 24 h post DMSO or estradiol treatment. Datapoints represent the average of the total PD intensity/plasmodesmata (PD) per image, with *n* = 14, 16, 16, and 15 from left to right, respectively. Bootstrap analysis indicates significant differences between DMSO and estradiol treatment in PD-Plug and icals3m, as indicated by *** *p* < 0.001. (**B**) Example micrographs of bombardment sites showing movement of GFP from transformed cells indicate cell-to-cell connectivity of uninduced (DMSO) versus restricted cell connectivity in induced (estradiol) leaves of PD-Plug and icals3m. (**C**) Quantification of microprojectile bombardment data showing GFP movement into neighboring cells. Leaves of 5-week-old plants were bombarded after 24 h treatment of DMSO or estradiol; *n* = 60, 80, 128, 152, 111, and 120 bombardment sites from left to right, respectively. Bootstrap analysis with significant differences indicated by a, b and c, with *p* < 0.01. Scale bar = 200 µM. For (**A**) and (**C**), the center line marks the median, the box indicates the upper and lower quartiles, and the whiskers show the minimum and maximum values within 1.5× interquartile range. Source data are available online for this figure.

**Figure EV1:**
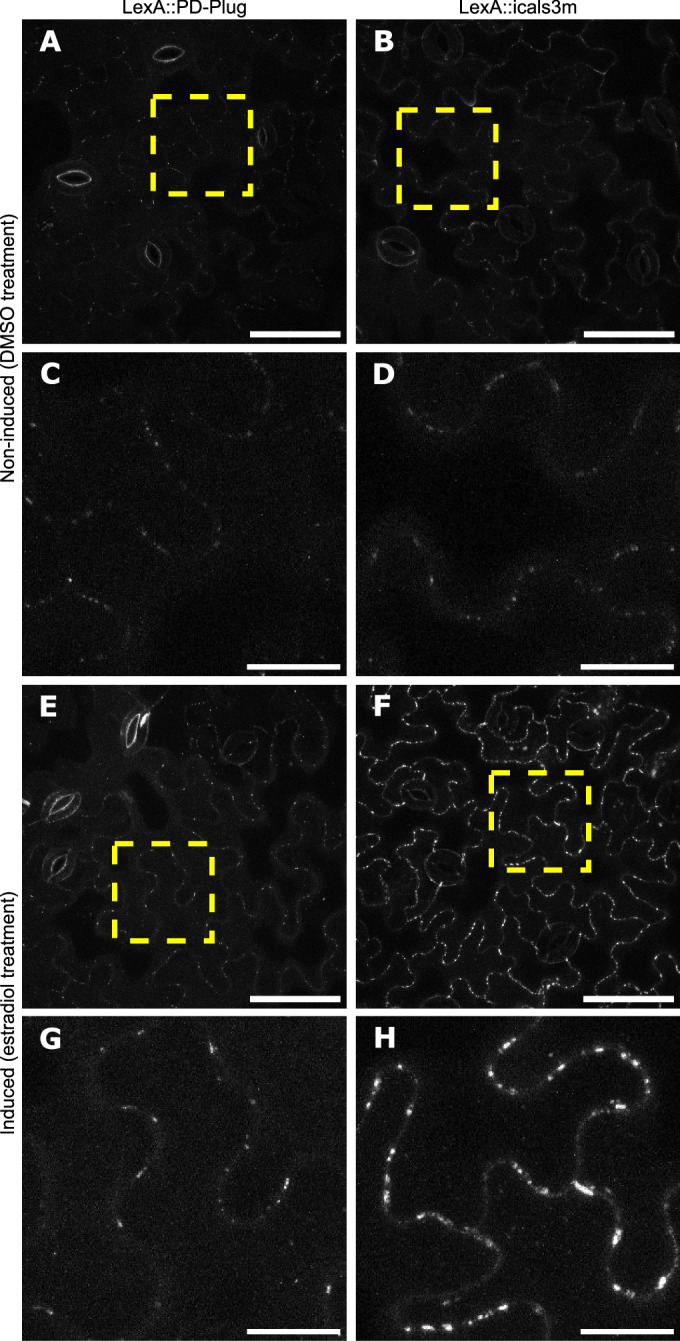
Aniline blue-stained callose at plasmodesmata in LexA::PD-Plug and LexA::icals3m. LexA::PD-Plug and LexA::icals3m treated with either DMSO (**A**–**D**) or estradiol (**E**–**H**) and infiltrated with aniline blue 24 h post treatment. (**C**) and (**D**) are zoomed in portions of (**A**, **B**), and (**G**, **H**) is zoomed in portion of (**E**, **F**) as indicated by the yellow square. (**A**, **B**) and (**E**, **F**) scale bar = 50 µM; (**C**, **D**) and (**G**, **H**) scale bar = 15 µM.

To functionally characterize the selected LexA::PD-Plug and LexA::icals3m lines, we first measured callose deposition by quantitative live cell imaging of aniline blue-stained plasmodesmal callose 24 h (h) post estradiol treatment. At this timepoint, induced LexA::PD-Plug and LexA::icals3m both showed greater accumulation of callose at PD when compared to the DMSO control, although the response in LexA::icals3m (Figs. 1A and EV1) suggests that this line can deposit more callose at plasmodesmata than LexA::PD-Plug. To determine whether increased callose deposition perturbed plasmodesmal permeability, we used microprojectile bombardment assays of *35S::GFP* to measure GFP flux between cells in DMSO or estradiol-treated leaves. While estradiol treatment did not impact GFP flux in comparison to DMSO in wild-type Col-0 plants, we found that estradiol induction of either transgene reduced GFP flux between cells, with the extent of GFP flux being significantly less in induced LexA::icals3m compared to induced LexA::PD-Plug (Fig. 1C). From these results, we conclude that estradiol induces plasmodesmal callose deposition, and plasmodesmal closure in both LexA::PD-Plug and LexA::icals3m.

**Figure EV2:**
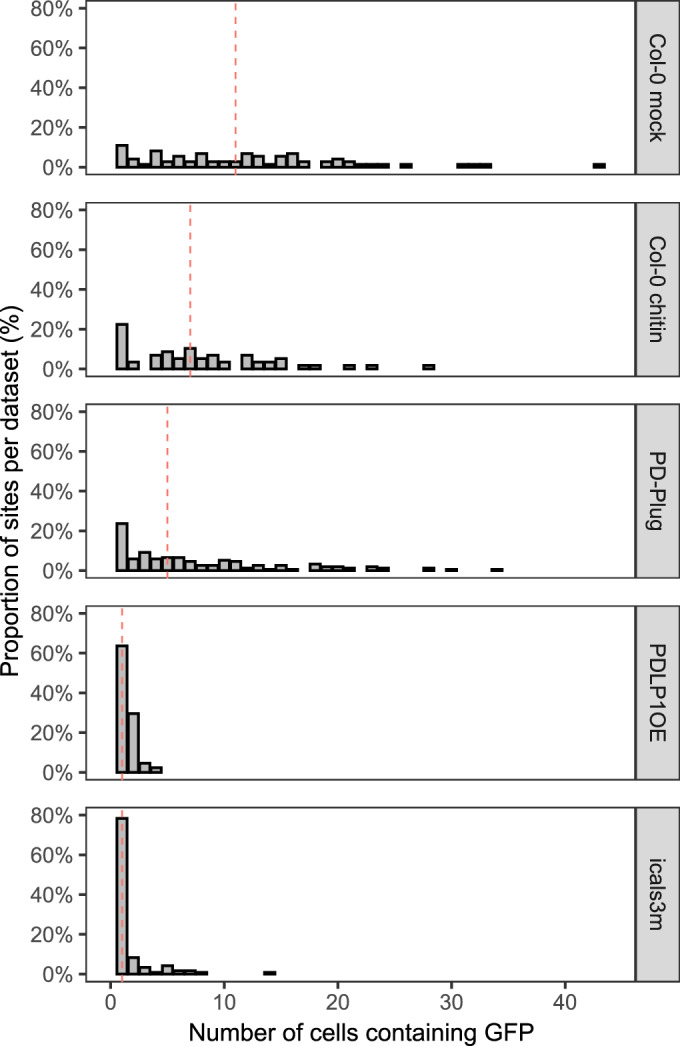
Microprojectile bombardment data in different conditions and genotypes. Comparison of microprojectile bombardment data showing GFP movement into neighboring cells, with datasets showing high variance and heterogeneity (i.e., Col-0 mock, Col-0 chitin and estradiol treated LexA::PD-Plug [PD-Plug]) in comparison to low variance and little GFP movement (i.e., PDLP1OE and estradiol treated LexA::icals3m [icals3m]). Data represented taken from Cheval et al (2020) for Col-0 mock and Col-0 chitin, and Tee et al (2023b) for PDLP1OE. Red dotted line indicates median in a given dataset.

We noted that the data obtained from GFP mobility assays indicates that induced LexA::PD-Plug displays heterogeneous plasmodesmal closure; while there is an increase in the population of cells that exhibit plasmodesmal closure in induced tissues, a subset of the population of cells remain connected to their neighbors (Figs. 1C and EV2). This is reminiscent of the data obtained from GFP mobility assays of leaves treated with elicitors such as chitin and flg22 (Cheval et al, 2020; Tee et al, 2023b). By comparison, induced LexA::icals3m displays a more homogenous response with low variance in cell-to-cell movement (Figs. 1C and EV2), similar to transgenic lines such as the PDLP1-overexpressor (Tee et al, 2023b).

To further characterize the selected LexA::PD-Plug and LexA::icals3m lines, we profiled the timing of plasmodesmal callose deposition across an extended timeline. Significant plasmodesmal callose accumulation following estradiol treatment was first detected at 24 h post treatment in both LexA::PD-Plug and LexA::icals3m, and was sustained up to 72 h post treatment (Appendix Fig. S2). Despite differences in the degree of plasmodesmal closure, these transgenic lines allow us to manipulate plasmodesmata independently of both endogenous and non-endogenous signals and investigate plant responses to plasmodesmal closure.

### Plasmodesmal closure triggers stress transcriptional responses and salicylic acid production

**Figure EV3:**
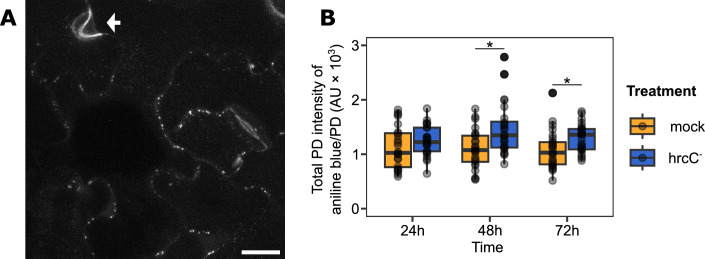
*Pseudomonas syringae* DC3000 mutant strain *hrcC*^-^ infection in Col-0. (**A**) Example aniline blue-stained callose deposition at plasmodesmata as well as macroscopic callose deposits (labeled by white arrow) with the *Pseudomonas syringae hrcC*^*-*^ mutant (*hrcC*^*-*^) treatment. Scale bar = 15 µm. (**B**) Quantification of aniline blue-stained plasmodesmata-associated callose in 5-week-old Col-0 plants 24 h, 48 h, and 72 h post treatment of H_2_O (mock) or *hrcC*^*-*^. Datapoints represent the average of the total PD intensity/plasmodesmata (PD) per image, with *n* = 24–25 images from 8 biological replicates per treatment/timepoint. Bootstrap analysis indicates significant differences between mock and *hrcC*^*-*^ treatment at 48 h and 72 h, as indicated by *p < 0.05. For (**B**), the center line marks the median, the box indicates the upper and lower quartiles, and the whiskers show the minimum and maximum values within 1.5× interquartile range.

As pathogens can manipulate plasmodesmal aperture during infection, it is difficult to determine whether plants sustain immune-associated plasmodesmal closure when challenged with a pathogen over time. Thus, we examined callose deposition in response to infection by *Pseudomonas syringae* DC3000 (*Pst* DC3000) mutant strain *hrcC*^*-*^ that does not secrete effectors that manipulate plasmodesmal aperture. We found that plasmodesmal callose was deposited throughout the leaf at 24 h, 48 h, and 72 h post infection (hpi), and significantly increased at 48 and 72 hpi (Fig. EV3). This suggests that sustained plasmodesmal closure can occur throughout an infection context.

To investigate the impact of sustained plasmodesmal closure on leaf tissue, we next characterized the transcriptional profile of this process using RNAseq, identifying transcriptional changes at 12 h, 24 h, 48 h, and 72 h following DMSO or estradiol treatment in Col-0, LexA::Plug and LexA::icals3m. Preliminary analysis of the data first revealed that a critical factor of variance was time (Appendix Fig. S3). Therefore, all subsequent analyses were performed within single timepoints. We assessed the effect of estradiol relative to DMSO treatment within a genotype, identifying differentially expressed genes (DEGs). The greatest response was in Col-0 at 12 h, where estradiol led to the downregulation of over a thousand genes (Appendix Fig. S4). We checked whether estradiol influenced plasmodesmal-associated callose deposition in Col-0, but no change was detected (Appendix Fig. S5). The presence of shared estradiol-responsive DEGs in Col-0 and the transgenic genotypes supports the possibility of an early estradiol specific response not correlated to transgene induction. The number of these shared estradiol-induced DEGs detected was dramatically reduced after 24 h (Appendix Fig. S4; Dataset EV1), suggesting secondary transcriptional effects of estradiol are negligible after this point.

To further validate our tools, we assessed the transgene induction across the sampling period; both PD-Plug transcripts (as indicated by read counts of mCherry) and icals3m transcripts (as indicated by read counts of CalS3) were upregulated compared to controls by 12 h post estradiol treatment and remained significantly elevated at 72 h (Appendix Fig. S6). To determine that the upregulation of the transgenes did not induce ER stress from high levels of ectopic protein production, we examined the expression of genes known to be ER stress markers, defined as either ER stress sensors or unfolded protein response (UPR) effectors (Beaugelin et al, 2020). Of the 13 genes examined, only *BIP3* (AT1G09080) was significantly upregulated in estradiol treated LexA::PD-Plug and LexA::icals3m at 72 h (Appendix Fig. S7), suggesting that transgene expression in these lines does not induce ER-stress. We also investigated gene expression changes specific to the induction of each transgene by using a likelihood-ratio test (Appendix Fig. S8, Dataset EV2). At both 12 h and 24 h, we found no genes uniquely upregulated in the estradiol treated samples from either LexA::PD-Plug or LexA::icals3m. At 48 h, 28 and five genes were uniquely upregulated in estradiol treated LexA::PD-Plug and estradiol treated LexA::icals3m, respectively. At 72 h, 33 genes were differentially upregulated in estradiol treated LexA::icals3m alone; there were no genes differentially upregulated exclusively in estradiol treated LexA::PD-Plug at 72 h.

**Figure 2:**
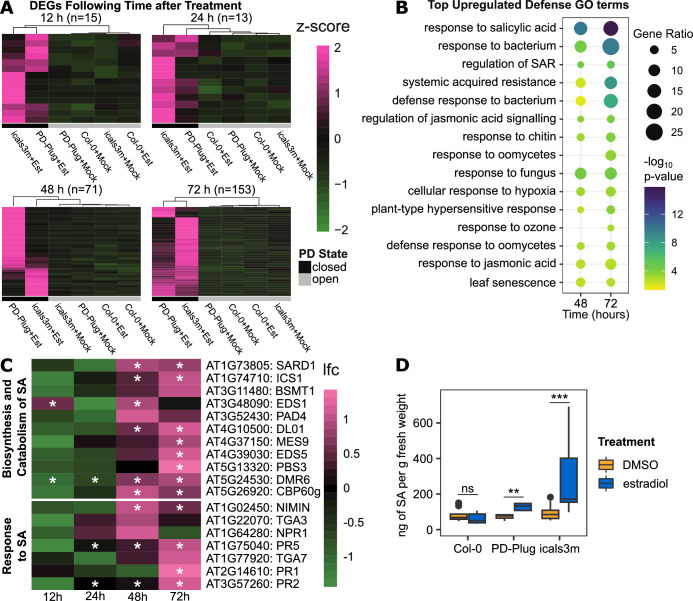
Plasmodesmal closure triggers stress transcriptional responses and salicylic acid-related changes. (**A**) Likelihood ratio test analysis determined number (indicated by *n*) of differentially expressed genes (DEGs) significantly differed between plasmodesmal status (i.e., PD State closed or open), and matches the cluster pattern of upregulated in closed state in comparison to open state at 12, 24, 48, and 72 h post treatment. Hierarchical clustering grouped together plasmodesmal state of open (i.e., estradiol treated LexA::PD-Plug and LexA::icals3m) and closed (DMSO treated LexA::PD-Plug, LexA::icals3m and Col-0, and estradiol treated Col-0). Expression normalized by row (z-score). (**B**) Top 15 defense GO terms enriched over time when plasmodesmata are closed, using a Fisher’s exact test cut off of *p* < 0.05. (**C**) Expression of genes related to the salicylic acid biosynthesis/catabolism and response over time when plasmodesmata are closed (i.e., in estradiol treated LexA::PD-Plug and LexA::icals3m; Dataset EV7). (**D**) Quantification of salicylic acid in 5-week-old plants after 72 h DMSO or estradiol treatment, *n* = 12, 12, 6, 6, 12, and 12 from left to right, respectively. Significant differences between treatment within a genotype analyzed using Mann–Whitney test denoted by ns *p* = 0.14, **p = 0.002, or ***p < 0.001. The center line marks the median, the box indicates the upper and lower quartiles, and the whiskers show the minimum and maximum values within 1.5× interquartile range. Source data are available online for this figure.

While both the icals3m and PD-Plug proteins have different modes of action, we reasoned that the genes that are commonly up- or down-regulated following induction are associated with their common effect on plasmodesmata and likely to indicate core responses to plasmodesmal closure. To identify genes uniquely upregulated when plasmodesmata are closed, we defined two different groups for comparison: plasmodesmal state closed (estradiol treated LexA::PD-Plug and LexA::icals3m) and plasmodesmal state open (DMSO treated LexA::PD-Plug and LexA::icals3m, and all Col-0 samples). A likelihood-ratio test was performed on these two groupings at each timepoint to identify DEGs expressed only in the closed plasmodesmal state (Fig. 2A; Appendix Fig. S9; Datasets EV3–6) and observed that the number of upregulated DEGs increased over time, suggesting that the longer plasmodesmata remain closed, the greater the cellular response. Notably, the number of DEGs that was shared between LexA::icals3m and LexA::PD-Plug in the grouping of plasmodesmal state closed was greater than the number of upregulated DEGs specific to each line (Fig. 2A). A GO term analysis identified that genes upregulated when plasmodesmata are closed at 48 and 72 h are enriched in defensive processes (Fig. 2B; Appendix Fig. S10).

As response to SA at 72 h was the most significantly enriched GO term in the entire analysis, we further explored this by analyzing SA-related DEGs to determine what aspects of SA signaling were impacted when plasmodesmata were closed (Fig. 2C; see Appendix Table S2 for associated references; Vlot et al, 2008; Seyfferth and Tsuda, 2014; Yang et al, 2015; van Butselaar and Van den Ackerveken, 2020). Both SA biosynthesis/catabolism and SA response pathways were significantly upregulated at 48 and 72 h. To confirm that the transcriptional signature of SA is a consequence of the activation of SA synthesis when plasmodesmata are closed, we measured SA content in Col-0, LexA::PD-Plug and LexA::icalsm3 72 h post DMSO and estradiol treatment. We found induced plasmodesmal closure increased SA content in both LexA::PD-Plug and LexA::icals3m, with induced LexA::icals3m having a greatly increased SA content, being three-fold higher when compared to DMSO treatment (Fig. 2D). It has long been known that constitutive over-production of the plasmodesmal protein PDLP5 closes plasmodesmata and leads to high levels of SA (Wang et al, 2013). Here, we have demonstrated that SA accumulation in response to plasmodesmal closure is not solely dependent on *PDLP5*. Overall, plasmodesmal closure leads to the upregulation of transcriptional defense responses, and SA synthesis and accumulation, independent of an immune stimulus.

Previous studies have shown pathogen-induced SA accumulation is associated with the suppression of jasmonic acid (JA) signaling (e.g., Spoel et al, 2003). Therefore, we might expect JA abundance and JA signaling to be suppressed when plasmodesmata are closed. However, GO terms associated with JA signaling and response were enriched amongst DEGs associated with 48 and 72 h PD closure (Fig. 2B; Appendix Fig. S10), and we could not quantify any differences in JA content in tissue when PD were closed (Appendix Fig. S11), suggesting direct antagonism between SA and JA is not occurring in this context.

### Plasmodesmal closure differentially impacts pathogen resistance and immune responses

**Figure 3:**
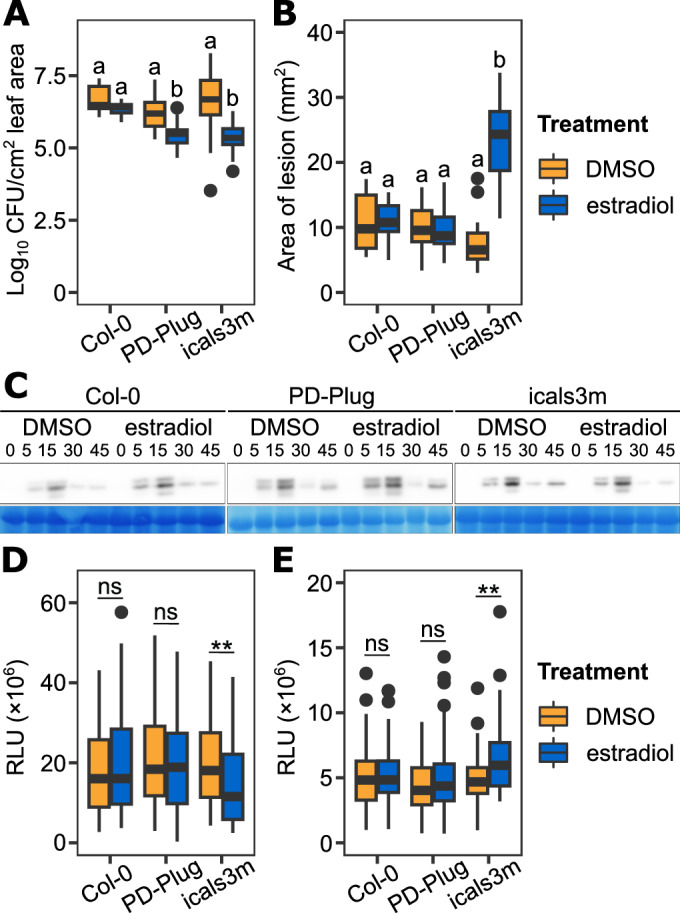
Plasmodesmal closure differentially impacts pathogen resistance and immune responses. (**A**) Bacterial growth (log_10_ CFU/cm^2^ leaf area) of *Pst* DC3000 3 dpi on uninduced and induced tissue of 4–5-week-old plants of Col-0, LexA::PD-Plug (PD-Plug) and LexA::icals3m (icals3m). Tissue was treated with DMSO (uninduced) or estradiol (induced) 72 h prior to infection. Four independent experiments were conducted, with 5 plants per treatment/genotype; *N* = 20. Independent factors genotype and treatment were significant, with a significant interaction between the two (ANOVA; F = 16.6, df = 2, *p* < 0.001; F = 67.0, df = 1, *p* < 0.001; F = 8.2, df = 2, *p* < 0.001). Significant differences denoted by a and b, *p* < 0.001; see Dataset EV8 for specific *p* values. (**B**) The average area of *Botrytis cinerea* disease lesions 2 dpi in leaves of 5-week-old plants of induced and uninduced Col-0, LexA::PD-Plug and LexA::icals3m. Tissue was treated with DMSO (uninduced) or estradiol (induced) 72 h prior to infection, with *N* = 16–17 for all genotypes/treatment combined from three independent repeats. Independent factors genotype and treatment were significant with a significant interaction between the two (ANOVA; F = 24.4, df = 2, *p* < 0.001; F = 52.7, df = 1, *p *< 0.001; F = 57.0, df = 2, *p* < 0.001). Significant differences denoted by a and b, *p* < 0.001; see Dataset EV8 for specific *p* values. (**C**) Western blot detection of MAPK-activation by flg22 treatment in leaf discs from expanded leaves of 4–5-week-old plants of Col-0, LexA::PD-Plug and LexA::icals3m collected 72 h post DMSO or estradiol treatment. Minute timings of tissue harvest are denoted, and Coomassie blue staining shown below as loading controls. (**D**) Average relative light units (RLU) generated from ROS production in response to flg22 in leaf discs of 5-week-old plants. Leaves were treated with estradiol or DMSO 72 h prior to flg22 treatment and data is combined from two independent experiment with *N* = 95 per genotype/treatment. Significant differences between treatment determined by bootstrap analysis, with significant differences denoted by **p < 0.01. (**E**) Average RLU generated from ROS production in response to flg22 in seedlings. Plants were treated with estradiol or DMSO 72 h prior to flg22 treatment, with *n* = 89, 93, 88, 80, 94 and 91 from left to right, respectively. Significant differences between treatment determined by bootstrap analysis, with significant differences denoted by **p < 0.01. For (**A**), (**B**), (**D**) and (**E**), the center line marks the median, the box indicates the upper and lower quartiles, and the whiskers show the minimum and maximum values within 1.5× interquartile range. Source data are available online for this figure.

Plants that lack plasmodesmata-specific immune signaling machinery and cannot close their plasmodesmata in response to pathogen infection, show increased susceptibility to different pathogenic microbes (Lee et al, 2011; Faulkner et al, 2013; Xu et al, 2017). To determine whether an inverse correlation exists for induced plasmodesmal closure we examined the susceptibility of induced, transgenic lines to the bacterial, biotrophic pathogen *Pst* DC3000. We inoculated expanded leaves of Col-0, LexA::PD-Plug and LexA::icals3m plants 72 h post DMSO or estradiol treatment (Fig. 3A) and found that induction of both transgenes reduced bacterial growth 3 days post infection (dpi). Thus, plasmodesmal closure is correlated with enhanced resistance to *Pst* DC3000. Next, we assayed for resistance to the fungal necrotroph, *Botrytis cinerea*. In contrast with *Pst* DC3000 infection, we found induction of the PD-Plug transgene had no impact on *B. cinerea* infection when compared to the disease caused in Col-0 plants, but induction of *icals3m* increased susceptibility to *B. cinerea* (Fig. 3B; Appendix Fig. S12). This indicates that plasmodesmal closure specifically does not lead to the enhancement of resistance in all infection contexts with the differences observed between susceptibility to a necrotroph and a biotroph raising the possibility that the effects of plasmodesmal closure differentially interact with different lifestyles of different microbes.

Overall susceptibility is driven by multiple components of host defense responses, and plasmodesmal closure is a key immune response to the pathogen-associated molecular patterns (PAMPs) flg22 and chitin. To determine if plasmodesmal closure in our transgenic lines influences other PAMP responses, we assessed the activation of MAPK signaling and ROS production by flg22 in induced LexA::icals3m and LexA::PD-Plug. When we assayed for flg22-triggered MAPK phosphorylation in leaf discs of mature leaves and intact seedlings of both lines following estradiol treatment, we saw no difference in the timing or activation of the response when compared to Col-0 (Fig. 3C; Appendix Fig. S13). By contrast, while induction of *LexA::PD-Plug* did not perturb the flg22-triggered ROS burst in either mature leaves or seedlings (Fig. 3D,E), when we assayed for the same response in LexA::icals3m, induction of the transgene reduced the flg22-triggered ROS burst in mature leaves (Fig. 3D) and increased the response in seedlings (Fig. 3E). To determine whether response differences are underpinned by differences in basal cellular ROS levels, we stained estradiol treated Col-0 and LexA::icals3m leaves with H_2_DCFDA but observed no quantitative differences between the genotypes or treatments (Appendix Fig. S14). Given that our *Pst* DC3000 assays were performed in mature leaves, this suggests that increased resistance induced by plasmodesmal closure is not associated with flg22-induced MAPK or ROS responses.

### Plasmodesmal closure induces sugar accumulation and growth defects

**Figure 4:**
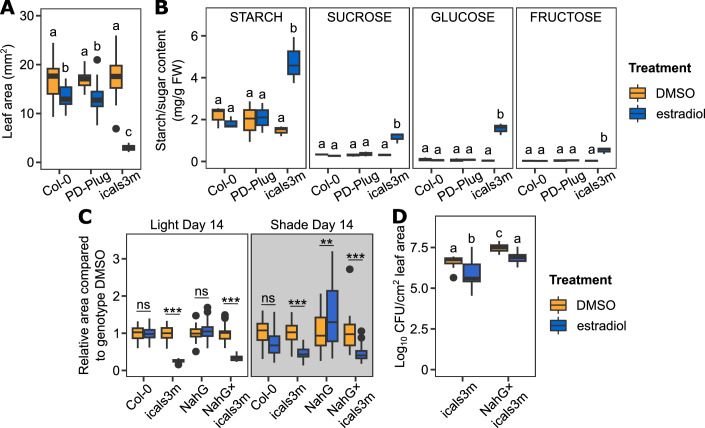
Plasmodesmal closure induces sugar accumulation and impairs leaf growth. (**A**) Leaf area of first two true leaves 6 days post treatment (DMSO or estradiol) of Col-0, LexA::PD-Plug (PD-Plug) and LexA::icals3m (icals3m). Independent factors genotype and treatment were significant, with a significant interaction between the two (ANOVA; F = 82.4, df = 2, *p* < 0.001; F = 445.0, df = 1, *p* < 0.001; F = 104.2, df = 2, *p* < 0.001). Significant differences denoted by a, b and c, *p* < 0.001. *n* = 40. (**B**) Starch, sucrose, glucose and fructose content in leaves of 5-week-old plants 72 h post DMSO or estradiol treatment. N = 3. Independent factors genotype and treatment were significant, with a significant interaction between the two for each metabolite (ANOVA; *starch* F = 4.7, df = 2, *p* < 0.05, F = 9.2, df = 1, *p* < 0.05; F = 11.4, df = 2, *p* < 0.01; *sucrose* F = 26.2, df = 2, *p* < 0.001; F = 25.8, df = 1, *p* < 0.001; F = 26.7, df = 2, *p* < 0.001; *glucose* F = 70.0, df = 2, *p* < 0.001; F = 74.4, df = 1, *p* < 0.001; F = 79.3, df = 2, *p* < 0.001; *fructose* F = 28.0, df = 2, *p* < 0.001; F = 30.4, df = 1, *p* < 0.0001; F = 28.8, df = 2, *p* < 0.001). Significant differences denoted by a and b, *p* < 0.01; see Dataset EV8 for specific *p* values. (**C**) Relative leaf area of true leaves 14 days post initial treatment (DMSO or estradiol) in light or shade conditions of Col-0, icals3m, NahG and NahG×LexA::icals3m (NahG×icals3m). *N* = 30, 31, 30, 31, 26, 31, 30, 30, 29, 29, 30, 26, 26, 26, 30, 24 from left to right, respectively. For relative values, independent factors treatment and genotype were significant while shade was not; there was a significant interaction between some of the pairs and between all three (ANOVA; F = 25.7, df = 1, *p* < 0.001; F = 56.9, df =3, *p* < 0.001; F = 0.6, df = 1, *p* = 0.4; Treatment:Genotype F = 57.3, df = 3, *p* < 0.001; Treatment:Shade F = 0.9, df = 1, *p* = 0.35; Genotype:Shade F = 6.2, df = 3, *p* < 0.001; Treatment:Genotype:Shade F = 7.1, df = 3, *p* < 0.001). Significant differences denoted by **p < 0.01, and ***p < 0.001; see Dataset EV8 for specific indicated *p* values. (**D**) Bacterial growth (log_10_ CFU/cm^2^ leaf area) of *Pst* DC3000 3 dpi on uninduced and induced tissue of 4–5-week-old plants. Tissue was treated with DMSO (uninduced) or estradiol (induced) 72 h prior to infection. Four independent experiments were conducted, with 5 plants per treatment/genotype; *N* = 20. Independent factors treatment and genotype were significant, with no significant interaction between the two (ANOVA; F = 42.6, df = 1, *p* < 0.001; F = 75.8, df = 1, *p* < 0.001; F = 0.4, df = 1, *p* = 0.5). Significant differences denoted by a, b and c, *p* < 0.001; see Dataset EV8 for specific *p* values. For all panels, the center line marks the median, the box indicates the upper and lower quartiles, and the whiskers show the minimum and maximum values within 1.5× interquartile range. Source data are available online for this figure.

**Figure EV4:**
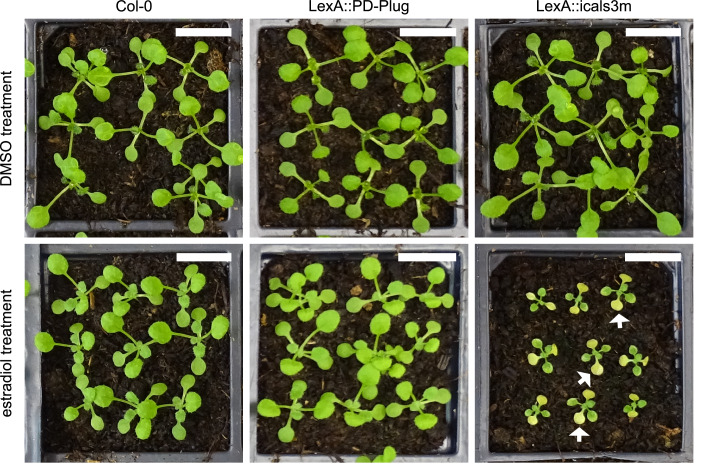
Photographs of 15-day-old plants showing the growth phenotype of Col-0, LexA::PD-Plug, LexA::icals3m induced by DMSO or estradiol treatment. White arrows indicate yellowing/senescence on leaves. Scale bar = 15 mm.

As plasmodesmata connect the phloem to cells in young sink tissues and allow sugar translocation, we expect that plasmodesmal closure might reduce the flux of sugars to growing tissues and limit growth. Alternatively, activation of defense processes can negatively regulate growth and therefore it is possible that plasmodesmal closure impairs growth by one or both pathways. Indeed, several transgenic lines known for constitutive plasmodesmal closure such as *PDLP1, PDLP5* and *PDLP6* overexpressors (Thomas et al, 2008; Lee et al, 2011; Li et al, 2024) exhibit a significant retardation in growth. Thus, we explored whether sustained plasmodesmal closure reduced growth in induced LexA::PD-Plug and LexA::icals3m. In 9-day-old seedlings, we applied either DMSO or estradiol treatment and measured the first true leaves (Fig. 4A) six days post treatment. We found that while estradiol reduced leaf growth in both Col-0 and LexA::PD-Plug in comparison to the DMSO treated seedlings, the estradiol-induced growth reduction was greater in LexA::icals3m relative to LexA::PD-Plug and Col-0. Alongside reduced leaf size, we observed yellowing and chlorosis on the cotyledons of estradiol treated LexA::icals3m (Fig. EV4).

Plasmodesmal closure might be expected to restrict photoassimilate distribution; a reduction in plasmodesmal permeability has been documented in *Arabidopsis* alongside a significant accumulation of soluble sugars and starch (e.g., in the MOVEMENT PROTEIN 17 *MP17* overexpressor, *CHOLINE TRANSPORTER-LIKE 1 cher1* mutant, *PDLP5* and *PDLP6* overexpressors; Kronberg et al, 2007; Kraner et al, 2017; Li et al, 2024). Therefore, we quantified sugar accumulation following plasmodesmal closure, measuring the sucrose, fructose, glucose, and starch content of source leaves 72 h post treatment. These data reveal a significant increase in all four sugars in estradiol treated LexA::icals3m samples (Fig. 4B). This increase in sugar content, combined with the increase in SA might explain our pathoassay data; the necrotrophic pathogen *B. cinerea* might be able to access increased intracellular sugar stores and be unimpeded by SA defense pathways (Ferrari et al, 2003; El-Oirdi et al, 2011), while *Pst* DC3000 might be unable to access intracellular sugars and is impeded by SA (Velásquez et al, 2017; Wilson et al, 2017; Howlader et al, 2020).

Sugars can induce specific signal transduction pathways, with some studies finding that increasing source leaf carbohydrate content leads to a feedback loop of decreasing photosynthetic yield (Araya et al, 2006). Reasoning that an increase in sugar content might down-regulate photosynthesis, we used chlorophyll fluorescence imaging to infer photosynthetic yield and found there was no difference in photosynthetic yield of PSII (as indicated by Y(II)) after 72 h in Col-0, LexA::PD-Plug and LexA::icals3m between the DMSO or estradiol treatments (Table 1).

**Table 1:** Photosynthetic yield as indicated by Y(II) following DMSO or estradiol treatment of leaves of Col-0, LexA::PD-Plug (PD-Plug) and LexA::icals3m (icals3m).

	Col-0	PD-Plug	icals3m
DMSO	0.79 ± 0.02	0.78 ± 0.02	0.78 ± 0.02
Estradiol	0.79 ± 0.01	0.78 ± 0.01	0.79 ± 0.01

Pooled average of Y(II) ± SD, with a biological replicate data averaged from 4 leaves per treatment/genotype on a given experimental run where *n* = 8, combined from two independent experiments. Independent factors genotype and treatment were not significant, with no significant interaction between the two (ANOVA; F = 1.3, df = 2, *p* = 0.29; F = 1.2, df = 1, *p* = 0.29, F = 0.1, df = 2, *p* = 0.92).

As there were no changes in the photosynthetic yield that correlated with the increase in sugar content in induced LexA::icals3m, we next hypothesized that plasmodesmal closure and sugar accumulation might activate the expression of sugar transporters and starch biosynthesis genes to reduce the cytosolic concentration of soluble sugars. An inverse relationship between plasmodesmal aperture and the expression/activity of sugar transporters has been reported (Ruan et al, 2001; Zhang et al, 2017a; Liu et al, 2022) suggesting that cells maintain the capability to transport sugars between cells by balancing plasmodesmal aperture with transporter activity (Zhang et al, 2017b). However, out of 125 genes known to be sugar and starch related (Dataset EV9; Büttner and Sauer, 2000; Williams et al, 2000; Chen et al, 2010; Monroe and Storm, 2018; Abt and Zeeman, 2020; Preiser et al, 2020; Smith and Zeeman, 2020; David et al, 2022; Bavnhøj et al, 2023), only the transporter *SENESCENCE-ASSOCIATED GENE 29 (SAG29)/SUGARS WILL EVENTUALLY BE EXPORTED TRANSPORTERED 15 (SWEET15)* was significantly upregulated when plasmodesmata are closed in LexA::icals3m (Appendix Figs. S15 and S16). AT3G20460 is proposed to encode a monosaccharide transporter (Johnson et al, 2006) and was downregulated in estradiol-treated LexA::icals3m. However, as the function of this gene is not yet well characterized it is difficult to speculate any possible role in the sugar/starch phenotype (Johnson et al, 2006). These results suggest that plasmodesmal closure induces starch and soluble sugar accumulation, but that neither photosynthesis nor sugar transporters significantly offsets this.

**Figure EV5:**
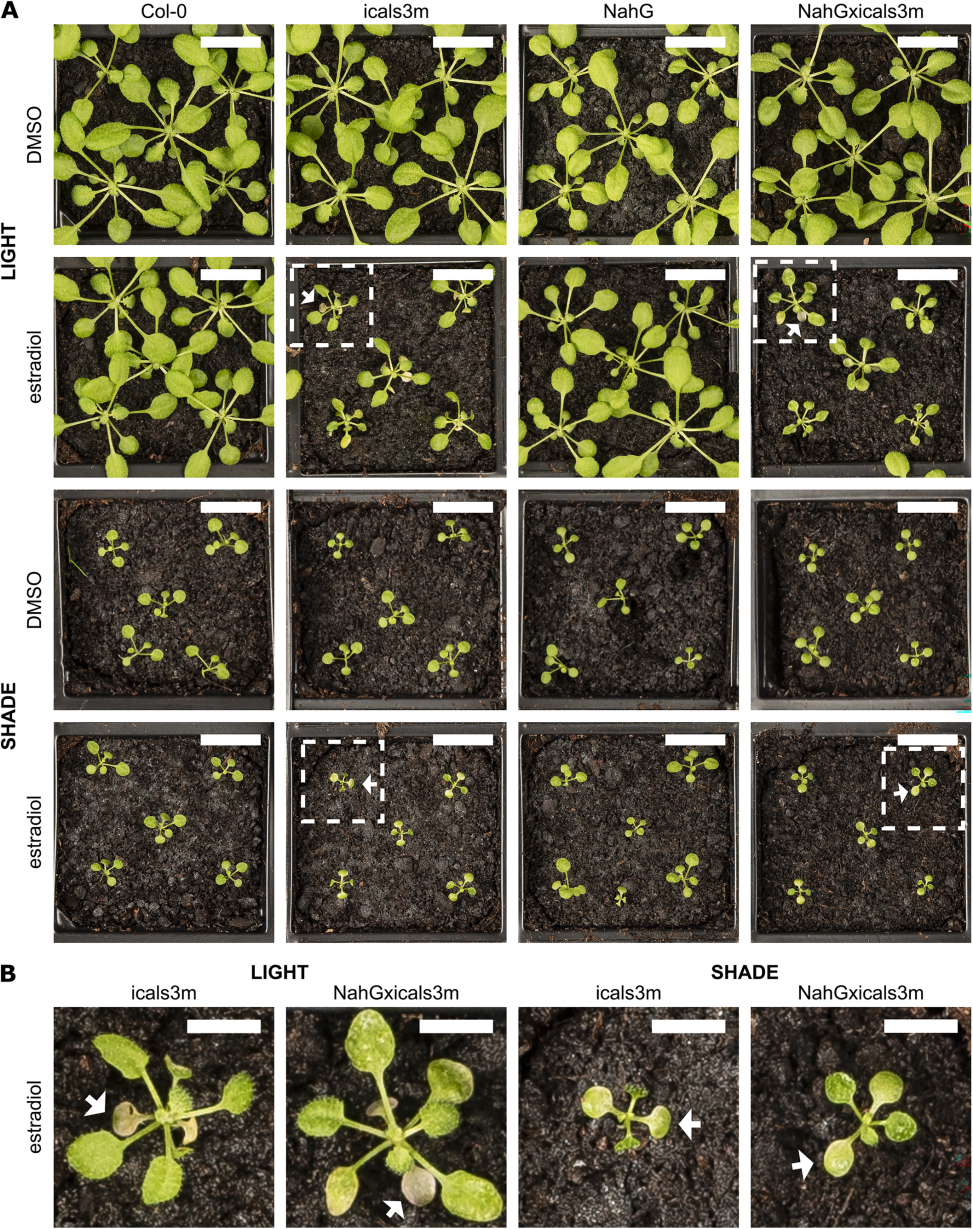
Photographs of 24-day-old plants showing the growth phenotype of Col-0, LexA::icals3m (icals3m), NahG and NahG×LexA::icals3m (NahG×icals3m) induced by DMSO or estradiol treatment under light or shaded conditions. White arrows indicate example yellowing/senescence on leaves. (**B**) is zoomed in portion of (**A**) as indicated by the white square. (**A**) scale bar = 15 mm, (**B**) scale bar = 5 mm.

We reasoned that sugar accumulation might induce osmotic stress which in turn induces the growth and senescence phenotypes of LexA::icals3m. To test this we simultaneously induced plasmodesmal closure and lowered the light intensity by shading the plants to reduce photosynthesis and thus starch and sugar content (Law et al, 2018). Shade conditions reduced growth in all plants, even Col-0, compared to the light grown plants after 14 days of this regime (Fig. EV5). However, estradiol treated LexA::icals3m were smaller than DMSO treated plants (Fig. 4C). While senescence of cotyledons was also still evident, it was not as severe as in normal light conditions (Fig. EV5). This suggests that while plasmodesmal closure induces starch and sugar accumulation, this does not impair growth or singularly induce senescence, and that these phenotypes are regulated by other consequences of plasmodesmal closure.

### SA does not mediate plasmodesmal-induced effects on growth and resistance

Given we detected that plasmodesmal closure increases SA and this could induce the observed senescence and reduction in growth in LexA::icals3m, we sought to test if there is a causal relationship. To test the hypothesis that SA accumulation reduces size and/or induces senescence, we crossed LexA::icals3m with plants that constitutively express *NahG*, the bacterial SA hydroxylase that degrades SA (Gaffney et al, 1993; Delaney et al, 1994; Friedrich et al, 1995; NahG×icals3m). We found similar transcriptional expression of *NahG* in the NahG×icals3m cross to NahG, indicating no transgene silencing in the resulting cross (Appendix Fig. S17). We observed a significant reduction in leaf size as well as senescence observed in the cotyledons after 14 days post plasmodesmal closure in both LexA::icals3m and NahG×icals3m plants (Figs. 4C and EV5), indicating that SA accumulation is not the cause of these phenotypes. Like LexA::icals3m, NahG×icals3m plants showed reduced growth and mild cotyledon senescence when grown in shade conditions, suggesting that SA does not contribute to the effects of plasmodesmal closure observed in these conditions.

Lastly, we used the NahG×icals3m cross to investigate whether enhanced pathogen resistance to *Pst* DC3000 when plasmodesmata are closed is mediated by accumulation of SA. Thus, we inoculated LexA::icals3m and NahG×icals3m plants 72 h post DMSO or estradiol treatment (Fig. 4D) and found DMSO treated NahG×icals3m had the highest level of bacterial growth 3 days post infection (dpi). However, estradiol treatment of both LexA::icals3m and NahG×icals3m reduced bacterial growth suggesting that SA accumulation is not the primary cause of the increased resistance induced by plasmodesmal closure.

Overall, our data suggests that cellular isolation is a stress-inducing state and, conversely, that connection to neighboring cells is essential for optimal cellular function. Understanding how a cell responds when it becomes isolated from its neighbors, and why cell-to-cell connectivity regulates homeostatic processes, is key to understanding how plants function as multicellular organisms.

## Methods

**Table:** Reagents and tools table

Reagent/Resource	Reference or Source	Identifier or Catalog Number
**Experimental models**		
*Arabidopsis thaliana* Col-0 seeds	NASC	N22681
*Arabidopsis thaliana* NahG seeds	Jurriann Ton, The University of Sheffield, UK	n/a
*Arabidopsis thaliana* *LexA::PD-Plug*	This study	n/a
*Arabidopsis thaliana* *LexA::icals3m*	Bellandi et al, 2022; NASC	N2111615
**Recombinant DNA**		
PDLP1 transmembrane domain and cytoplasmic tail	Caillaud et al, 2014	n/a
pB7WG2.0.RFP_ER_	Thomas et al, 2008	n/a
pB7WG2.0.GFP	Thomas et al, 2008	n/a
**Antibodies**		
p44/42 MAPK	Cell Signaling Technologies	#9102
α-Rabbit-HRP	Sigma-Aldrich	A0545
**Oligonucleotides and other sequence-based reagents**		
qPCR primers icals3m/CalS3	This study	n/a
qPCR primers AtUBQ10	This study	n/a
qPCR primers GAPC2	Tee et al, 2023b	n/a
Primers NahG	Heck et al, 2003	n/a
**Chemicals, Enzymes and other reagents**		
β-Estradiol 17-acetate	Sigma-Aldrich	E7879
RNeasy® Plant Mini Kit	QIAGEN	74904
TURBO DNA-*free*^TM^	Invitrogen/Thermo Fisher	10792877/AM1907
High-Capacity cDNA Reverse Transcription Kit	Thermo Fisher	4374966
LightCycler® 480 SYBR® Green I Master	Roche	04707516001
Flagelin 22 (flg22)	EZBiolab/Biosynth	Crb1000331
cOmplete^TM^ ULTRA Tablets, protease inhibitor cocktail	Roche/Sigma-Aldrich	06538282001
PhosSTOP^TM^ phosphatase inhibitor	Roche/Sigma-Aldrich	4906845001
L-012 sodium salt	Sigma-Aldrich	SML2236
Immun-Blot® PVDF Membrane	Bio-Rad	1620177
HRP (peroxidase from horseradish)	Sigma-Aldrich	P6782
H_2_DCFDA	Thermo Fisher	D399
Salicylic acid-_d4_	Merck	S-042
Jasmonic acid-_d5_	Cayman Chemicals	29076
**Software**		
ImageJ	https://imagej.net/ij/index.html	n/a
RStudio	https://posit.co/download/rstudio-desktop/	2021.09.01 Build 351/R version 4.0.3.
TargetLynx Software	Waters	V4.1 SCN876
ImagingWin Software	Walz	n/a
**Other**		
2010 Geno/Grinder®	SPEX® SamplePrep, USA	n/a
LSM Zeiss 800 confocal microscope	Zeiss	n/a
Leica M205 FA stereo microscope	Leica	n/a
Axio Zoom V16 light microscope	Zeiss	n/a
Leica TCS SP5	Leica	n/a
Varioskan Flash	Thermo Fisher	n/a
AQUITY UPLC/Xevo TQS tandem mass spectrometer	Waters	n/a
2.6 µM Kinetex EVO C18 column	Phenomenex	n/a
Imaging-PAM chlorophyll fluorometer	Walz	n/a

### Plant material

All *Arabidopsis thaliana* used in this study is in the Columbia-0 ecotype background. For assays on mature plants, *Arabidopsis* plants were grown at 10 h light at 22 °C, on soil or for the RNA-seq experiment, on Murashige and Skoog (MS) 1% sucrose 0.8% agar in 50 mm diameter petri dishes for 4–5 weeks. For MAPK and ROS measurement assays on seedlings, seeds were germinated on MS 1% sucrose 0.8% agar at 16 h light at 22 °C and grown for 8 days, then 10 seedlings were transferred per well on a 6-well plate together with 8 mL of liquid MS and grown for an additional 7 days.

### Confocal microscopy

Microprojectile bombardment, transgene induction visualization and aniline blue plasmodesmal callose measurements utilized confocal microscopy (LSM Zeiss 800 or Leica TCS SP5). RFP/mCherry was excited with a 561-nm DPSS laser and collected at 600 to 640 nm, GFP/H_2_DCFDA was excited with a 488-nm argon laser and collected at 505 to 530 nm, and aniline blue was excited with a 405-nm UV laser and collected at 430 to 550 nm. Transgene induction visualization was imaged using a 40× oil objective (HCX PL APO CS 40.0×1.25 OIL).

### Microprojectile bombardment

Microprojectile bombardment of *35S::GFP* was performed as described in (Tee et al, 2022). 24 h after DMSO or estradiol treatment, 5-week-old leaves were co-bombarded with pB7WG2.0.RFP_ER_ and pB7WG2.0.GFP. Bombardment sites were then imaged 16 h after using confocal microscopy with a 20× water dipping objective (W N-Achroplan 20×/0.5) or a 25× water dipping objective (HCX IRAPO L 25.0×0.95).

### Generation of transgenic lines and induction of transgene

The *LexA::icals3m* construct is as described (Bellandi et al, 2022). The *LexA::PD-Plug* construct was assembled into a binary vector via Golden Gate cloning; first the ‘PD-Plug’ sequence was created by fusing the PDLP1 signal peptide and the PDLP1 transmembrane domain and cytoplasmic tail to the N- and C-termini of mCherry, respectively (Caillaud et al, 2014). This synthetic peptide was inserted downstream of the *LexA* promoter sequence and upstream of a NOS terminator and assembled into the backbone of pICSL0022013 containing the *Nos::BAR::Nos* selection cassette and *Act2::XVE::Act2* cassette.

*LexA::PD-Plug* and *LexA::icals3m* constructs were transformed into the Col-0 *Arabidopsis* background and screened with phosphinothricin (10 µg/mL concentration) or kanamycin selection (50 µg/mL concentration), respectively. For induction of the transgene in all experiments (with the exception of size phenotyping), leaves were painted on both the abaxial and adaxial side with either 0.1% DMSO (mock treatment) or 20 µM β-Estradiol 17-acetate (estradiol treatment, Sigma-Aldrich) containing 0.01% Silwet L-77 in ddH_2_O, using a fine paint brush. For size phenotyping, seedlings were sprayed with the above specified DMSO or estradiol treatment.

### Plasmodesmal callose measurements

24 h post treatment with either DMSO or estradiol, expanded leaves of 5-week-old plants were infiltrated with 1% aniline blue in PBS buffer (pH 7.4) then imaged using confocal microscopy from the abaxial side using a 63× water immersion objective (Plan-Apochromat 63×/1.20). Z-stacks from multiple areas from a given treated leaf, with a minimum of three biological replicates per genotype and treatment were collected. An automated image analysis pipeline to quantify aniline blue-stained plasmodesmata is available at (Johnston et al, 2022; 10.5281/ZENODO.6583765).

### Quantitative PCR

To determine transgene expression, *Arabidopsis* T2 seedlings were grown on MS 1% sucrose 0.8% agar with phosphinothricin (10 µg/mL concentration) or kanamycin (50 µg/mL concentration), for 21 days in 16 h light at 22 °C. Leaves from each line was treated with DMSO or estradiol as above and harvested 48 h later in liquid nitrogen. Samples were homogenized in a Geno/Grinder® 2010 (SPEX® SamplePrep, USA), with RNA extracted (RNeasy® Plant Mini Kit, QIAGEN) DNase treatment (TURBO DNA-*free*^TM^, Invitrogen/Thermo Fisher), first strand cDNA synthesis (High-Capacity cDNA Reverse Transcription Kit, Thermo Fisher) and qPCR (LightCycler® 480 SYBR® Green I Master, Roche) performed as previously described (Bellandi et al, 2022). Genes tested are *icals3m*/*CalS3* (using primers AGGACGTTACTTCATGGCGG and GGCTCCGGGAATACAGTCTG) and the housekeeper *AtUBQ10* (using primers AGTCTACTCTTCACTTGGTCCTGC and GCCCCAAAACACAAACCACCAAAG). Normalized relative quantities (NRQs) were generated using the qBase model (Hellemans et al, 2007). For the NahG×icals3m cross, the *NahG* transcript was tested (using primers ACTGGAACTCTGCCGCTA and TGAGTTACTAGGGCGTCG; Heck et al, 2003) and the housekeeper *GAPC2 5’* (using primers TCGGAAGAATCGGTCGTTTGG and TGTATGTCATGTACTCGGTGG). The fold change relative to *NahG* was generated by calculating the ∆*C*_*T*_ value [*C*_*T*_(Target) – *C*_*T*_(Housekeeper)] for each sample, then calculating the ∆∆*C*_*T*_ value utilizing the average ∆*C*_*T*_ of NahG [∆*C*_*T*_(sample) − x̄ of ∆*C*_*T*_(NahG)]. The relative fold change in gene expression was calculated (2^−∆∆CT^, Livak and Schmittgen, 2001).

### Pseudomonas syringae infection assays

Three leaves from 4–5-week-old plants were treated with DMSO or estradiol. After 72 h, leaves were infiltrated with 5 × 10^4^ cfu/mL *Pseudomonas syringae* DC3000 and harvested 72 h later. Each leaf was harvested with a 5 mm corer and the three leaves from a single plant combined as a sample. Leaf tissue was homogenized with 1 mL ddH_2_O for 2 min at 1200 rpm in a Geno/Grinder® 2010. 5 µL of multiple 10-fold dilutions were plated out with three technical replicates per plant sample, then incubated for 48 h at 28 °C with colony counts determined from countable dilutions. Four independent experiments with five plants per treatment/genotype per experiment was performed.

To determine plasmodesmal associated callose deposits after *Pseudomonas syringae* DC3000 *hrcC*^*-*^ (*hrcC*^*-*^) infection, a culture at an OD of 0.2 was prepared. Leaves from 5-week-old Col-0 plants were infiltrated either with H_2_O (mock) or *hrcC*^*-*^, and then imaged 24 h, 48 h or 72 h post treatment. Aniline blue staining was performed as per the above plasmodesmal callose measurements with the exception that 0.1% aniline blue was used. 3–4 z-stacks from multiple areas per leaf was performed, with eight biological replicates per treatment/timepoint collected. Only callose deposits at plasmodesmata were analyzed.

### Botrytis cinerea infection assays

*B. cinerea* spores were applied to leaves from 4–5-week-old plants 72 h post DMSO or estradiol treatment. *B. cinerea* spores were harvested and adjusted to a concentration of 2.5 × 10^5^ spores/mL in 0.25× potato dextrose broth and incubated at room temperature for 2 h with continual shaking for spore germination. Leaves were adhered to 1.5% agar H_2_O plates, and droplets of 2 µL spore suspensions were placed in between the mid vein and leaf edge on each side. Plates were sealed with parafilm and incubated in a cabinet set at 22 °C with 10 h light. Developing disease lesions were photographed 2 dpi and measured using FIJI. Each prepared plate had four leaves per genotype and treatment, and the average lesion area was calculated per genotype/treatment in a given plate, defined as a single biological replicate. Three independent experiments were conducted with a minimum of four biological replicates per genotype/treatment in a given experiment.

### MAPK activation assays

MAPK activation assays were performed on leaf discs from expanded leaves of 5-week-old plants or on 15-day-old seedlings 72 h post treatment with DMSO or estradiol. Samples were harvested 0, 5 min, 15 min, 30 min, and 45 min post 100 nM flg22 treatment, and flash frozen in liquid nitrogen. Frozen samples were homogenized via 90 s of shaking (1100 rpm) in a Geno/Grinder® 2010, then 500 µL protein extraction buffer (50 mM Tris-HCl pH 7.5, 150 mM NaCl, 1 mM EDTA pH 8.0, 10% glycerol, 0.5% NP40 IGEPAL® CA-630, protease inhibitor cocktail (cOmplete^TM^ ULTRA Tablets) 1:100, phosphatase inhibitor (PhosSTOP^TM^, Roche/Sigma-Aldrich) 1:200, 1 mM Na_2_MoO_4_×2H_2_O, 1 mM NaF, 1.5 mM activated Na_3_VO_4_, 5 mM DTT, 1 mM PMSF) was added and the sample left to mix for 30 min at 4 °C. Samples were centrifuged at max speed for 10 min at 4 °C, supernatant transferred to a new tube and re-centrifuged at max speed for another 10 min at 4 °C. The resulting supernatant was incubated at 95 °C for 15 min with Laemmli buffer, then proteins were separated by SDS-PAGE and transferred to Immun-Blot® PVDF membrane (Bio-Rad). Proteins were detected using p44/42 MAPK (1:2000; Cell Signaling Technologies, #9102) and α-Rabbit-HRP (1:10,000, Sigma-Aldrich, A0545).

### ROS burst measurements

ROS assays were performed on leaf discs taken from 5-week-old plants or 15-day-old seedlings 72 h post DMSO or estradiol treatment. Leaf discs were first floated on H_2_O overnight; the following day water was removed, then 20 µg/mL HRP and 6 µM L-012 was added, with or without 100 nm flg22. The same was performed for seedlings except liquid media was removed before adding the HRP and L-012. Chemiluminescence was recorded using a Varioskan Flash (Thermo Fisher) over 45 min, with the total luminescence emitted used for analysis.

### General ROS quantification

20 µM H_2_DCFDA (Thermo Fisher) was syringe infiltrated into expanded leaves of 5-week-old plants 72 h post DMSO or estradiol treatment, and confocal microscopy was performed on the abaxial side of the leaf. Z-stacks were taken from four areas of a given leaf from four plants per genotype/treatment. Quantification of fluorescence was performed in FIJI using a sum projection.

### RNA-seq

Two mature leaves per 5-week-old plant were DMSO or estradiol treated, and harvested 12 h, 24 h, 48 h, and 72 h post treatment. Each biological replicate contained four plants (i.e., 8 leaves), with three biological replicates for each genotype/treatment/timepoint. Leaves were flash frozen in liquid nitrogen, then homogenized via 90 s of shaking (1100 rpm) in a Geno/Grinder® 2010. Total RNA was extracted using the RNeasy® Plant Mini Kit (QIAGEN) and eluted into 60 µl of water. Purified RNA was treated with the TURBO DNA-*free*^TM^ kit (Invitrogen/Thermo Fisher). RNA quantification and library construction was conducted by Novogene Co., Ltd (Beijing). RNA quality and quantity were access via 1% agarose gel, NanoPhotometer ® spectrophotometer (IMPLEN, USA), and the RNA Nano 6000 Assay Kit for the Bioanalyzer 2100 System (Agilent Technologies). Libraries were constructed using 0.4 µg of RNA and the NEBNext ® UltraTM RNA Library Prep Kity for Illumina (NEB). mRNA was purified via poly-T magnetic beads. Fragmentation, cDNA synthesis, and NEBNext Adaptor hybridization were performed according to manufacturer’s instructions and purified with the AMPure XP system (Beckman Coulter). Index-coded samples were clustered on a cBot Cluster Generation System using TruSeq PE Cluster Kit v3-cBot-HS (Illumina) and sequenced on an Illumina Novaseq platform to generate 150 bp paired-end reads with a sequencing depth of 10 million reads per sample. All sequencing data are available on ArrayExpress at E-MTAB-16776.

### Bioinformatic analyses

Adaptors and low-quality (phred33 < 20) reads were trimmed from the data using TrimGalore v0.5.0 (Krueger et al, 2021; 10.5281/ZENODO.5127899) and Cutadapt v1.7 (Martin, 2011). RNA quality was accessed using FastQC v0.11.8 (Babraham Bioinformatics, 2023) and MultiQC v.1.7 (Ewels et al, 2016). The data was mapped against the *Arabidopsis* reference genome (TAIR10; NCBI Refseq GCA_000001735.1) using HISAT2 v2.1.0 (Kim et al, 2019). Mapped reads were sorted using Samtools v1.4.1 Sort (Danecek et al, 2021). Transcript assembly was completed using Stringtie v1.3.3 (Pertea et al, 2015) and gene counts (see Dataset EV10 and Dataset EV11 for gene count matrix and metadata, respectively) were extracted using the built-in python wrapper, prepDE.py available at http://ccb.jhu.edu/software/stringtie/dl/prepDE.py. Principle component analysis was conducted using DEseq2, ggplot2 (Wickham, 2016), ggConvexHull, and TidyVerse (Wickham et al, 2019) in R (R Core Team, 2022). Differentially expressed genes were determined using DEseq2 (Love et al, 2014) in R. Genes with less than 10 counts across all samples were excluded from the analysis. The data was subset by timepoints, and Wald tests or Likelihood ratio tests (LRT) were performed based on comparisons of interest. Wald Tests were used to extract DEGs based on differences between treatment or genotype. LRT were used to extract genes differentially expressed based on difference between closed plasmodesmata (induced/estradiol treated LexA::PD-Plug and LexA::icals3m combined) and open plasmodesmata (uninduced/DMSO treated LexA::PD-Plug, uninduced/DMSO treated LexA::icals3m, and DMSO and estradiol treated Col-0). LRT was used in this way to determine genes uniquely up- or down-regulated in the inducible lines treated with estradiol. Differentially expressed genes from the Wald Test were designated if the log2 fold change (lfc) was greater than |1.5| and adjusted *p*-value < 0.05. Differentially expressed genes from the LRT were considered significant if the adjusted *p*-value was less than 0.05. LRT significantly differentially expressed genes were clustered using Lasso2 (Lokhorst et al, 2021) and DEGreports v1.36.0 (Pantano, 2023) and normalized read counts plotted using ggplot2 and pheatmaps v1.0.12 (Kolde, 2019). Genes involved in Salicylic Acid biosynthesis/catabolism and response were determined based on a Wald Test of open plasmodesmata versus closed plasmodesmata with a lfc cut-off of +/−0.5 and adjusted *p*-value of 0.05 (Dataset EV7). Gene ontology enrichment analysis was conducted based on differentially expressed genes. Enrichment was conducted in TopGo v2.52.0 (Alexa and Rahnenführer, 2023) based on TAIR10 GO terms, with a Fisher’s exact test cut-off of *p* < 0.05. GO terms were plotted in ggplot2. GO parent terms were determined using the Revigo’s (Supek et al, 2011) redundancy feature (cut-off 0.7) and utilized to extract defense-specific enriched GO terms.

### Transgene mapping

Transgenes were mapped by generating a pseudo-genome consisting of the *Arabidopsis* TAIR genome as well as the transgene of interest (mCherry) using SeqKit v0.9.1 (Shen et al, 2016) and STAR v2.5a (Dobin et al, 2013). The reads were then mapped and sorted by coordinates to the pseudo-genome using STAR v2.5a. The data was processed the same as previously described using Samtools and Stringtie. Gene counts of mCherry (Dataset EV12) and CalS3 (Dataset EV13) were extracted using DESeq2 and plotted in ggplot2.

### Size phenotyping

9-day-old seedlings of Col-0, LexA::PD-Plug and LexA::icals3m were finely sprayed with treatments DMSO or estradiol and 6 days later, the first two true leaves were adhered to a microscope slide and images taken on a Leica M205 FA stereo microscope. Leaf area was measured using FIJI. For the shade experiments, 10-day-old seedlings of Col-0, LexA::icals3m, NahG and NahG×icals3m were finely sprayed with treatments of DMSO or estradiol and either kept at normal light conditions (80 μmol/m^−2^/s^−1^) or a shaded lid was placed on top reducing the light intensity to 9 μmol/m^−2^/s^−1^. An additional spray was performed 7 days post the initial treatment, and 14 days post treatment, the first two true leaves were adhered to a microscope slide and images taken on a Axio Zoom V16 light microscope. A leaf measurement macro developed using FIJI was used to measure the leaf area (Tee et al, 2026; 10.5281/zenodo.18887793); the average between the two true leaves per plant was calculated, and the relative size was calculated by dividing the average of the DMSO control in each condition/genotype for each sample.

### Sugar and starch measurements

Leaf tissue was harvested in liquid nitrogen from plants 72 h post treatment of either DMSO or estradiol (3 leaves from 5-week-old plants), with sugars and starch separately extracted from approximately 100 mg total leaf tissue as described by Smith and Zeeman (2006). Frozen tissue was homogenized for 2 min at 1200 rpm in a Geno/Grinder® 2010. Samples were further homogenized in 1 mL of 0.7 M cold perchloric acid and then centrifuge at max speed 4 °C for 5 min. The supernatant was transferred to process for sugar, while the pellet was kept on ice for starch quantification. For sugar, 600 µL supernatant was transferred to a new 1.5 mL tube, and neutralization buffer (2 M KOH, 400 mM MES) was added to achieve a pH of 6–7. The sample was then spun at max speed for 4 min and supernatant recovered. Soluble sugars were quantified by enzymatic assay in technical triplicates by monitoring NADH production absorbance at 340 nm in a 96-well format using an Omega FLUOstar microplate reader. Each sample was added to 50 µL of buffer (50 mM HEPES NaOH pH 7.4–7.6, 1 mM MgCl_2_, 1 mM ATP, 1 mM NAD, 1.4 U Hexokinase), topped with H_2_O to a total volume of 198 µL. An initial A_340_ reading was taken, before adding glucose-6-phosphate dehydrogenase for a kinetic reading to determine glucose content, followed by adding phosphoglucose isomerase to determine fructose content, followed by invertase to determine sucrose content. To determine starch content, the sample pellet was first washed by adding 1 mL H_2_O and vortexing, followed by centrifugation for 3 min at 3000 × *g*. Supernatant was removed, then pellet washed again by adding 1 mL 80% ethanol and vortexing, followed by centrifugation for 3 min at 3000 × *g*; this ethanol wash was repeated for a total of three washes. Excess ethanol was evaporated, then resuspended with 750 µL of H_2_O. 2 × 200 µL of each sample was transferred to a new screw cap tube and incubated at 95 °C for 12 min. The samples were left to briefly cool to room temperature (~5 min), then one aliquot was digested by incubation with α-amylase (10 U) and amyloglucosidase (1.26 U) in 0.1045 M sodium acetate buffer (pH 4.8) for two hours at 37 °C, alongside the other aliquot serving as the non-digested control (i.e., containing only the 0.1045 M sodium acetate buffer and two hour 37 °C incubation). Starch content was quantified through enzymatic assay of the digested aliquot sample by monitoring NADH production as per the above glucose measurement. Technical triplicates were performed for each digested sample, alongside a single technical replicate for the non-digested sample. Starch content was calculated from the glucose assay values in mg per gram of fresh weight of sample.

### Hormone measurements

Leaves from 5-week-old plants were treated with DMSO or estradiol and harvested 72 h post treatment and flash frozen in liquid nitrogen; per biological replicate, a minimum of six leaves from a minimum of three plants were pooled together to obtain between 150–220 mg of fresh tissue weight. Frozen tissue was homogenized for 2 × 2 min at 1200 rpm in a Geno/Grinder® 2010. 300 µL of buffer (10% methanol, 1% acetic acid in H_2_O) containing an internal standard of 1 µM salicylic acid-d_4_ (SA-_d4_, Merck) and 500 nM jasmonic acid-d_5_ (JA-_d5_, Cayman Chemicals) was added, mixed well with a vortex then left to mix at 4 °C for 20 min. Samples were centrifuged for 25 min at 4 °C at 15,000 RCF, and the supernatant recovered. The pellet was re-extracted with another 300 µL buffer without the internal standard, mixed and centrifuged as before, with the resulting supernatant combined with the first recovered supernatant. The total supernatant was centrifuged at 4 °C for 10 min at max speed, and each sample was then run for analysis. Quantification was performed on an Acquity UPLC attached to a Xevo TQS tandem mass spectrometer (Waters). Separation was on a 50 × 2.1 mm 2.6 µM Kinetex EVO C18 column (Phenomenex) using the following gradient of acetonitrile (solvent B) versus 0.1% formic acid in water (solvent A), run at 600 µL.min^−1^ and 35 °C: 0 min, 10% B; 2 min, 80% B; 2.15 min, 80% B; 2.2 min, 10% B; 3.4 min, 10% B. The injection volume was 5 µL. Salicylate was quantified by negative mode electrospray, monitoring the transition *m/z* 137 → 65 (and for the internal standard the corresponding transition 141 → 97) while jasmonate was monitored at the transition *mz* 209 → 59 (and for the internal standard the corresponding transition 214 → 62) at 16 V collision energy. Spray chamber conditions were 1.5 kV spray voltage, 500 °C desolvation temperature, 900 L.h^−1^ desolvation gas, 150 L.h^−1^ cone gas, and 7 bar nebulizer pressure. Results were processed using TargetLynx software V4.1 SCN876 (2012).

### Chlorophyll fluorescence measurements

Chlorophyll fluorescence measurements were performed using an Imaging-PAM chlorophyll fluorometer and ImagingWin software application (Walz). The effective PSII quantum yield as indicated by Y(II) was taken following a 30 min dark adaptation of leaves from 5-week-old plants 72 h post DMSO or estradiol treatment. Four leaves per treatment/genotype were measured in a given run, with 8 runs in total over two independent experiments.

### Statistical analyses

Statistical analyses were performed using RStudio 2021.09.01 Build 351/R version 4.0.3. Data presented as boxplots have the middle line indicating the median, the box representing the upper and lower quartiles, and the whiskers indicating the minimum and maximum values with 1.5× interquartile range. For bootstrap analyses, data were analyzed using *medianBootstrap* (Johnston and Faulkner, 2021) or *medianBootstrap2* (Tee et al, 2023a; 10.5281/zenodo.7680790). Pathoassays, basal ROS quantification, and chlorophyll fluorescence measurements were analyzed using a linear-mixed effects model using the R package, *lmerTest*. For pathoassays, the random effect was ‘independent experiment repeat’; for the basal ROS quantification the random effect was ‘biological replicate’; for chlorophyll fluorescence measurements the random effect was ‘experimental run’. Size phenotyping, starch, sucrose, glucose and fructose measurements were analyzed using a linear model. All ANOVAs specified are ANOVA Satterthwaite’s Method, and significant differences between factors were determined by post hoc Tukey HSD using the R package, *emmeans*. Salicylic acid quantification was analyzed using a Mann–Whitney test.

## Supplementary information

Appendix

Peer Review File

Dataset EV1

Dataset EV2

Dataset EV3

Dataset EV4

Dataset EV5

Dataset EV6

Dataset EV7

Dataset EV8

Dataset EV9

Dataset EV10

Dataset EV11

Dataset EV12

Dataset EV13

Source data Fig. 1

Source data Fig. 2

Source data Fig. 3

Source data Fig. 4

Expanded View Figures Source Data

Expanded View Figures

## Data Availability

The original images for callose staining for Fig. 1A, Fig. EV1 and Fig. EV3 and bombardment images for Fig. 1B and Fig. 1C are available at BioStudies, accessible with the accession number of S-BIAD3028. RNAseq data has been deposited at ArrayExpress BioStudies database under the accession code E-MTAB-16776, with further analysis included in Datasets as specified in-text. The source data of this paper are collected in the following database record: biostudies:S-SCDT-10_1038-S44319-026-00789-2.
