# Supplementary material for: Plasmodesmal closure elicits stress responses
Source: EMBO Rep. 2026 May 2;27(12):3231–51. doi: 10.1038/s44319-026-00789-2 (PMC13303860; doi:10.1038/s44319-026-00789-2)
Supplement: Supplementary file 1 — Appendix [file 44319_2026_789_MOESM1_ESM.pdf]

Plasmodesmal closure elicits stress responses

Estee E. Tee, Andrew Breakspear, Diana Papp, Hannah R. Thomas, Catherine Walker, Annalisa Bellandi, & Christine Faulkner

Table of Contents

Appendix Figure S1 – page 2
Appendix Figure S2 – page 3
Appendix Figure S3 – page 4
Appendix Figure S4 – page 5
Appendix Figure S5 – page 6
Appendix Figure S6 – page 7
Appendix Figure S7 – page 8
Appendix Figure S8 – page 9
Appendix Figure S9 – page 11
Appendix Figure S10 – page 12
Appendix Figure S11 – page 13
Appendix Figure S12 – page 14
Appendix Figure S13 – page 15
Appendix Figure S14 – page 16
Appendix Figure S15 – page 17
Appendix Figure S16 – page 18
Appendix Figure S17 – page 19

Appendix Table S1 – page 20
Appendix Table S2 – page 21

Appendix References – page 22

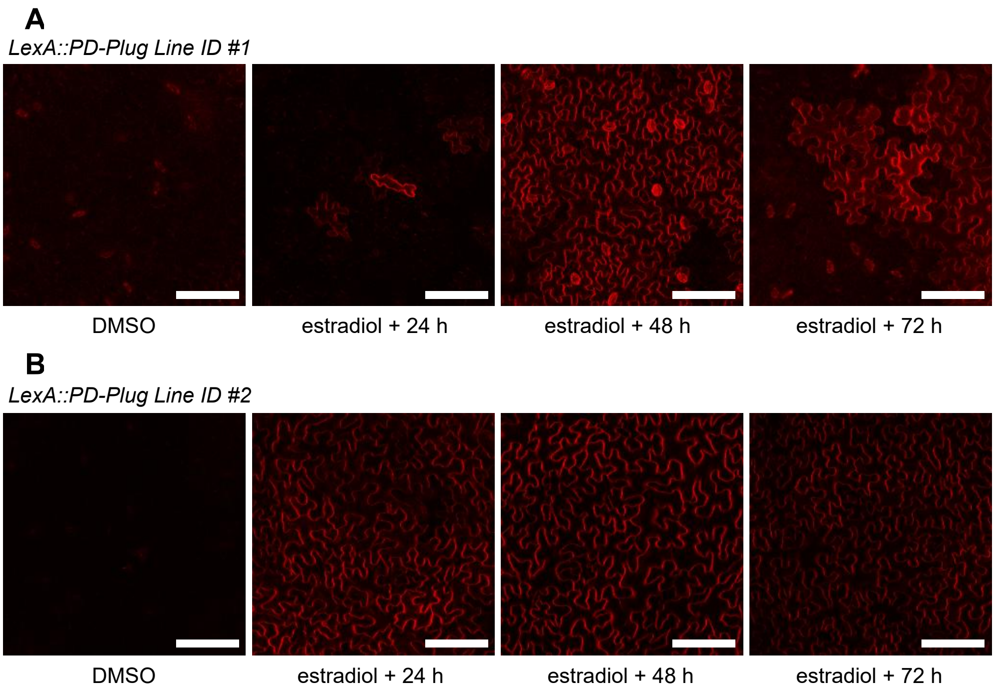

Appendix Figure S1. Induction of LexA::PD-Plug transgene construct after estradiol treatment in T2 independent lines. Independent T2 lines of LexA::PD-Plug with mCherry expression showing induction post estradiol treatment. Line 2 was selected for further experimental analysis. Scale bar = 100μM.

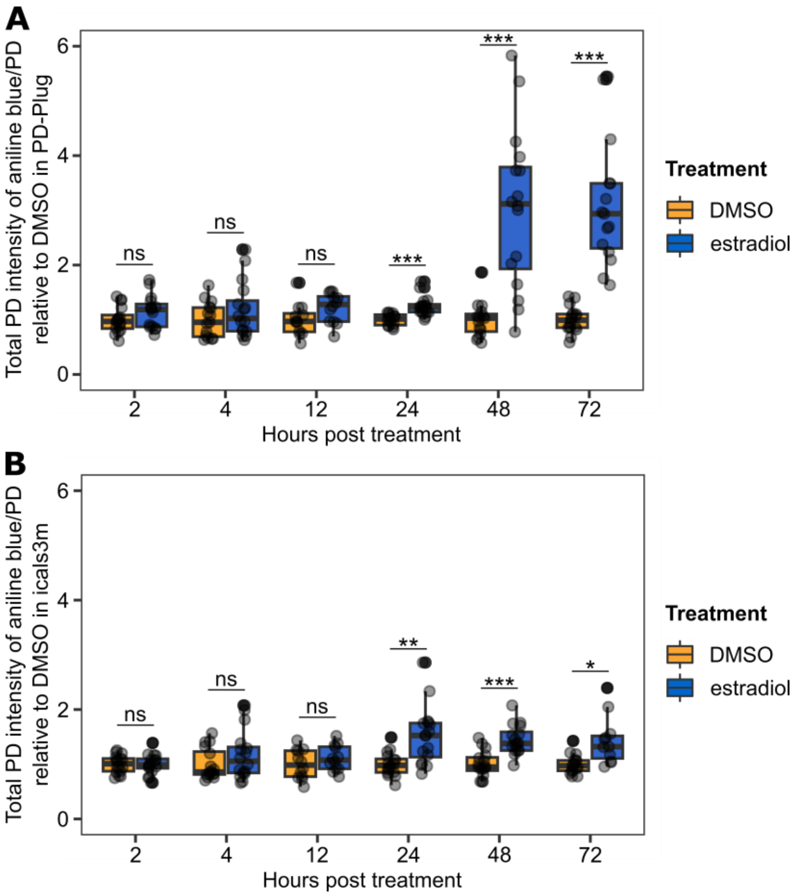

Appendix Figure S2. Time course callose quantification in LexA::PD-Plug and LexA::icals3m treated with DMSO or estradiol. Aniline blue-stained callose deposition at plasmodesmata quantification in either LexA::PD-Plug (**A**) or LexA::icals3m (**B**). Bootstrap analysis indicates significant differences between DMSO and estradiol treatment at each time point, as indicated by * p<0.05, ** p<0.01, or *** p<0.001, with n ≥ 11 images with a minimum of 3 biological replicates per genotype/treatment/timepoint. For all plots, the center line marks the median, the box indicates the upper and lower quartiles, and the whiskers show the minimum and maximum values within 1.5× interquartile range.

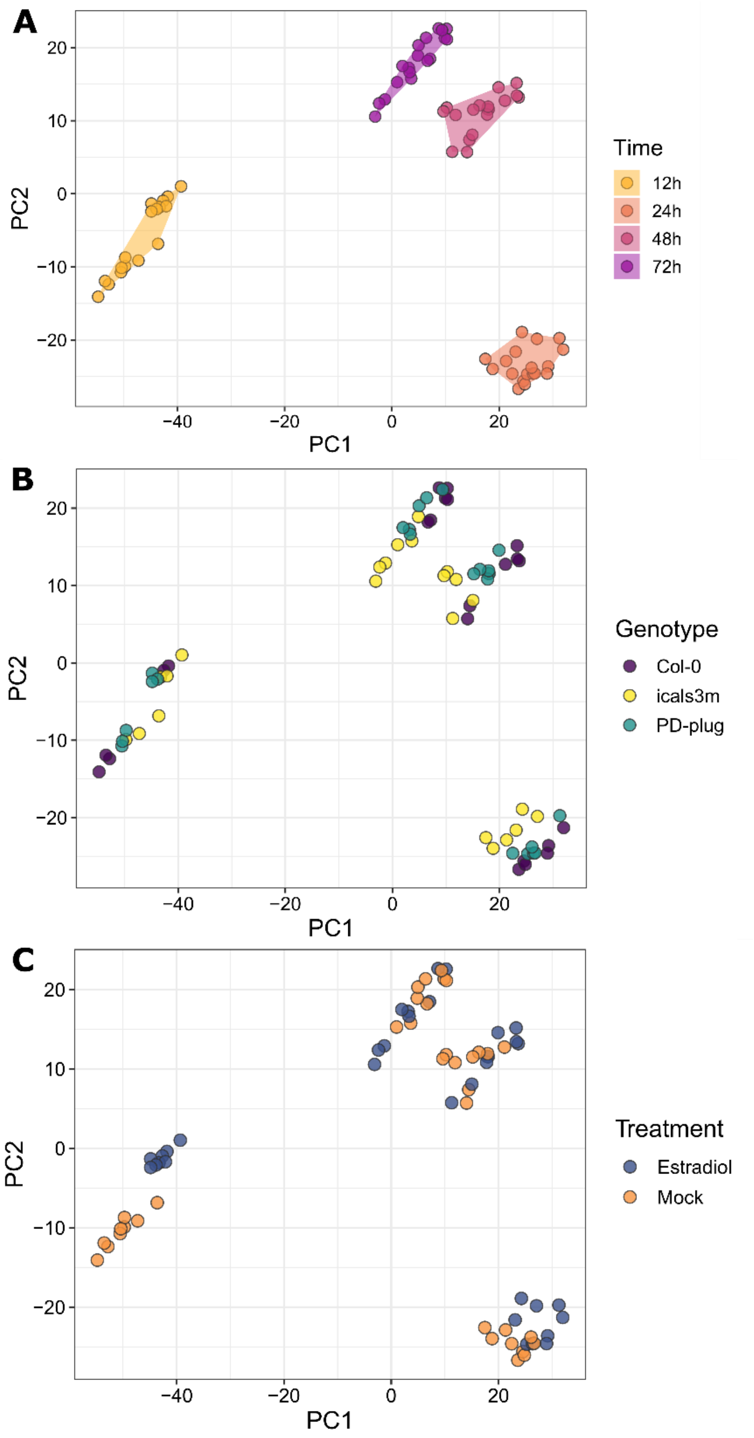

Appendix Figure S3. PCA of genotypes Col-0, LexA::PD-Plug and LexA::icals3m treated with DMSO or estradiol at each time point. PCA grouped based on time (**A**), genotype (**B**) and treatment (**C**) indicate a critical driving factor of variance was time.

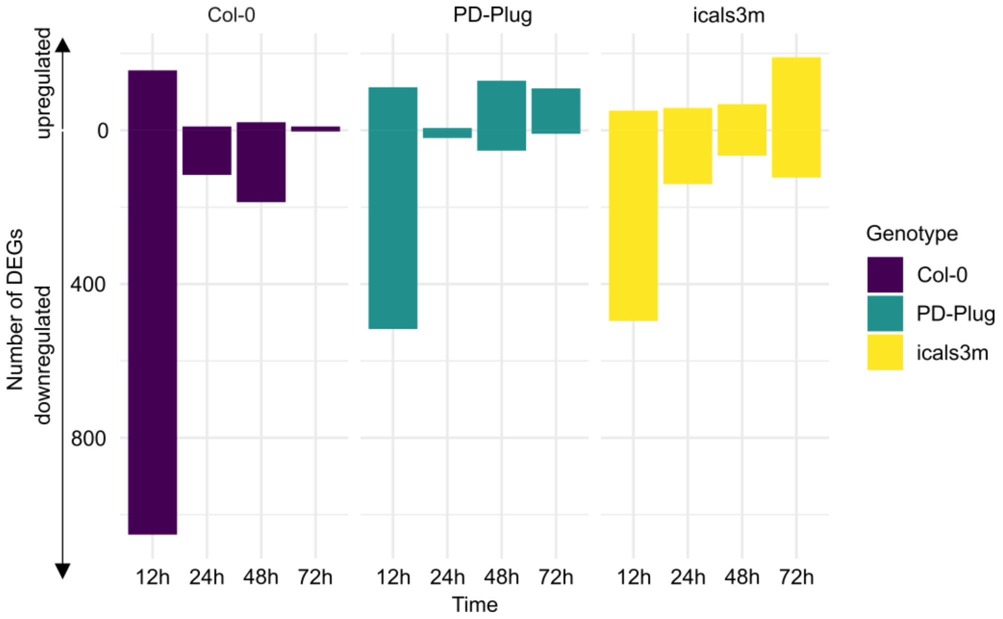

Appendix Figure S4. Estradiol induces transcriptional changes. Number of differentially expressed genes (DEGs) in each genotype, when treated with estradiol compared to DMSO. 12 h after treatment, hundreds of genes are downregulated in all genotypes with a large effect seen in Col-0. The effect of estradiol in Col-0 is largely negated by 24 h (see Dataset EV1 for shared genes across genotypes), with no effect of estradiol detected in Col-0 by 72 h.

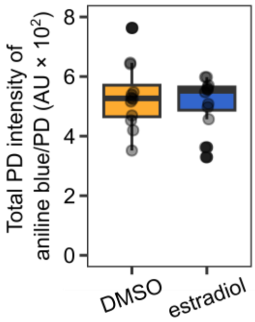

Appendix Figure S5. Estradiol has no effect on plasmodesmal callose accumulation Col-0 at 12 h. Aniline blue-stained callose deposition at plasmodesmata quantification in Col-0 with either DMSO or estradiol treatment. Bootstrap analysis indicates no significant difference between treatments, with n = 12 images and 3 biological replicates per treatment. The center line marks the median, the box indicates the upper and lower quartiles, and the whiskers show the minimum and maximum values within 1.5× interquartile range.

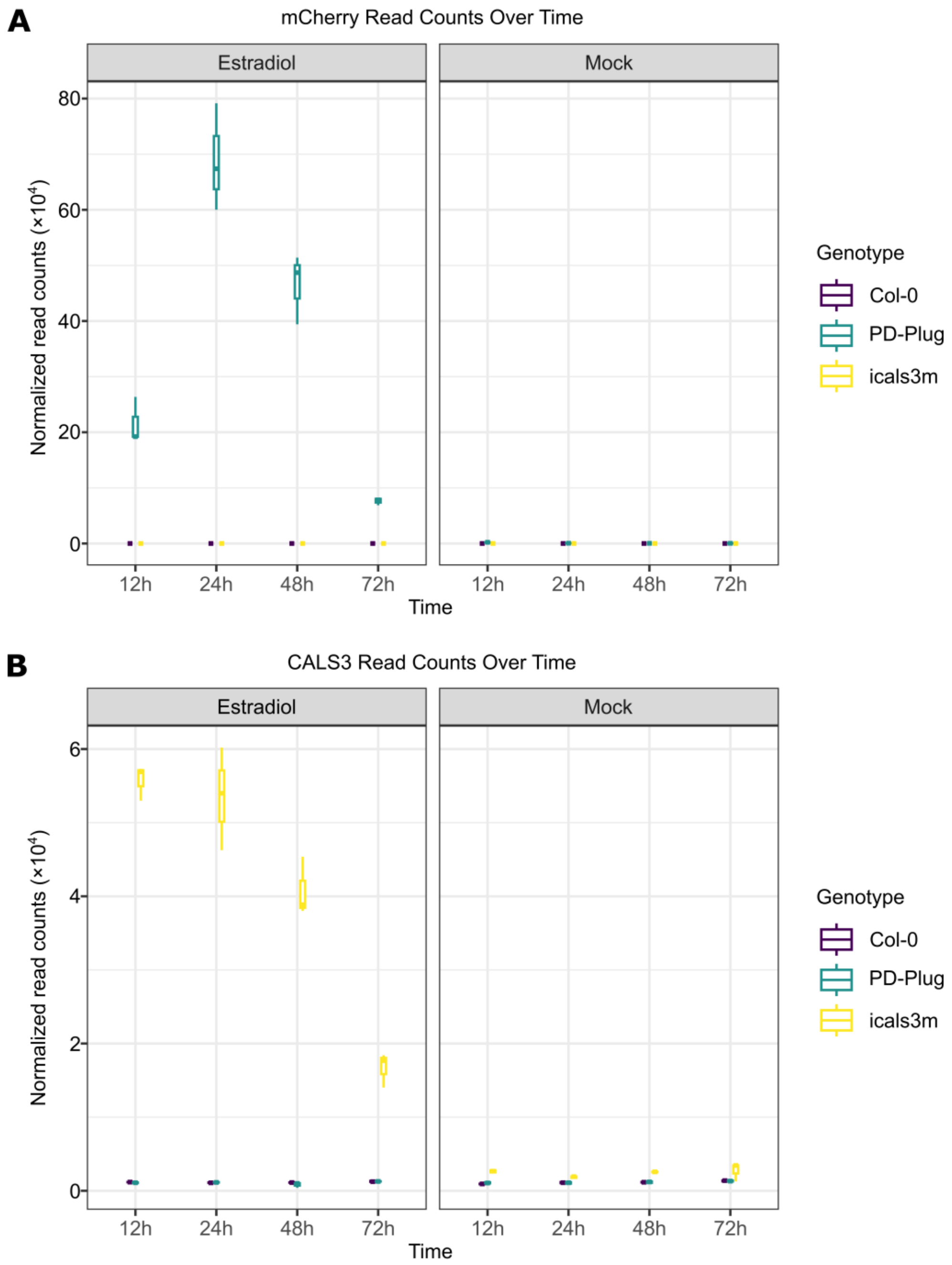

Appendix Figure S6. Estradiol-induced transgene expression over 72 h. Normalized read counts of estradiol induced transgene expression at each time point for each genotype (Estradiol, left panels), in comparison to the respective DMSO treated (Mock, right panels) samples. (**A**) mCherry read counts, indicative of the LexA::PD-Plug transgene (Dataset EV12) (**B**) CalS3 read counts, including reads from both the native CalS3 expression and that induced by the LexA::icals3m transgene (Dataset EV13). For all plots, the center line marks the median, the box indicates the upper and lower quartiles, and the whiskers show the minimum and maximum values within 1.5× interquartile range.

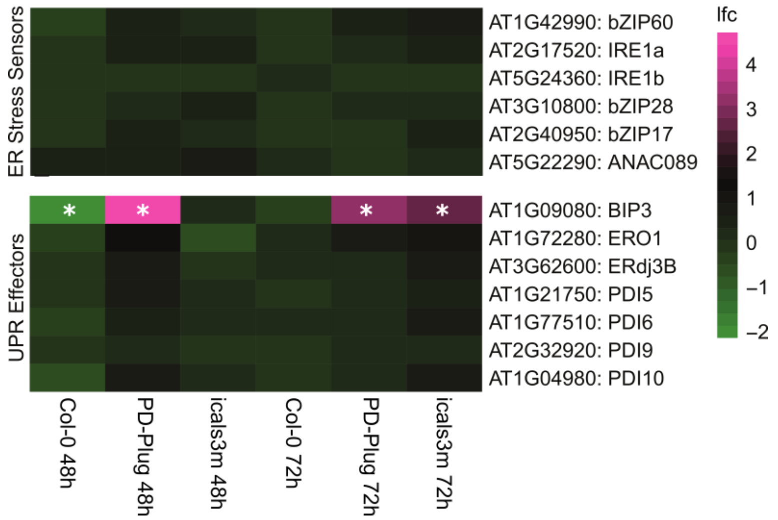

Appendix Figure S7. Gene expression of genes related to ER stress. Effect of estradiol on genes defined as ER stress sensors or unfold protein response (UPR) effectors (Beaugelin et al. 2020), in genotypes Col-0, LexA::PD-Plug and LexA::icals3m. Stars indicate significant up or down-regulation in comparison to DMSO treatment.

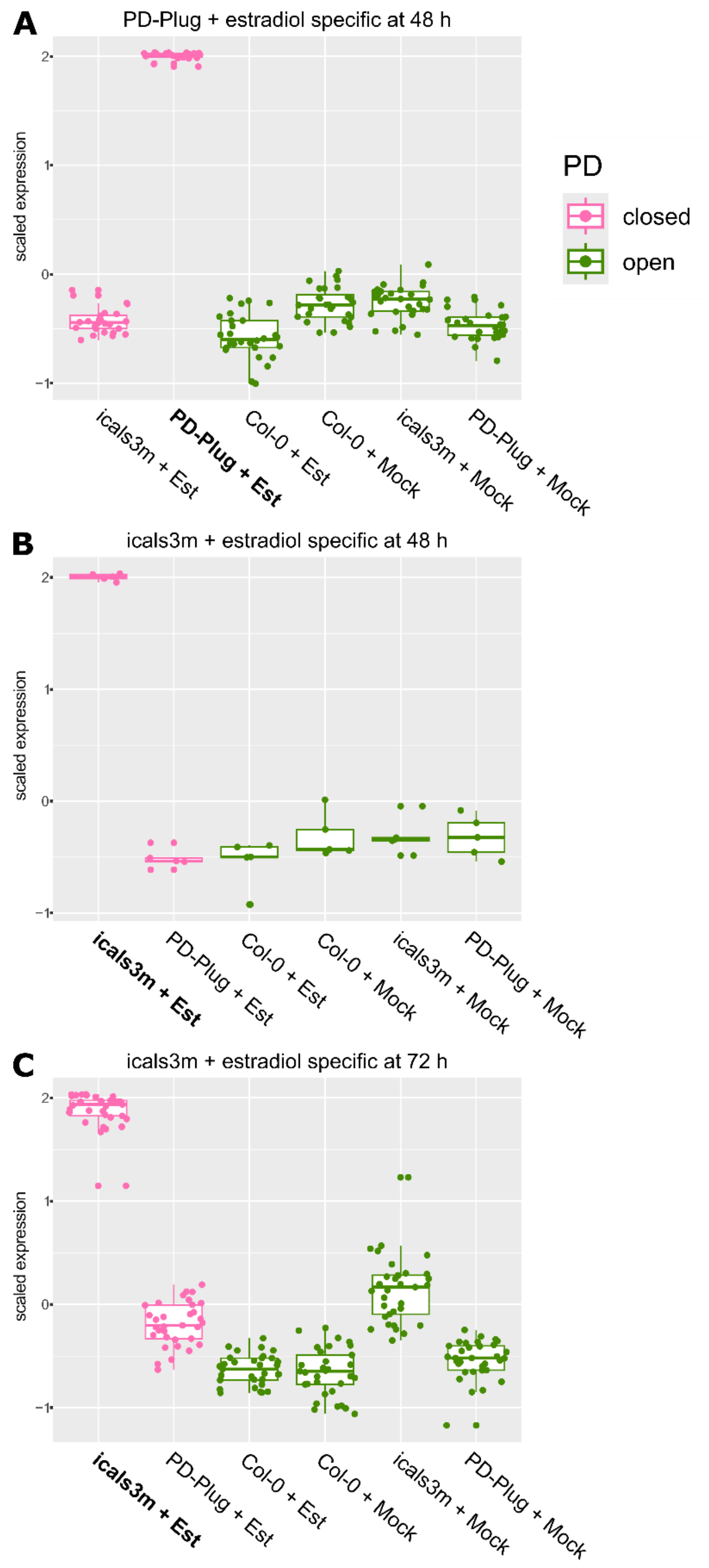

Appendix Figure S8. Likelihood ratio clustering for genes significantly different in estradiol treated LexA::PD-Plug at 48 h (A), estradiol treated LexA::icals3m at 48 h (B) and estradiol treated LexA::icals3m at 72 h (C). Plasmodesmal state (PD) indicated by color, with 'closed' plasmodesmata being LexA::PD-Plug (PD-Plug) and LexA::icals3m (icals3m) treated with estradiol (Est), and 'open' plasmodesmata being LexA::PD-Plug and LexA::icals3m treated with DMSO (Mock), and Col-0 treated with

DMSO (Mock) and estradiol (Est). See Dataset EV2 for gene list. For all plots, the center line marks the median, the box indicates the upper and lower quartiles, and the whiskers show the minimum and maximum values within 1.5× interquartile range.

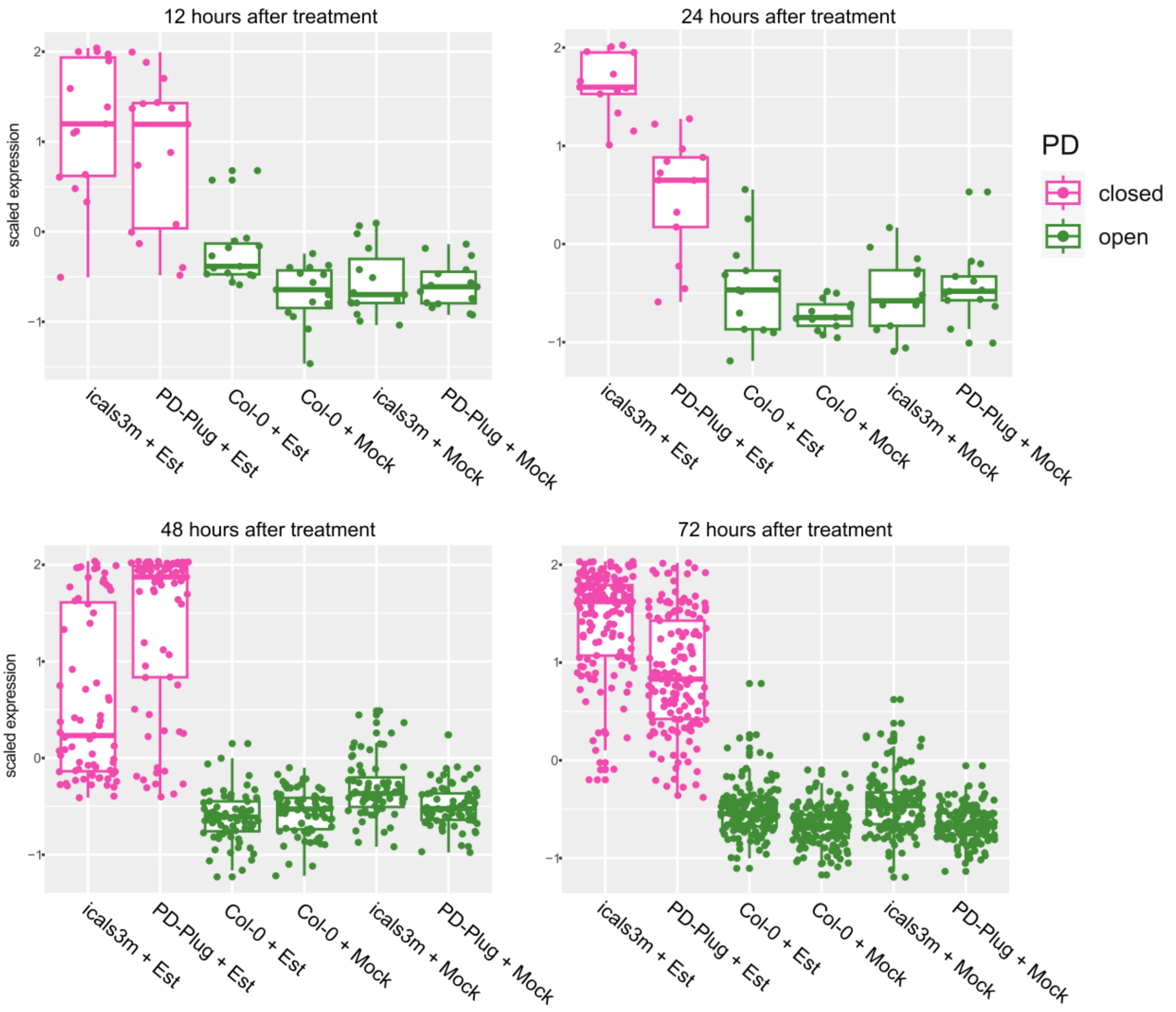

Appendix Figure S9. Likelihood ratio clustering of groups based on plasmodesmal state. Groups based on plasmodesmal state (PD), with ‘closed’ plasmodesmata being LexA::PD-Plug (PD-Plug) and LexA::icals3m (icals3m) treated with estradiol (Est), and ‘open’ plasmodesmata being LexA::PD-Plug and LexA::icals3m treated with DMSO (Mock), and Col-0 treated with DMSO (Mock) and estradiol (Est). See Datasets EV3-6 for gene lists. For all plots, the center line marks the median, the box indicates the upper and lower quartiles, and the whiskers show the minimum and maximum values within 1.5× interquartile range.

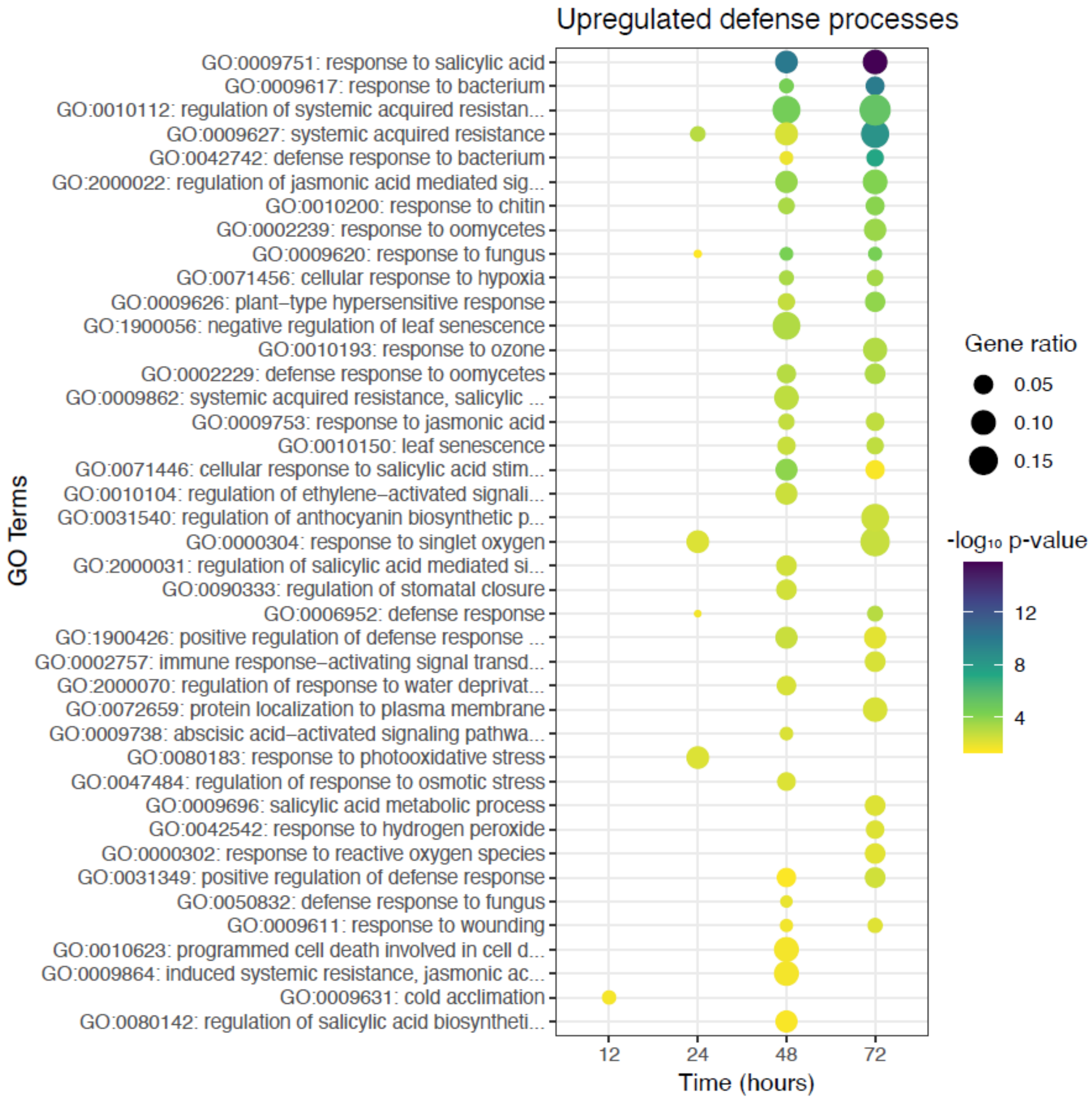

Appendix Figure S10. GO terms enriched when plasmodesmata are closed, under the parent term of defense processes shown across all time points.

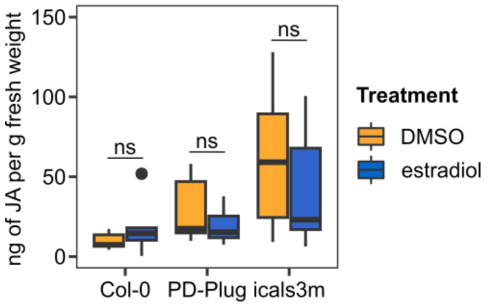

Appendix Figure S11. Jasmonic acid quantification in Col-0, PD-Plug, and icals3m. Quantification of jasmonic acid in 5-week-old plants after 72 h DMSO or estradiol treatment, n = 6 per genotype/treatment. No significant differences (ns) between treatment within a genotype was found using the Mann-Whitney test. The center line marks the median, the box indicates the upper and lower quartiles, and the whiskers show the minimum and maximum values within 1.5× interquartile range.

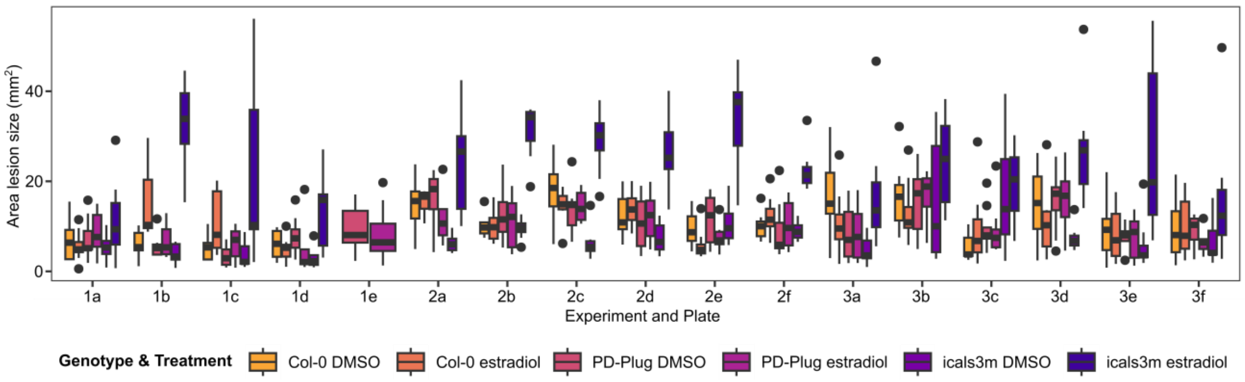

Appendix Figure S12. *Botrytis cinerea* disease lesions in Col-0, LexA::PD-Plug and LexA::icals3m. Area of disease lesions 2 dpi in leaves of 5-week-old plants, inoculated after 72 h of DMSO or estradiol treatment. Graph of each plate within an experiment as indicated by the number (experiment) and letter (plate). Four leaves were represented for each treatment/genotype combination per plate. The center line marks the median, the box indicates the upper and lower quartiles, and the whiskers show the minimum and maximum values within 1.5× interquartile range.

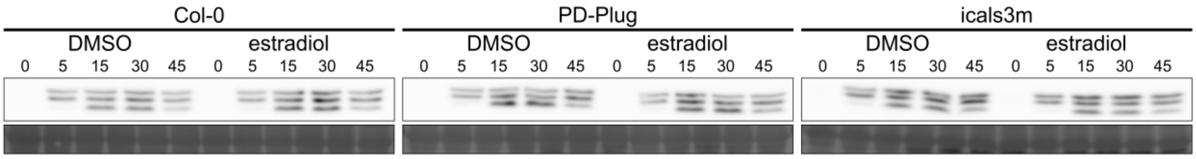

Appendix Figure S13. MAPK-activation by flg22 in seedlings of Col-0, LexA::PD-Plug and LexA::icals3m, 72 h post DMSO or estradiol treatment. Western blot with minute timings denoted, and Coomassie blue staining indicated below as loading controls.

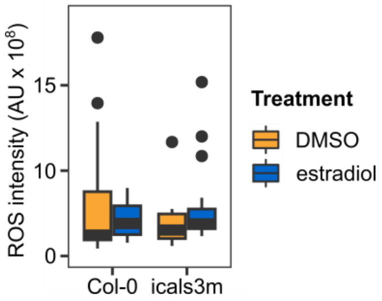

Appendix Figure S14. Basal ROS quantification of LexA::icals3m in comparison to Col-0. Quantification of ROS from z-stacks using sum projection, as indicated utilizing 20 µM H_2DCFDA. 5-week-old plants were treated with DMSO or estradiol for 72 h before visualization, with data from four images per plant, n = 4 per treatment/genotype. Independent factors genotype and treatment were not significant, with no significant interaction between the two (ANOVA; F = 0.05, df = 2, p = 0.83; F = 0.002, df = 1, p = 0.97; F = 1.08, df = 1, p = 0.32). The center line marks the median, the box indicates the upper and lower quartiles, and the whiskers show the minimum and maximum values within 1.5× interquartile range.

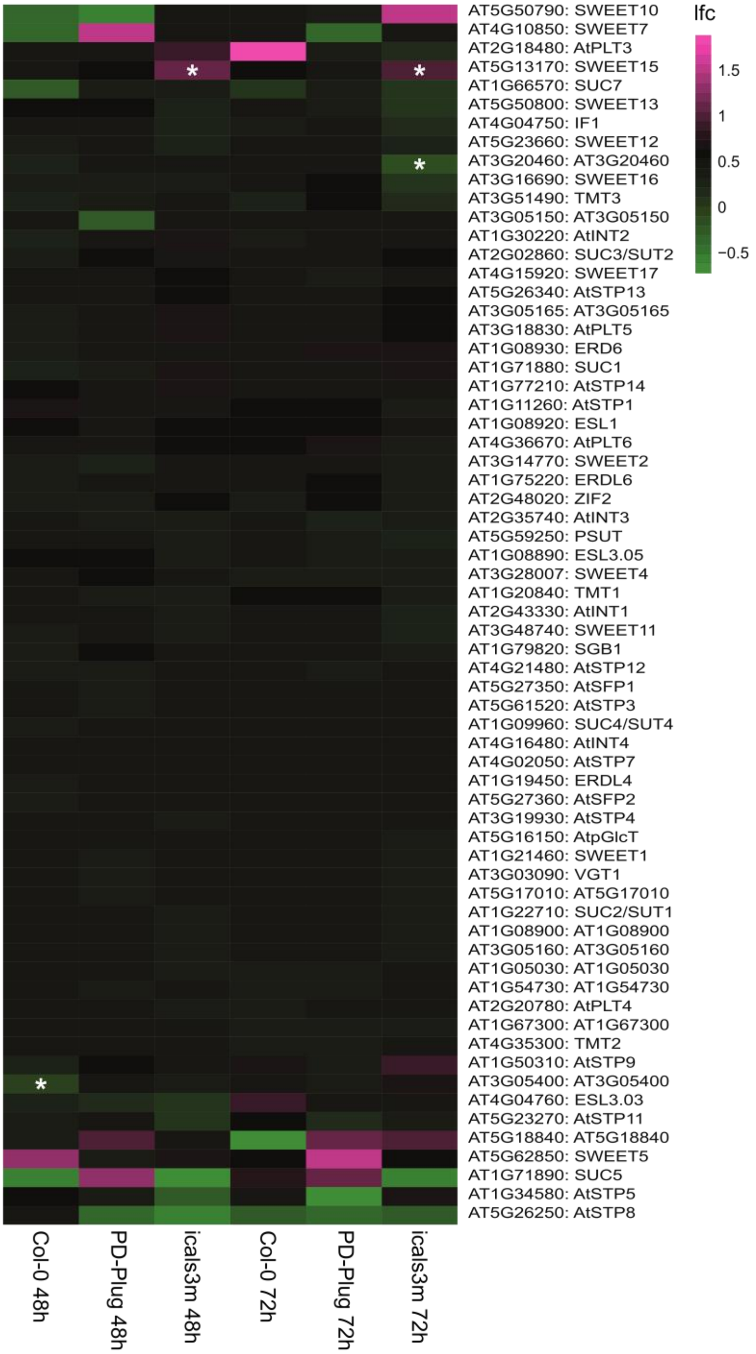

Appendix Figure S15. Heat map showing gene expression of sugar related genes at 48 h and 72 h post treatment. Effect of estradiol on genes related to sugar transporters (Dataset EV9), in genotypes Col-0, LexA::PD-Plug and LexA::icals3m, with stars indicating significant up or down-regulation in comparison to DMSO treatment.

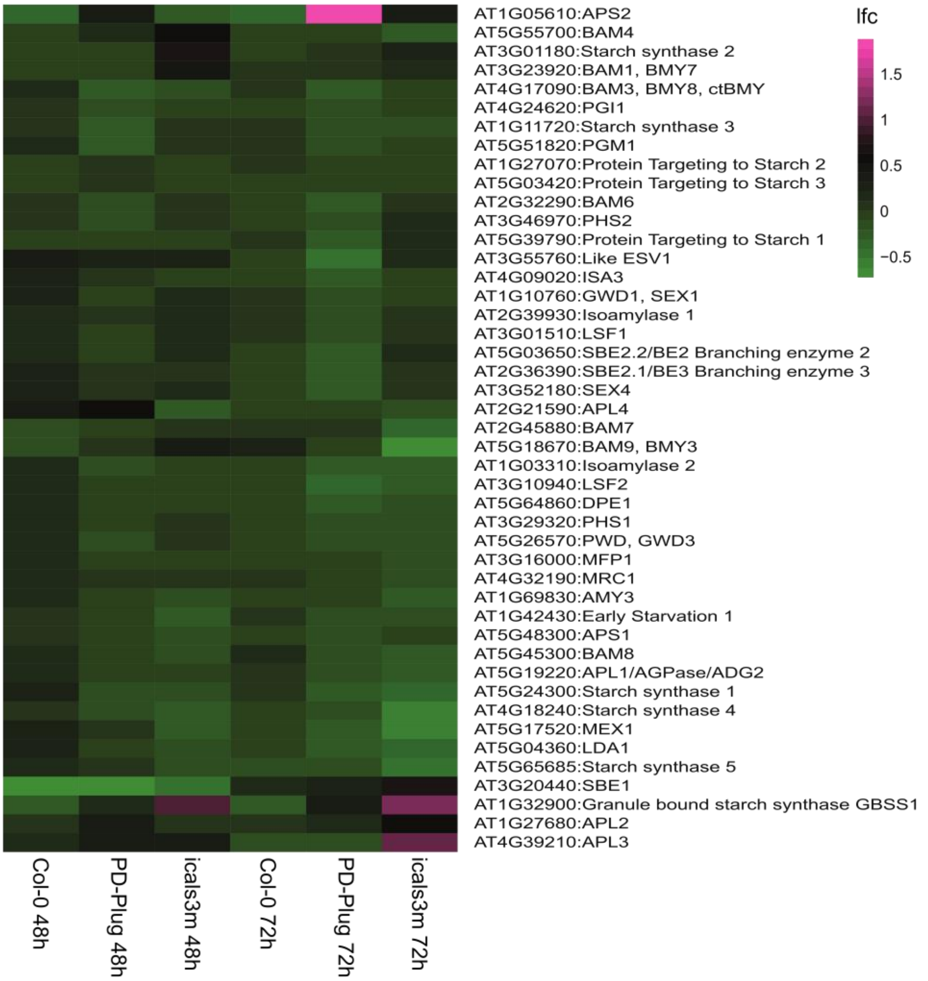

Appendix Figure S16. Gene expression of starch related genes at 48 h and 72 h post treatment. Effect of estradiol on expression of genes related to starch (Dataset EV9) in genotypes Col-0, LexA::PD-Plug and LexA::icals3m.

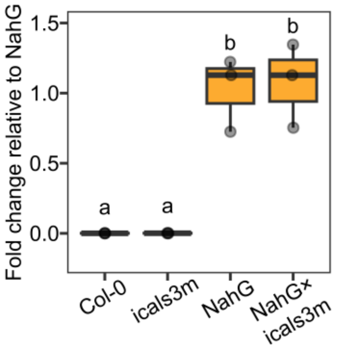

Appendix Figure S17. Relative gene expression of *NahG* in Col-0, icals3m, NahG and NahG×icals3m. N = 3 per genotype. Independent factor genotype was significant (ANOVA; F = 27.6, df = 3, $p < 0.001$). Significant differences denoted by a and b: Col-0 vs icals3m p = 1; Col-0 vs NahG p = 0.001; Col-0 vs NahG×icals3m $p < 0.001$; icals3m vs NahG p = 0.001; icals3m vs NahG×icals3m $p < 0.001$; NahG vs NahG×icals3m p = 0.989. The center line marks the median, the box indicates the upper and lower quartiles, and the whiskers show the minimum and maximum values within 1.5× interquartile range.

Appendix Table S1. Selection information for LexA::icals3m transgenic line. Independent T2 lines as indicated by Line ID, were analyzed for copy number and a qPCR was performed on seedings 24 h post treatment of DMSO or estradiol, with gene expression indicated by relative normalized quantity (NRQ). Fold change difference is estradiol NRQ/DMSO NRQ. Chosen line for characterization indicated by bold and italicized text.

Line ID	Copy Number	DMSO NRQ	Estradiol NRQ	Fold Change
#23	***2***	***0.03***	***0.44***	***15.14***
#10	2	0.02	0.10	6.05
#22	2	0.02	0.05	3.19
#7	3	0.03	0.33	10.73
#19	3	0.07	0.15	2.09
#2	4	0.02	0.31	19.52
#17	4	0.06	0.80	13.93
#6	16	0.07	2.45	34.94
#12	24	0.16	1.19	7.26

Appendix Table S2. Genes identified from the literature as associated with salicylic acid biosynthesis, catabolism and response.

	Gene ID	Name	Reference
Biosynthesis and Catabolism	AT1G73805	SARD1	Zhang et al. 2010
	AT1G74710	ICS1	Wildermuth et al. 2001
	AT3G11480	BSMT1	Chen et al. 2003
	AT3G48090	EDS1	Cui et al. 2017
	AT3G52430	PAD4	Cui et al. 2017
	AT4G10500	DL01	Zhang et al. 2013
	AT4G37150	MES9	Vlot et al. 2008
	AT4G39030	EDS5	Serrano et al. 2013
	AT5G13320	PBS3	Rekhter et al. 2019; Torrens-Spence et al. 2019
	AT5G24530	DMR6	Zhang et al. 2017
	AT5G26920	CBP60g	Zhang et al. 2010
Salicylic acid responsive	AT1G02450	NIMIN	Mohan et al. 2016
	AT1G22070	TGA3	Han et al. 2022
	AT1G64280	NPR1	Cao et al. 1994
	AT1G75040	PR5	Uknes et al. 1992
	AT1G77920	TGA7	Shearer et al. 2009
	AT2G14610	PR1	Uknes et al. 1992
	AT3G57260	PR2	Uknes et al. 1992

Appendix References

Cao H, Bowling SA, Gordon AS, Dong X (1994) Characterization of an Arabidopsis mutant that is nonresponsive to inducers of systemic acquired resistance. Plant Cell 6:1583–1592

Chen F, D’Auria JC, Tholl D, Ross JR, Gershenzon J, Noel JP, Pichersky E (2003) An Arabidopsis thaliana gene for methylsalicylate biosynthesis, identified by a biochemical genomics approach, has a role in defense. Plant J 36:577–588

Cui H, Gobbato E, Kracher B, Qiu J, Bautor J, Parker JE (2017) A core function of EDS1 with PAD4 is to protect the salicylic acid defense sector in Arabidopsis immunity. N Phytol 213:1802–1817

Han Q, Tan W, Zhao Y, Yang F, Yao X, Lin H, Zhang D (2022) Salicylic acid activated BIN2 phosphorylation of TGA3 promotes Arabidopsis PR gene expression and disease resistance. EMBO J 41:e110682

Mohan R, Tai T, Chen A, Arnoff T, Fu ZQ (2016) Overexpression of Arabidopsis NIMIN1 results in salicylate intolerance. Plant Signal Behav 11:e1211222

Rekhter D, Lüdke D, Ding Y, Feussner K, Zienkiewicz K, Lipka V, Wiermer M, Zhang Y, Feussner I (2019) Isochorismate-derived biosynthesis of the plant stress hormone salicylic acid. Science 365:498–502

Serrano M, Wang B, Aryal B, Garcion C, Abou-Mansour E, Heck S, Geisler M, Mauch F, Nawrath C, Métraux JP (2013) Export of salicylic acid from the chloroplast requires the multidrug and toxin extrusion-like transporter EDS5. Plant Physiol 162:1815–1821

Shearer HL, Wang L, De LongC, Despres C, Fobert PR (2009) NPR1 enhances the DNA binding activity of the Arabidopsis bZIP transcription factor TGA7. Botany 87:561–570

Torrens-Spence MP, Bobokalonova A, Carballo V, Glinkerman CM, Pluskal T, Shen A, Weng JK (2019) PBS3 and EPS1 complete salicylic acid biosynthesis from isochorismate in Arabidopsis. Mol Plant 12:1577–1586

Uknes S, Mauch-Mani B, Moyer M, Potter S, Williams S, Dincher S, Chandler D, Slusarenko A, Ward E, Ryals J (1992) Acquired resistance in Arabidopsis. Plant Cell 4:645–656

Wildermuth MC, Dewdney J, Wu G, Ausubel FM (2001) Isochorismate synthase is required to synthesize salicylic acid for plant defence. Nature 414:562–565

Zhang K, Halitschke R, Yin C, Liu CJ, Gan SS (2013) Salicylic acid 3-hydroxylase regulates Arabidopsis leaf longevity by mediating salicylic acid catabolism. Proc Natl Acad Sci USA 110:14807–14812

Zhang Y, Xu S, Ding P, Wang D, Cheng YT, He J, Gao M, Xu F, Li Y, Zhu Z, Li X, Zhang Y (2010) Control of salicylic acid synthesis and systemic acquired resistance by two members of a plant-specific family of transcription factors. Proc Natl Acad Sci USA 107:18220–18225
