# Supplementary figures and images for: Plasmodesmal closure elicits stress responses
Source: EMBO Rep. 2026 May 2;27(12):3231–51. doi: 10.1038/s44319-026-00789-2 (PMC13303860; doi:10.1038/s44319-026-00789-2)

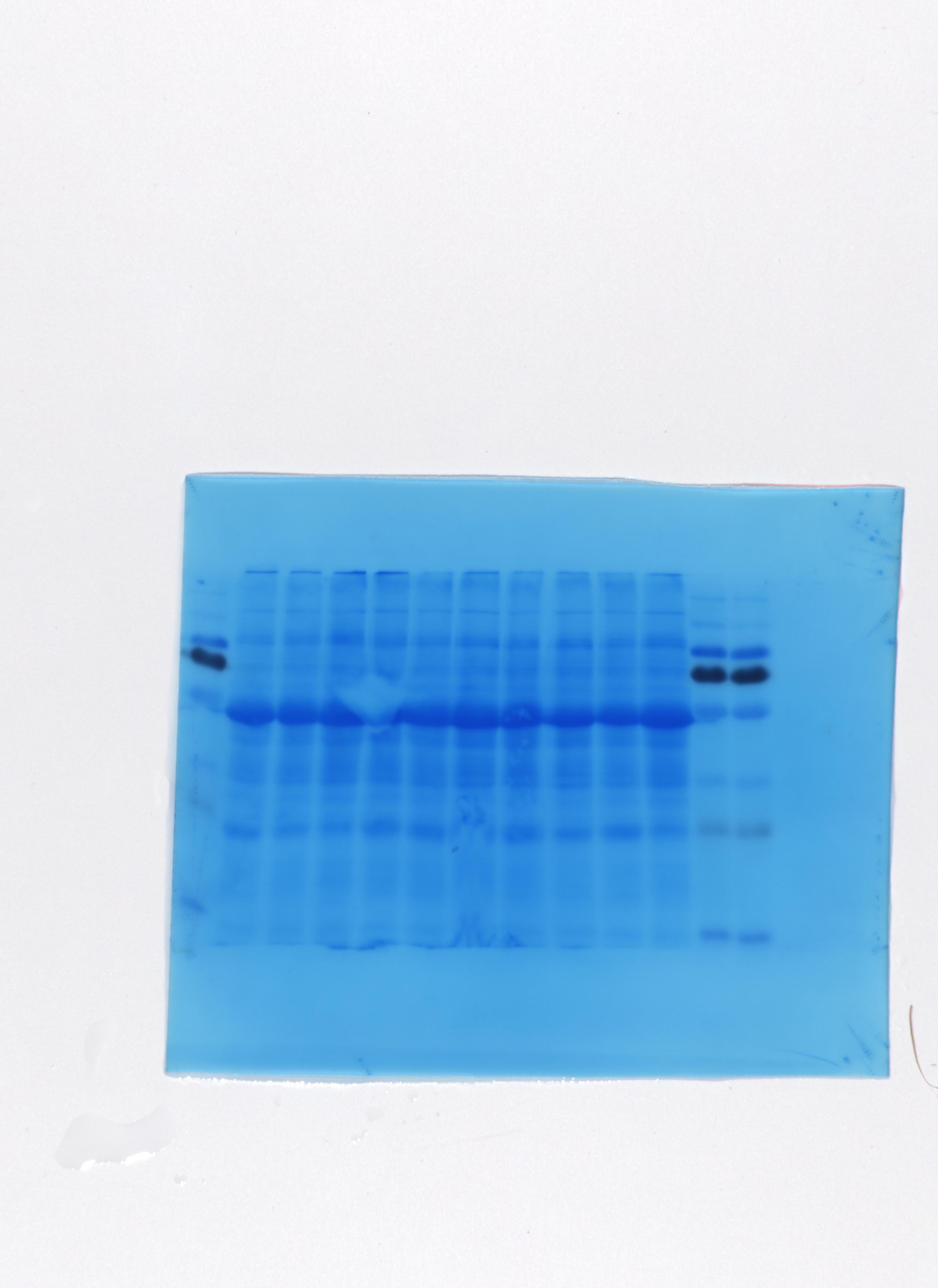

Supplement: Supplementary file 18 — Source data Fig. 3 [file 44319_2026_789_MOESM18_ESM.zip › 3C/Col-0_gel.jpg]

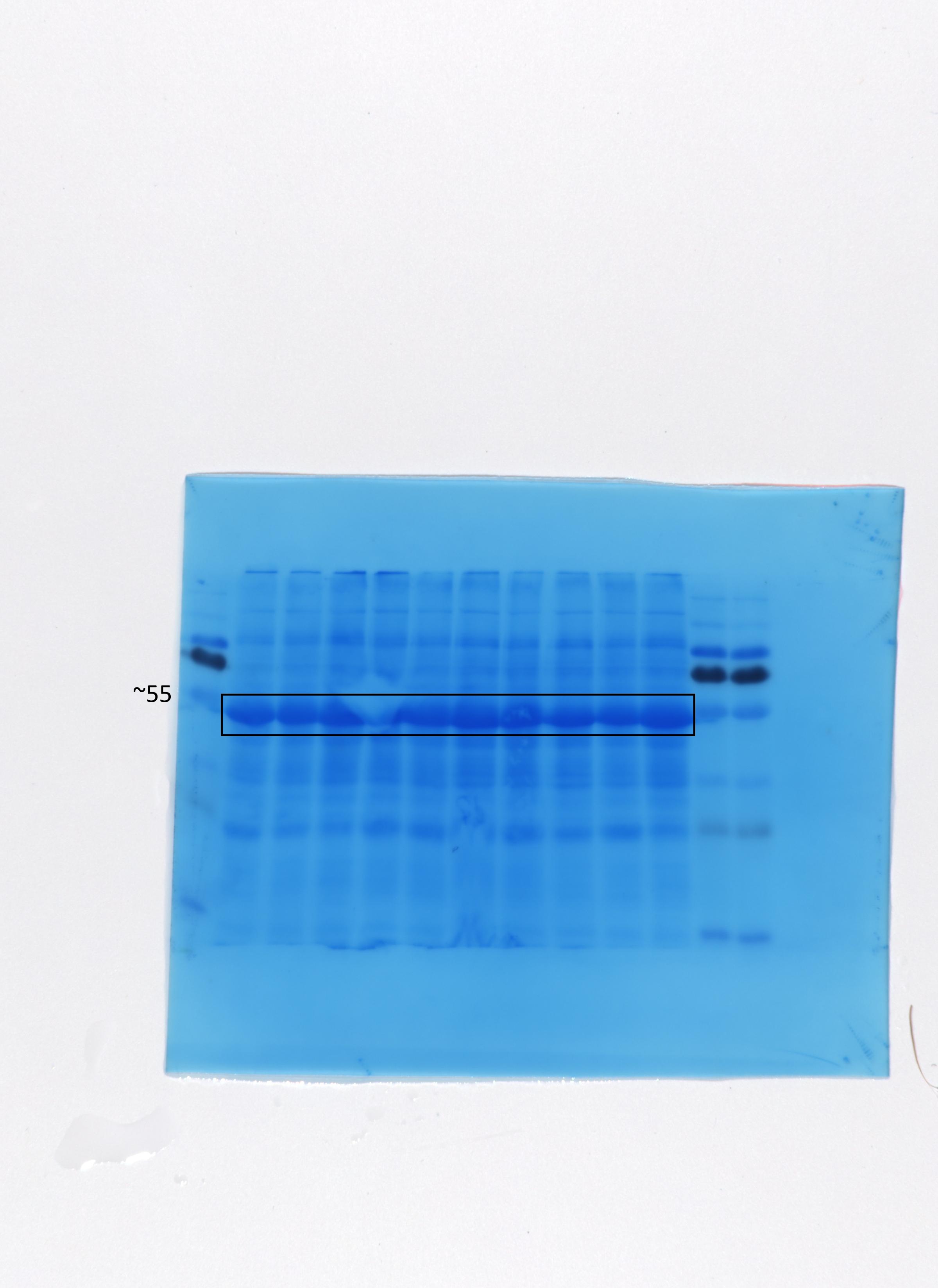

Supplement: Supplementary file 18 — Source data Fig. 3 [file 44319_2026_789_MOESM18_ESM.zip › 3C/Col-0_gel_labelled.jpg]

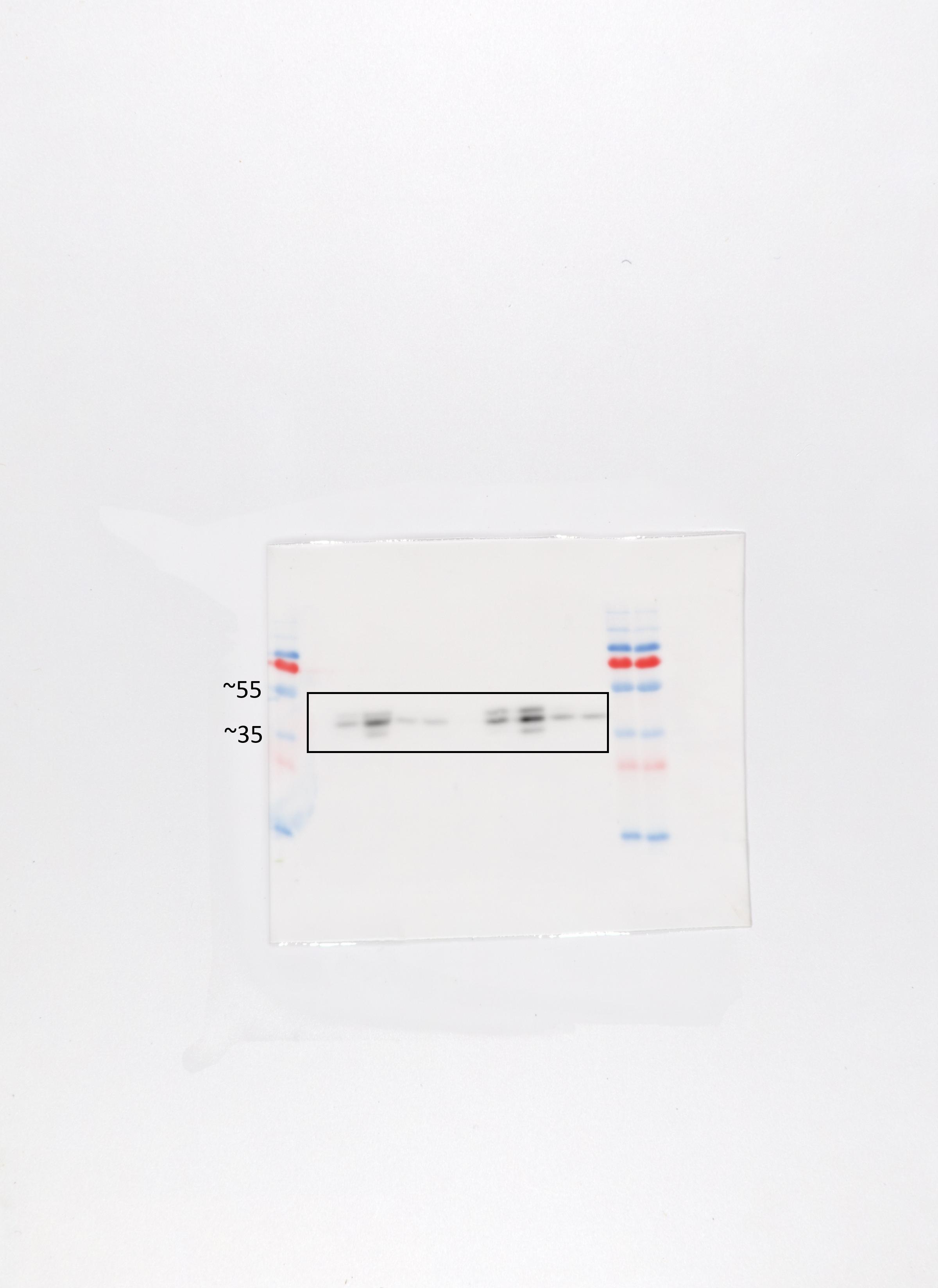

Supplement: Supplementary file 18 — Source data Fig. 3 [file 44319_2026_789_MOESM18_ESM.zip › 3C/Col-0_westernblot _labelled.jpg]

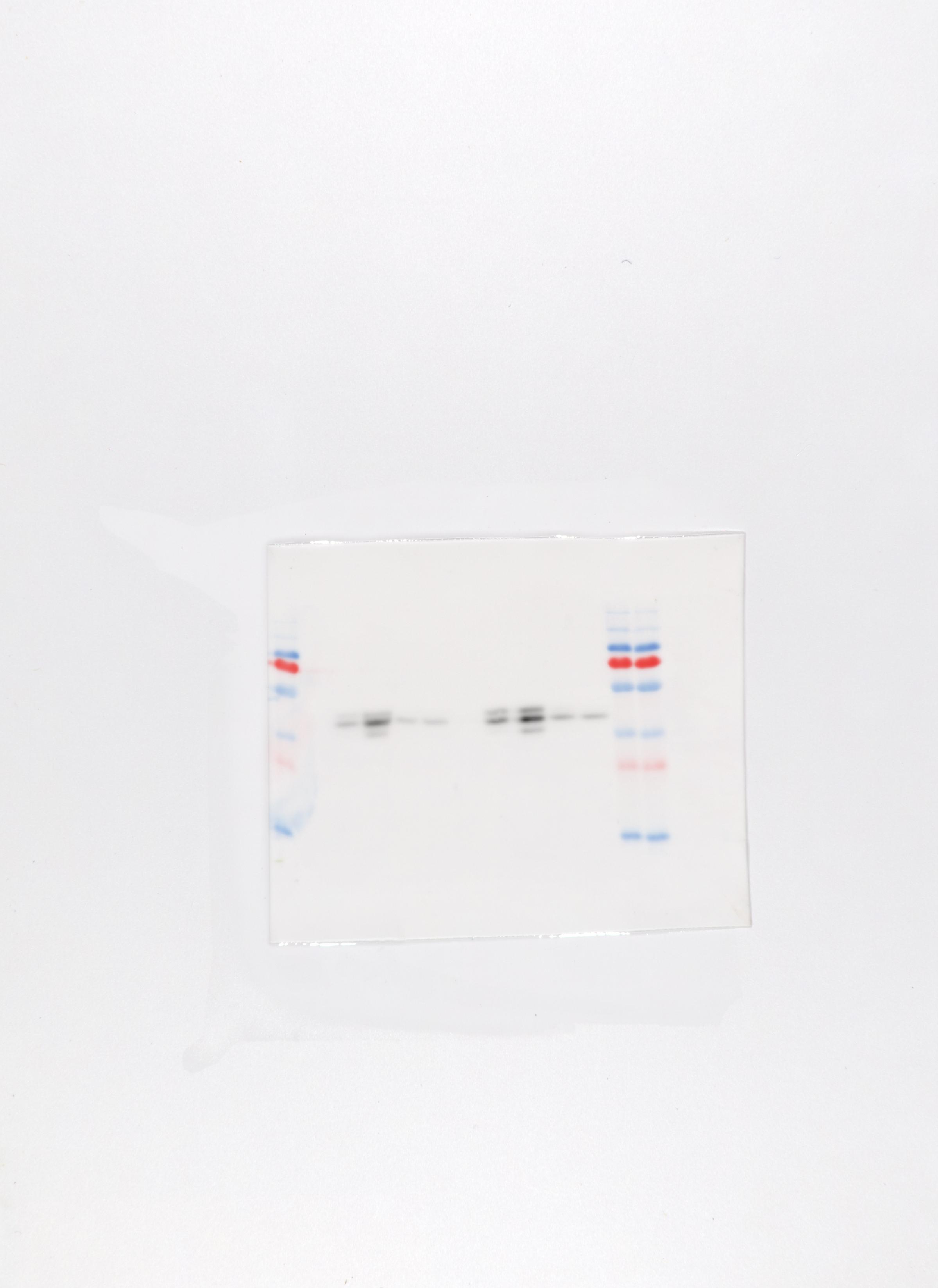

Supplement: Supplementary file 18 — Source data Fig. 3 [file 44319_2026_789_MOESM18_ESM.zip › 3C/Col-0_westernblot.jpg]

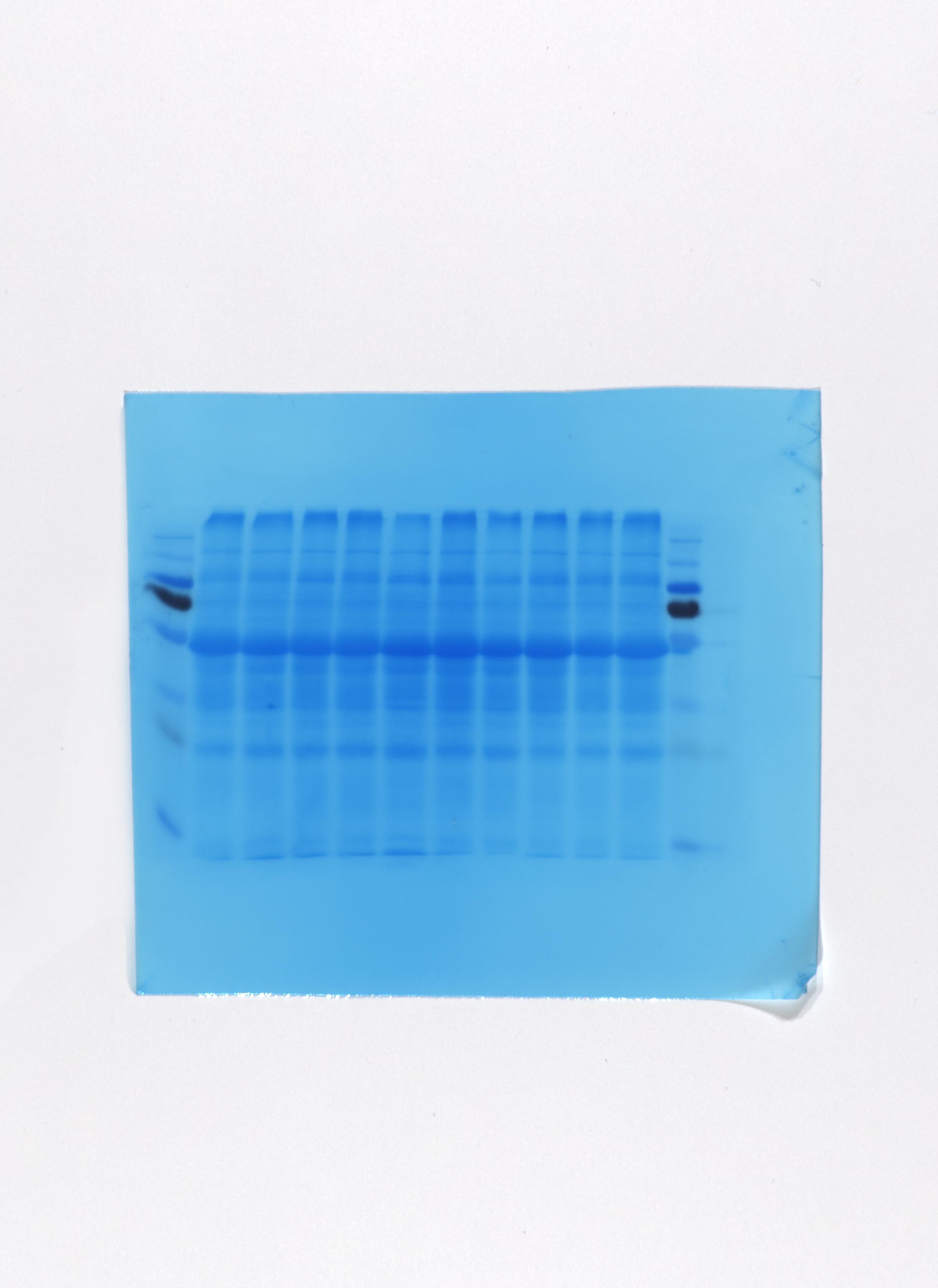

Supplement: Supplementary file 18 — Source data Fig. 3 [file 44319_2026_789_MOESM18_ESM.zip › 3C/icals3m_gel.jpg]

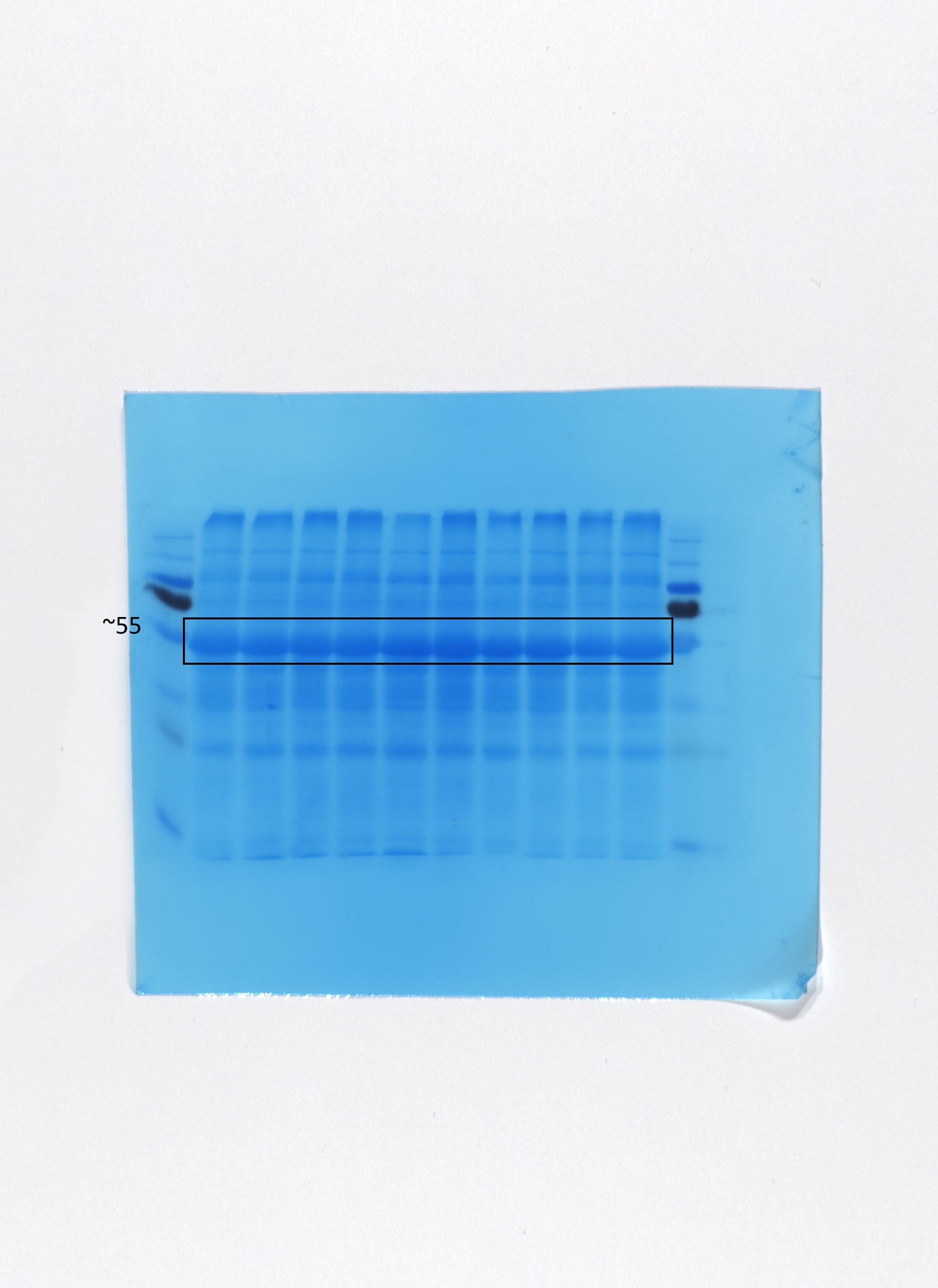

Supplement: Supplementary file 18 — Source data Fig. 3 [file 44319_2026_789_MOESM18_ESM.zip › 3C/icals3m_gel_labelled.jpg]

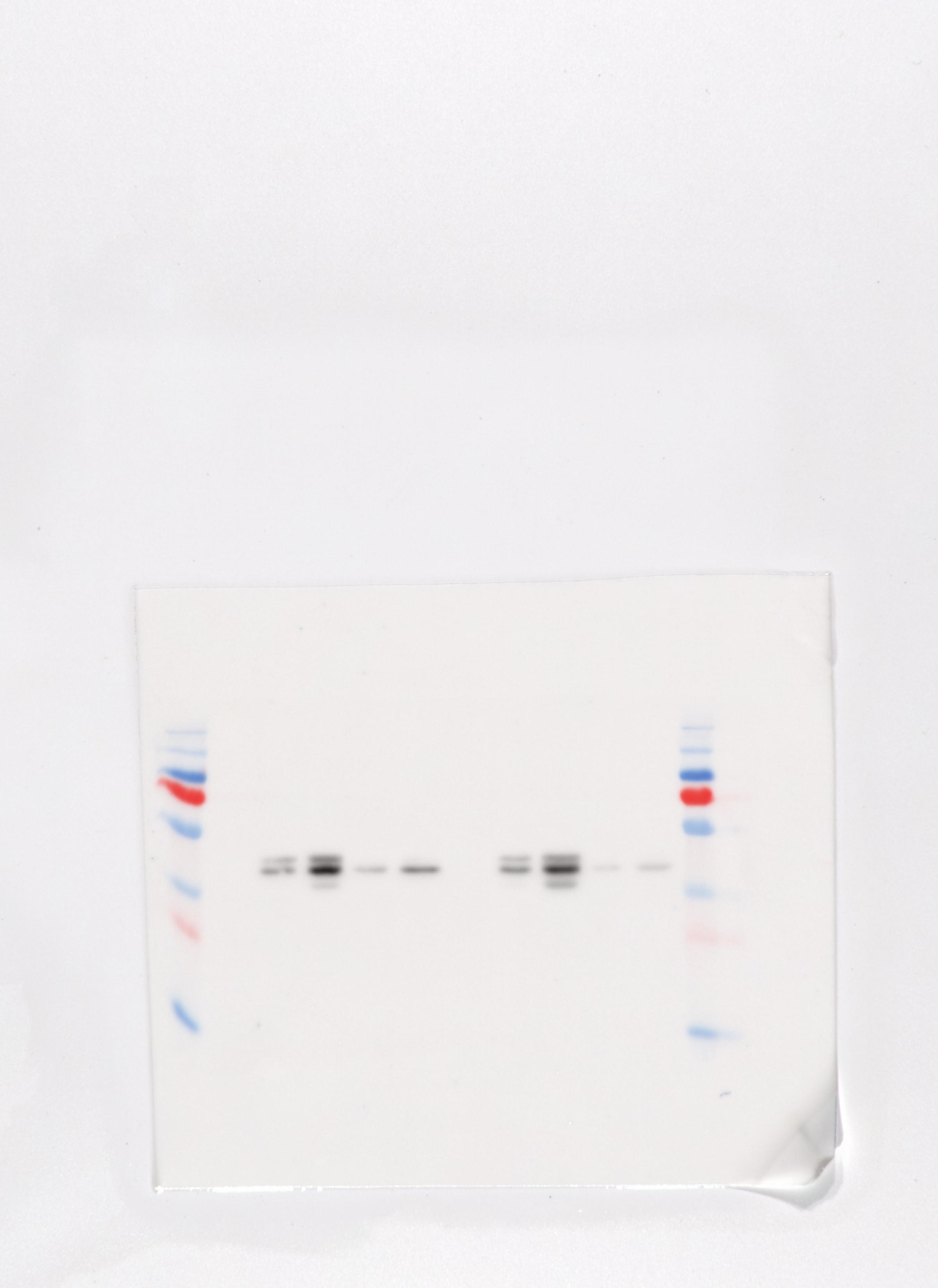

Supplement: Supplementary file 18 — Source data Fig. 3 [file 44319_2026_789_MOESM18_ESM.zip › 3C/icals3m_westernblot.jpg]

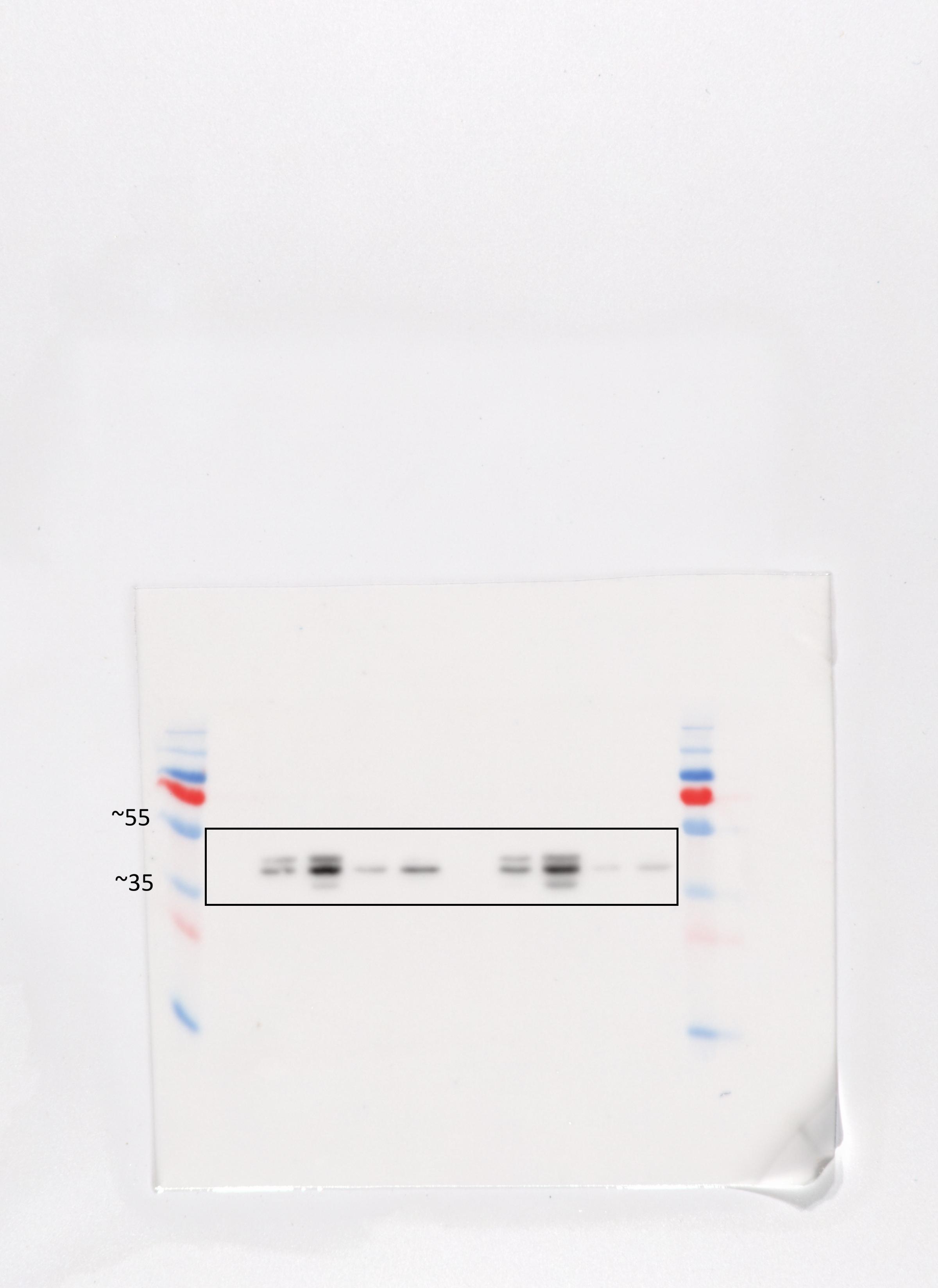

Supplement: Supplementary file 18 — Source data Fig. 3 [file 44319_2026_789_MOESM18_ESM.zip › 3C/icals3m_westernblot_labelled.jpg]

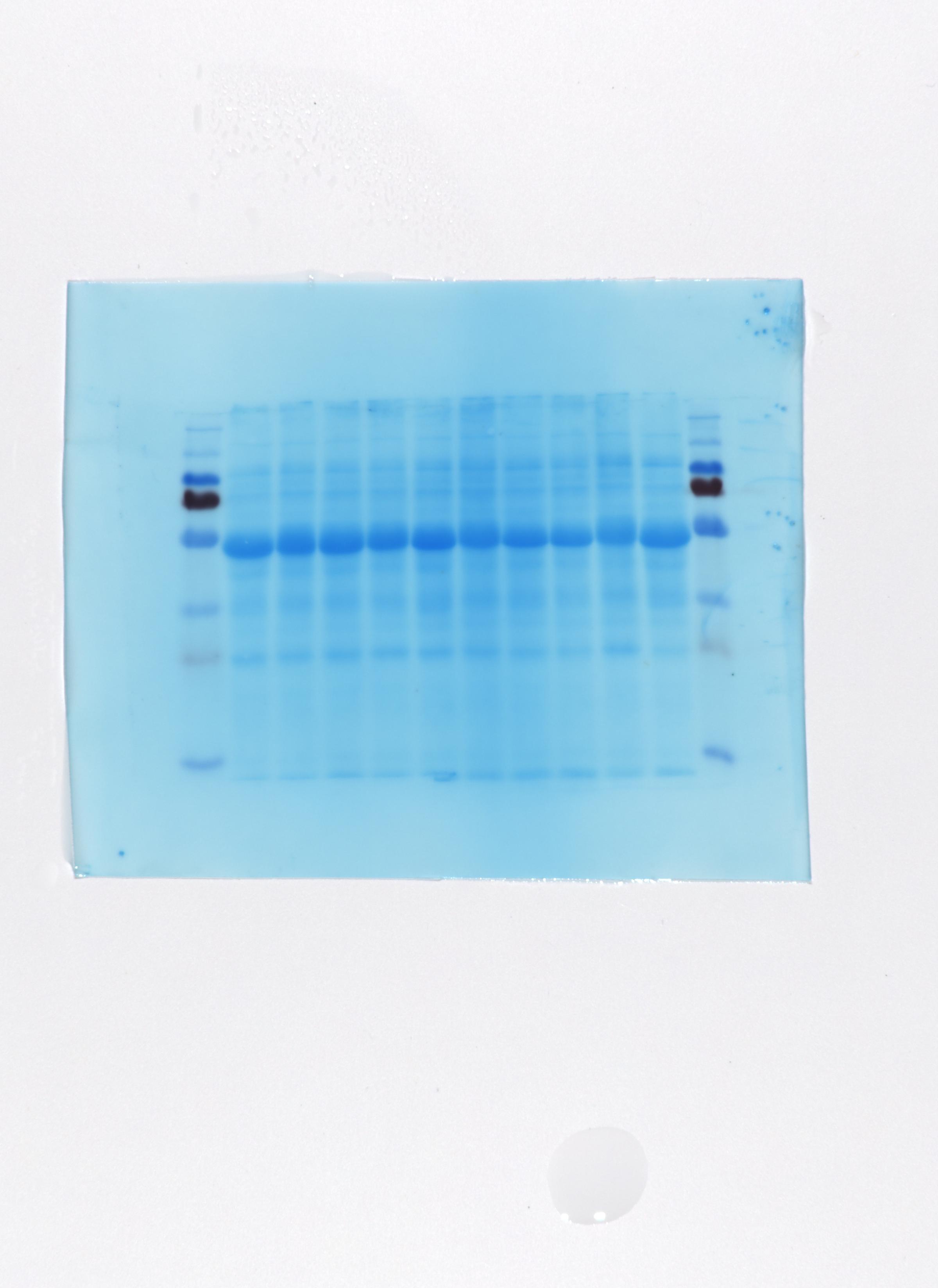

Supplement: Supplementary file 18 — Source data Fig. 3 [file 44319_2026_789_MOESM18_ESM.zip › 3C/PD-Plug_gel.jpg]

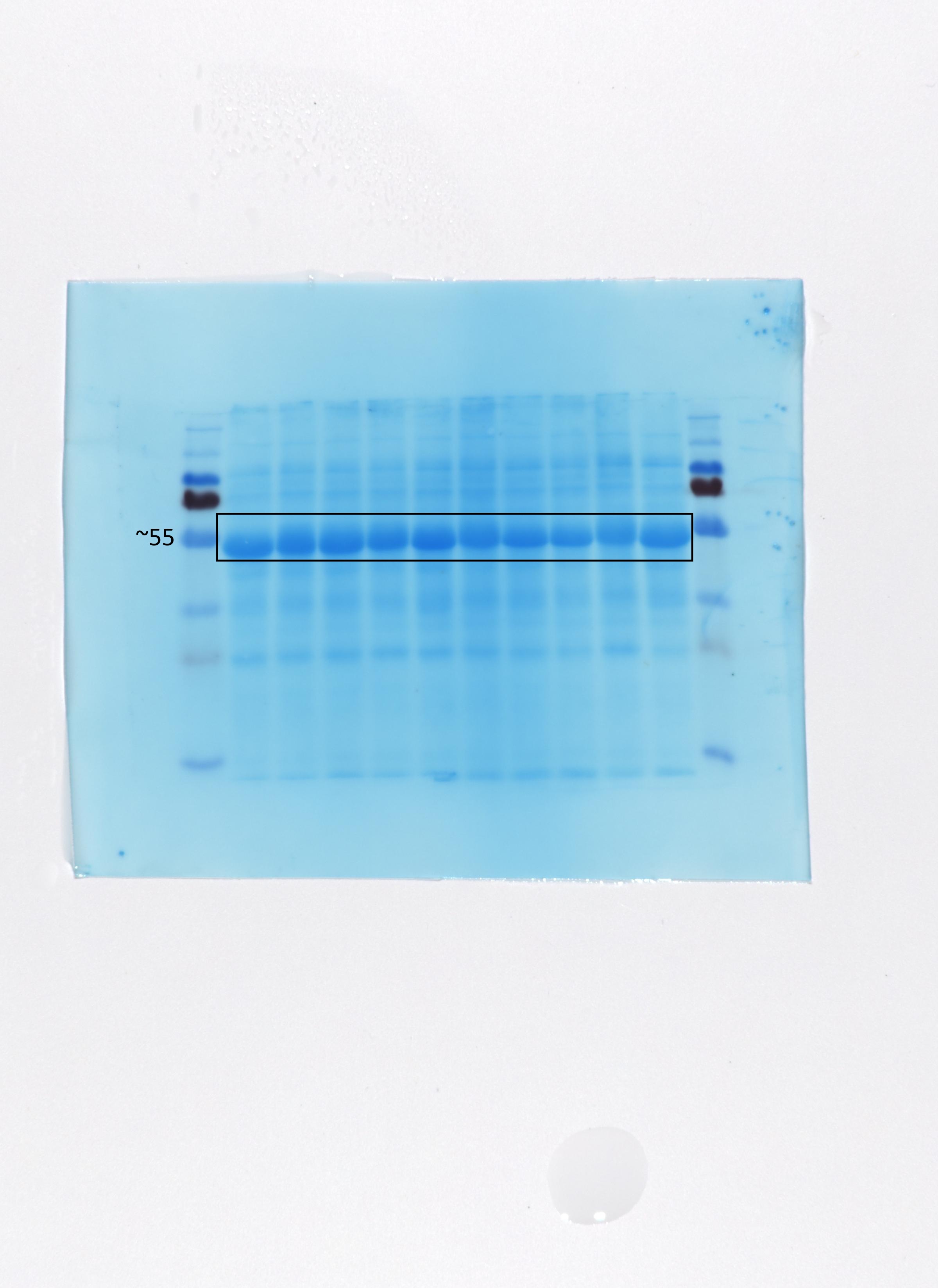

Supplement: Supplementary file 18 — Source data Fig. 3 [file 44319_2026_789_MOESM18_ESM.zip › 3C/PD-Plug_gel_labelled.jpg]

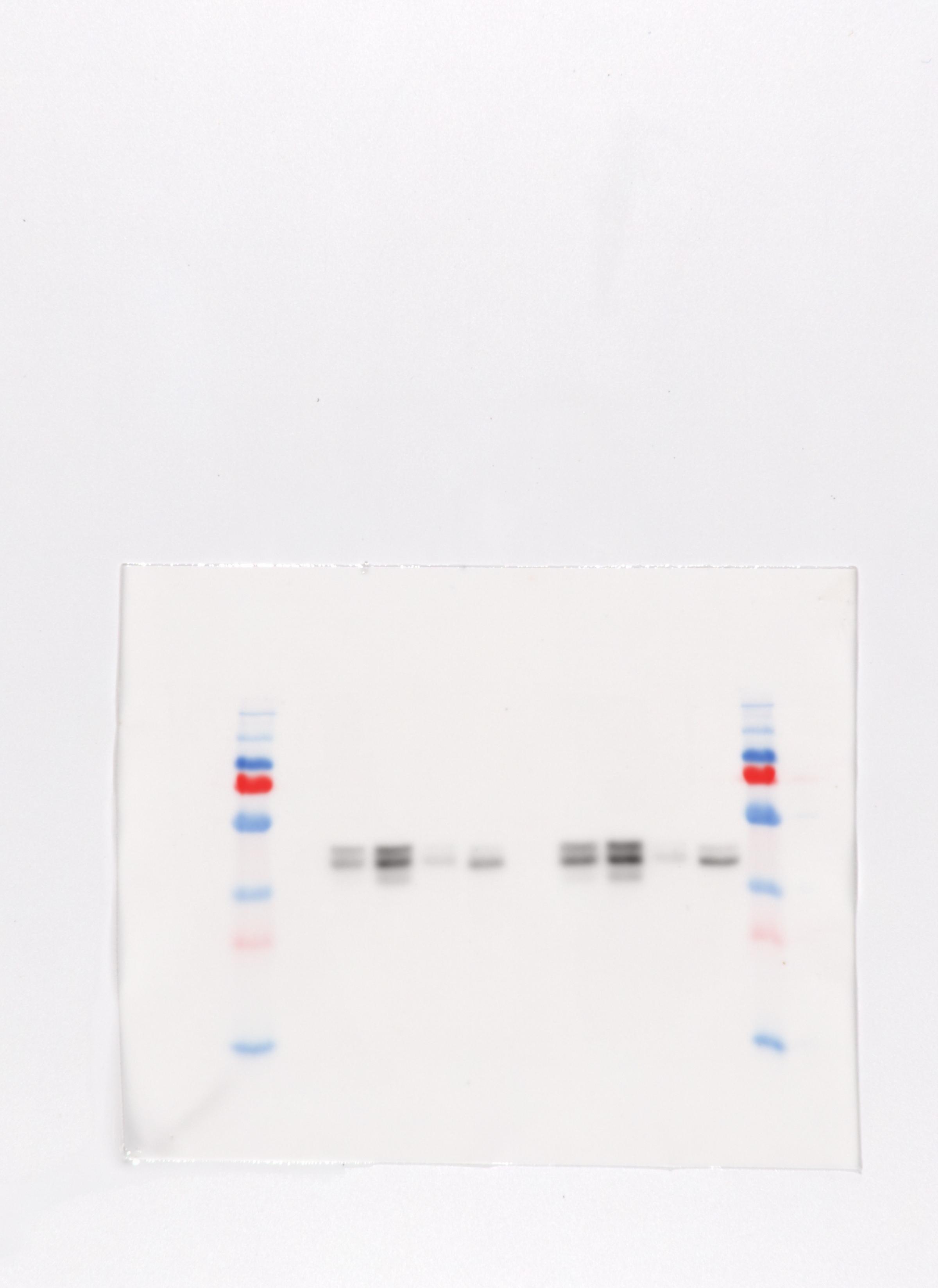

Supplement: Supplementary file 18 — Source data Fig. 3 [file 44319_2026_789_MOESM18_ESM.zip › 3C/PD-Plug_westernblot.jpg]

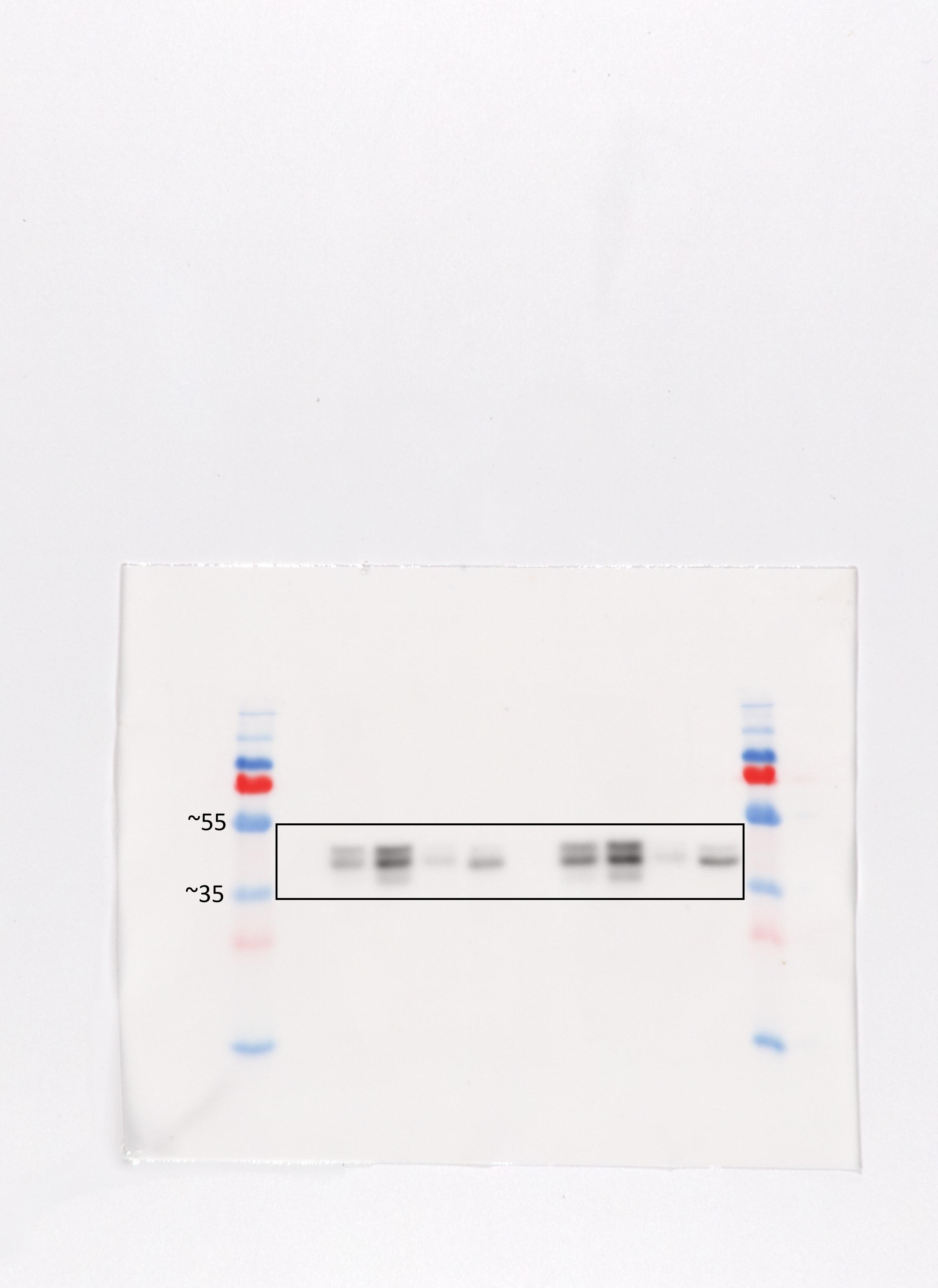

Supplement: Supplementary file 18 — Source data Fig. 3 [file 44319_2026_789_MOESM18_ESM.zip › 3C/PD-Plug_westernblot_labelled.jpg]

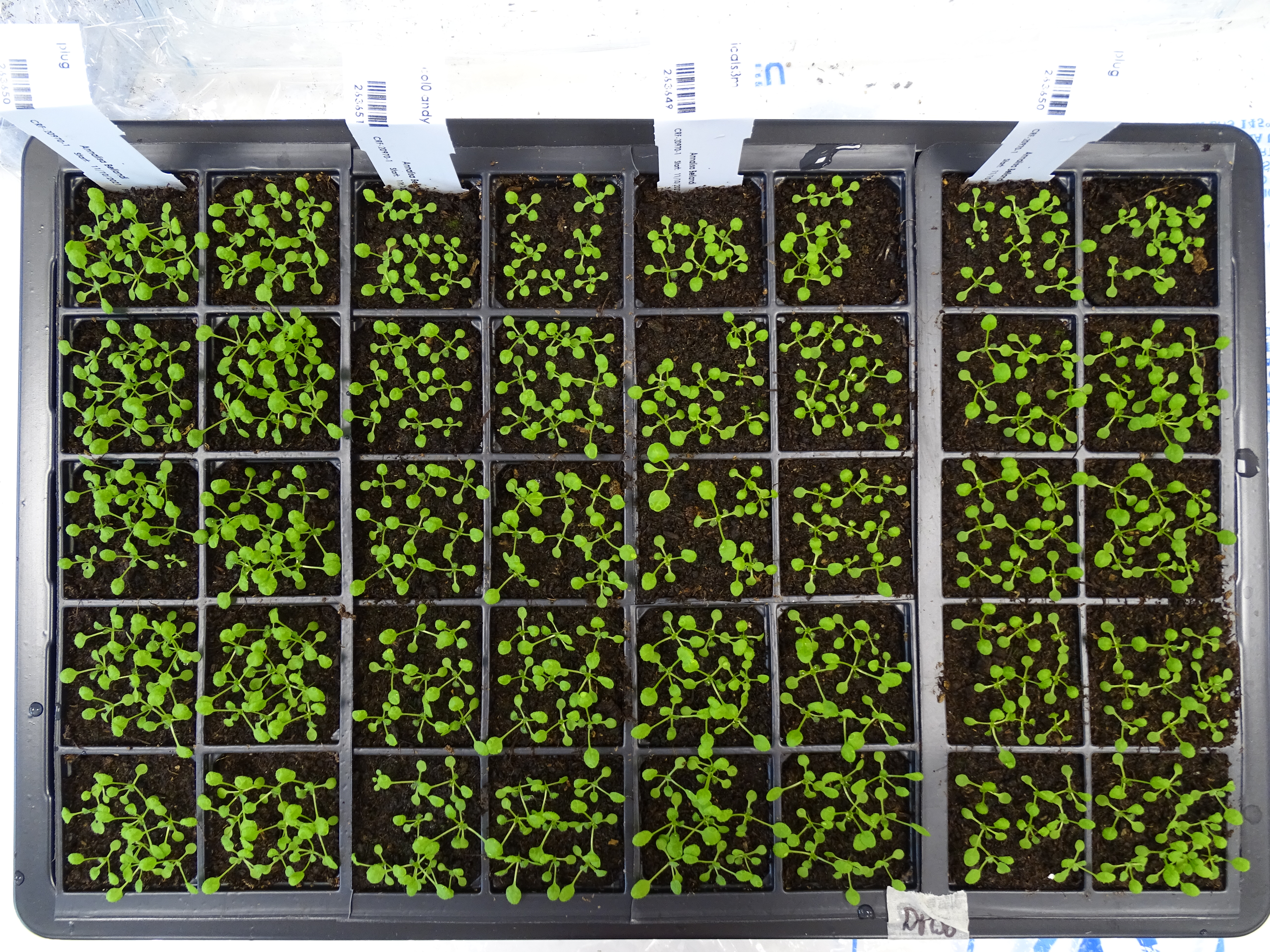

Supplement: Supplementary file 20 — Expanded View Figures Source Data [file 44319_2026_789_MOESM20_ESM.zip › Source Data for Expanded View and Appendix/Figure EV4/DMSO_treatment.JPG]

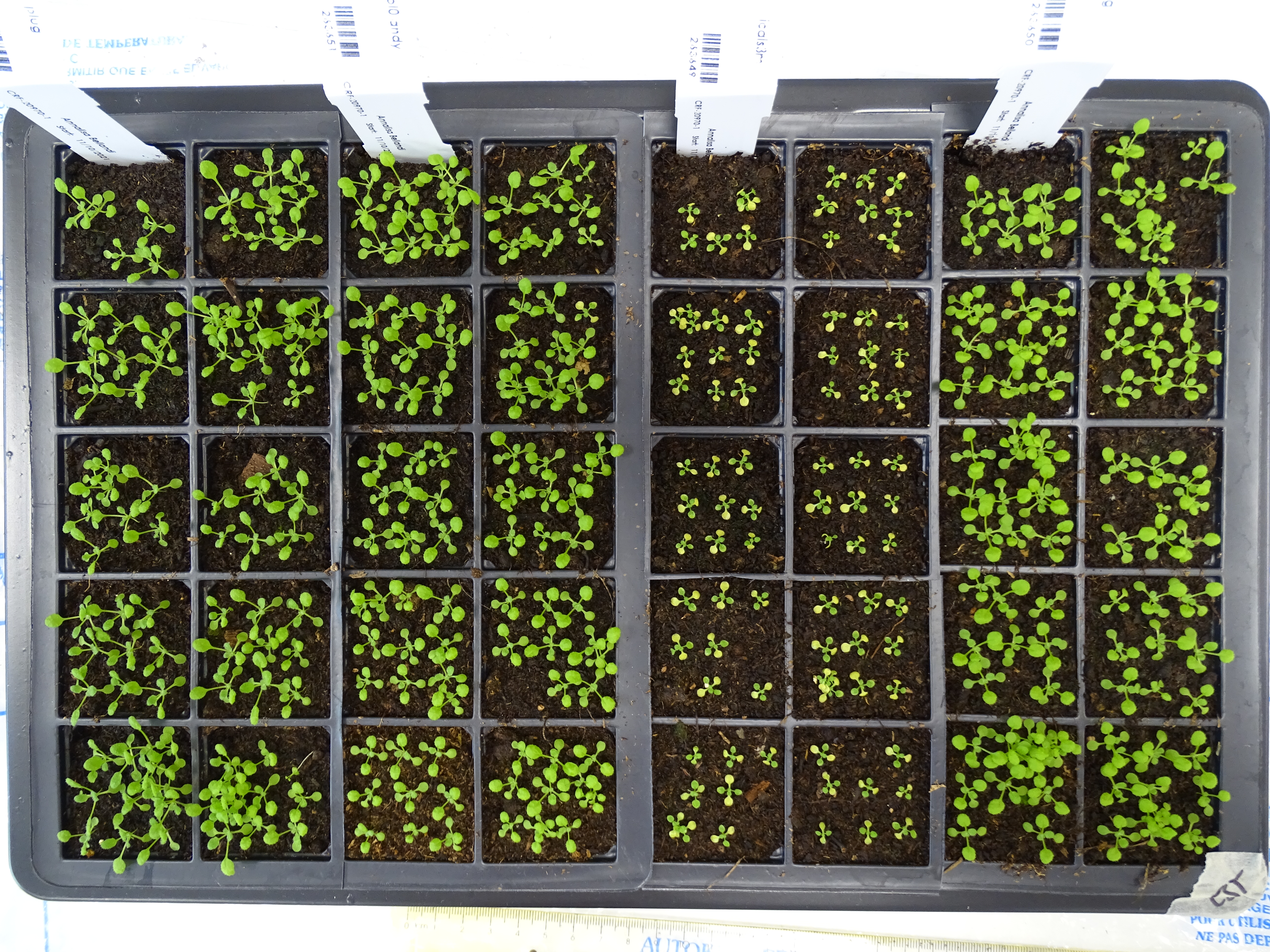

Supplement: Supplementary file 20 — Expanded View Figures Source Data [file 44319_2026_789_MOESM20_ESM.zip › Source Data for Expanded View and Appendix/Figure EV4/estradiol_treatment.JPG]

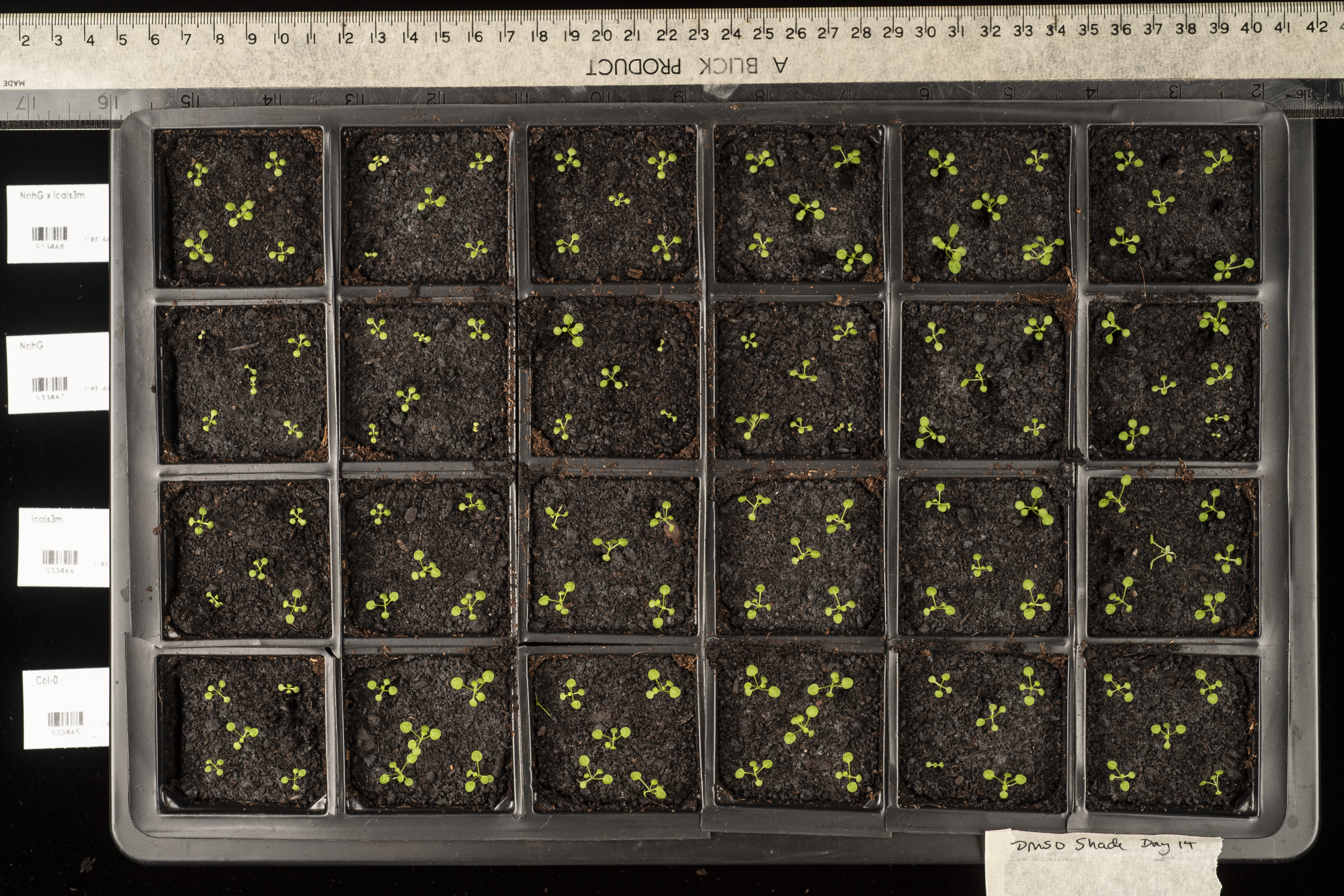

Supplement: Supplementary file 20 — Expanded View Figures Source Data [file 44319_2026_789_MOESM20_ESM.zip › Source Data for Expanded View and Appendix/Figure EV5/DMSO_shade.tif]

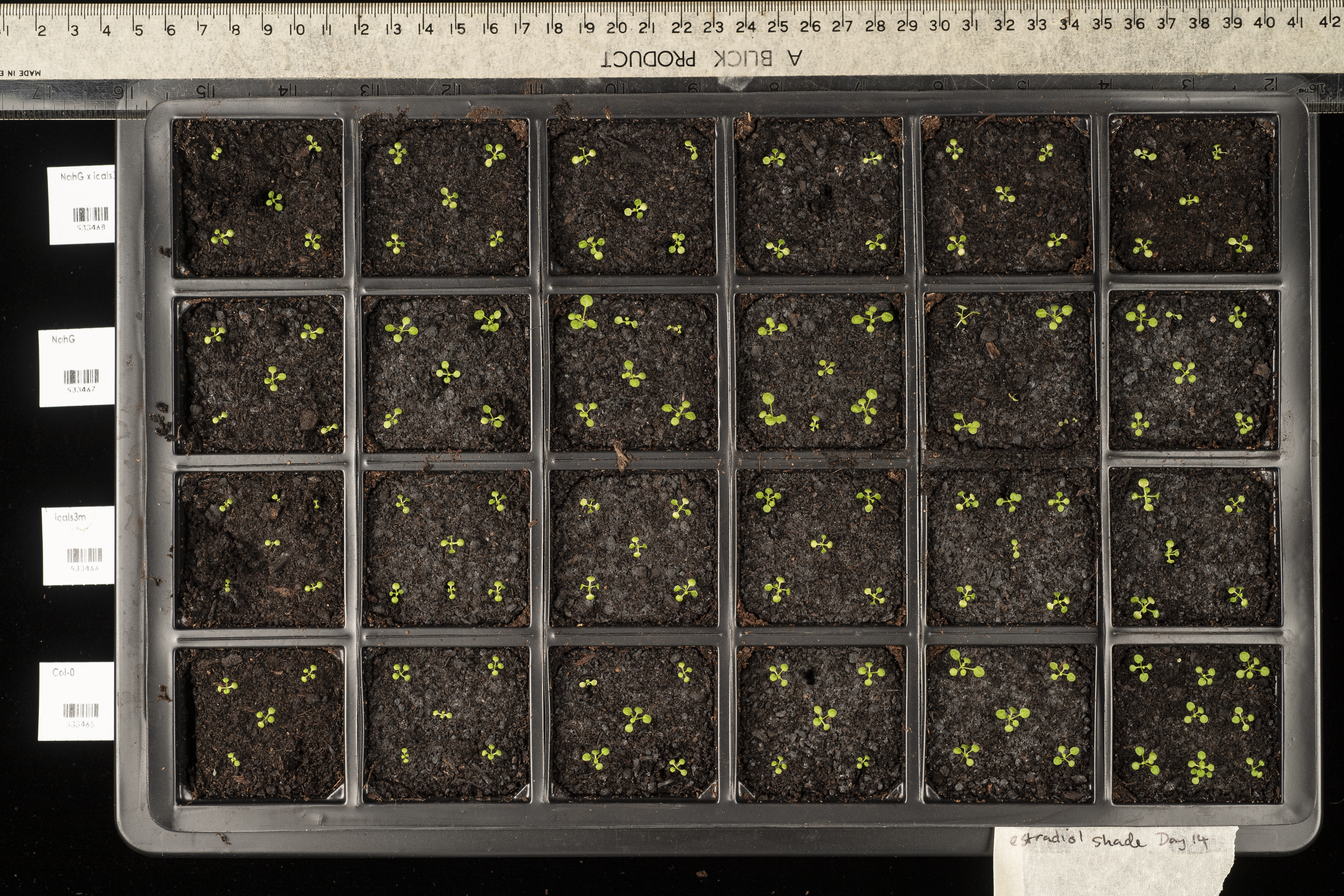

Supplement: Supplementary file 20 — Expanded View Figures Source Data [file 44319_2026_789_MOESM20_ESM.zip › Source Data for Expanded View and Appendix/Figure EV5/estradiol_shade.tif]

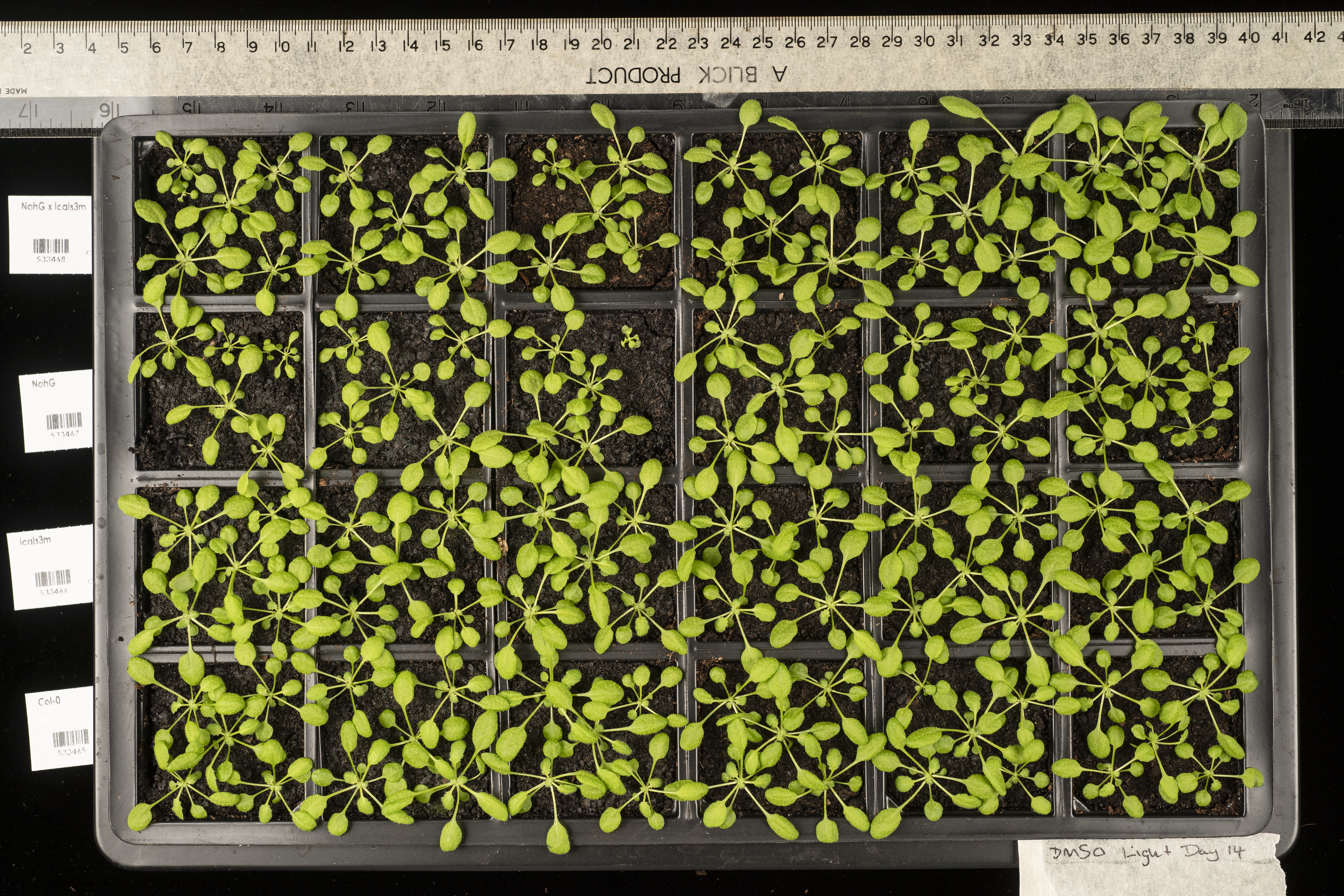

Supplement: Supplementary file 20 — Expanded View Figures Source Data [file 44319_2026_789_MOESM20_ESM.zip › Source Data for Expanded View and Appendix/Figure EV5/DMSO_light.tif]

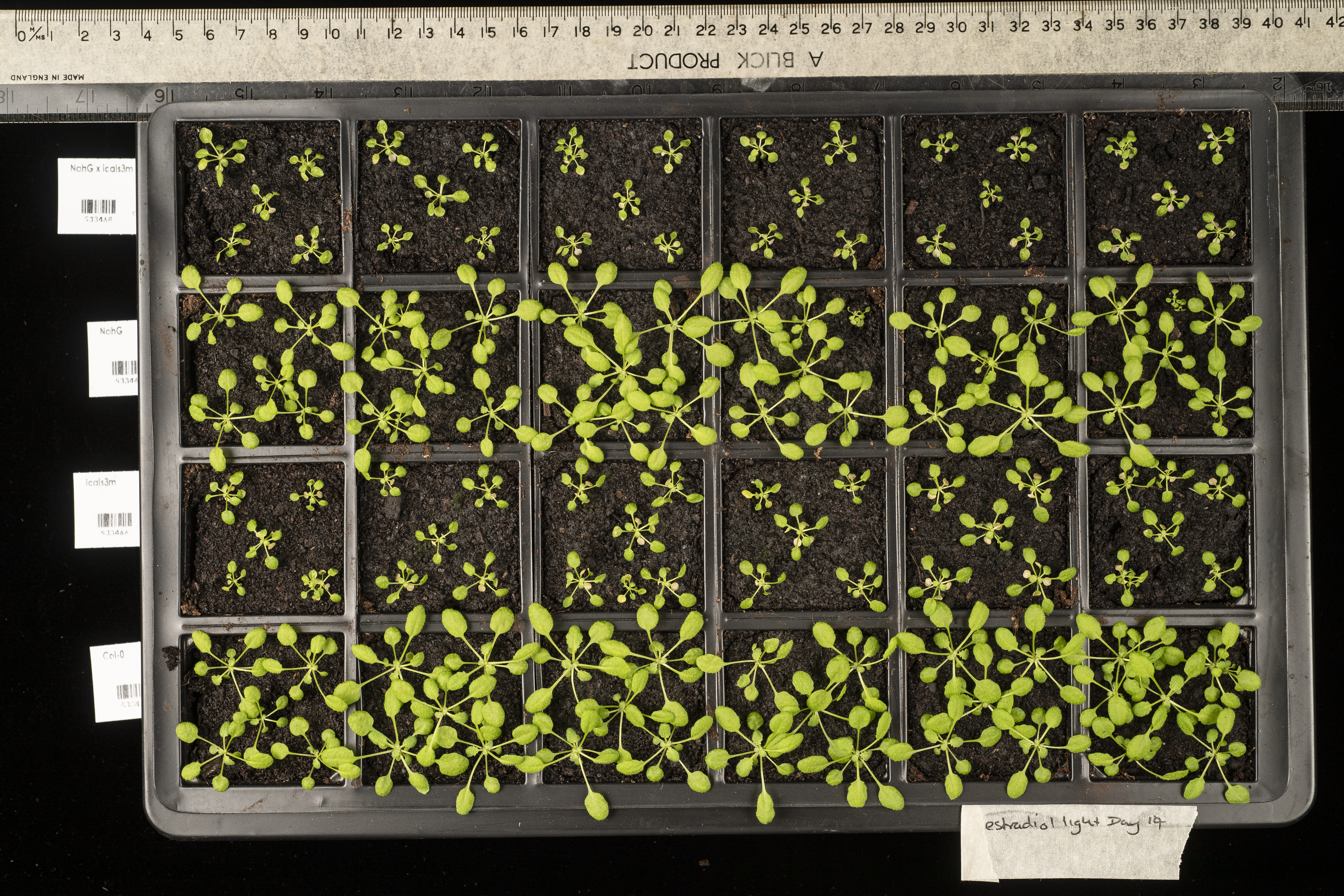

Supplement: Supplementary file 20 — Expanded View Figures Source Data [file 44319_2026_789_MOESM20_ESM.zip › Source Data for Expanded View and Appendix/Figure EV5/estradiol_light.tif]
