# Supplementary material for: Plasmodesmal closure elicits stress responses
Source: EMBO Rep. 2026 May 2;27(12):3231–51. doi: 10.1038/s44319-026-00789-2 (PMC13303860; doi:10.1038/s44319-026-00789-2)
Supplement: Supplementary file 21 — Expanded View Figures [file 44319_2026_789_MOESM21_ESM.pdf]

Expanded View Figures

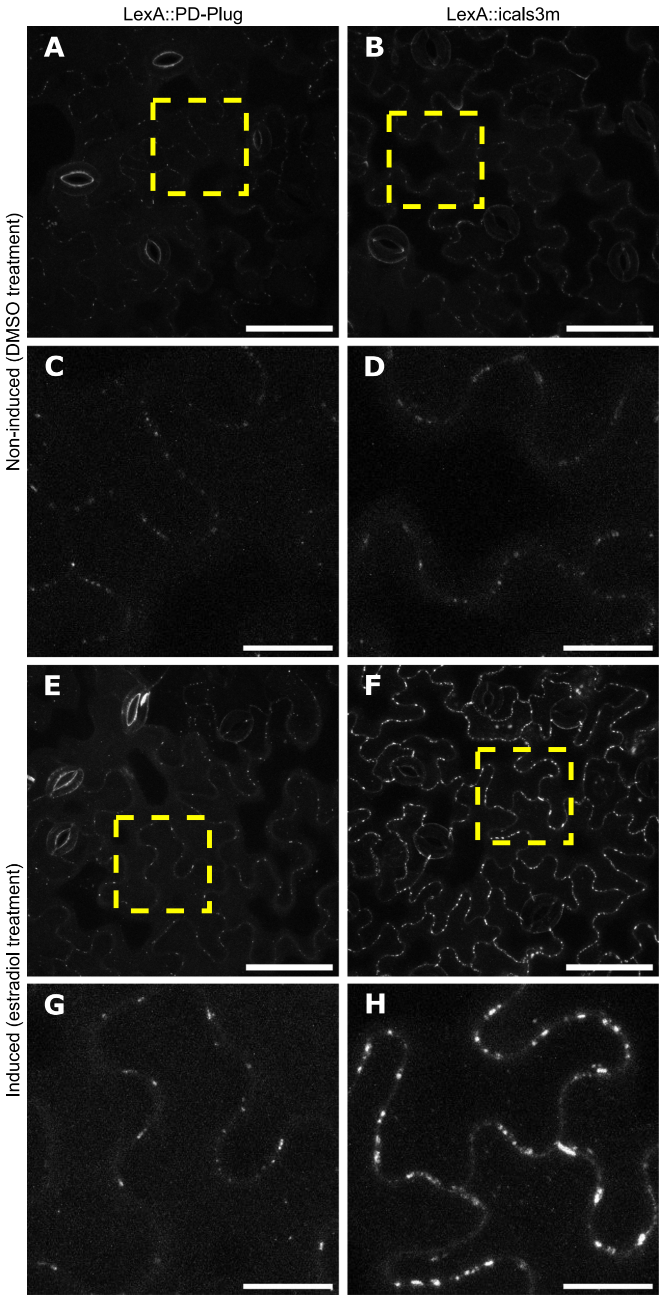

Figure EV1. Aniline blue-stained callose at plasmodesmata in LexA::PD-Plug and LexA::icals3m.

LexA::PD-Plug and LexA::icals3m treated with either DMSO (**A–D**) or estradiol (**E–H**) and infiltrated with aniline blue 24 h post treatment. (**C**) and (**D**) are zoomed in portions of (**A**, **B**), and (**G**, **H**) is zoomed in portion of (**E**, **F**) as indicated by the yellow square. (**A**, **B**) and (**E**, **F**) scale bar = 50 μM; (**C**, **D**) and (**G**, **H**) scale bar = 15 μM.

 © The Author(s)

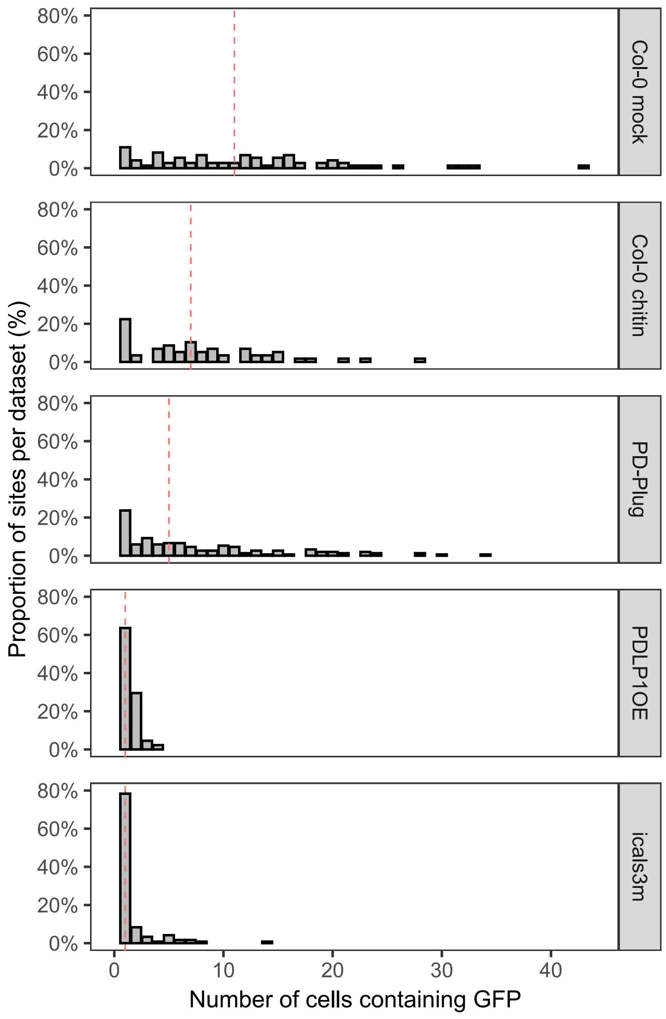

Figure EV2. Microprojectile bombardment data in different conditions and genotypes.

Comparison of microprojectile bombardment data showing GFP movement into neighboring cells, with datasets showing high variance and heterogeneity (i.e., Col-0 mock, Col-0 chitin and estradiol treated LexA::PD-Plug [PD-Plug]) in comparison to low variance and little GFP movement (i.e., PDLP1OE and estradiol treated LexA::icals3m [icals3m]). Data represented taken from Cheval et al (2020) for Col-0 mock and Col-0 chitin, and Tee et al (2023b) for PDLP1OE. Red dotted line indicates median in a given dataset.

© The Author(s)

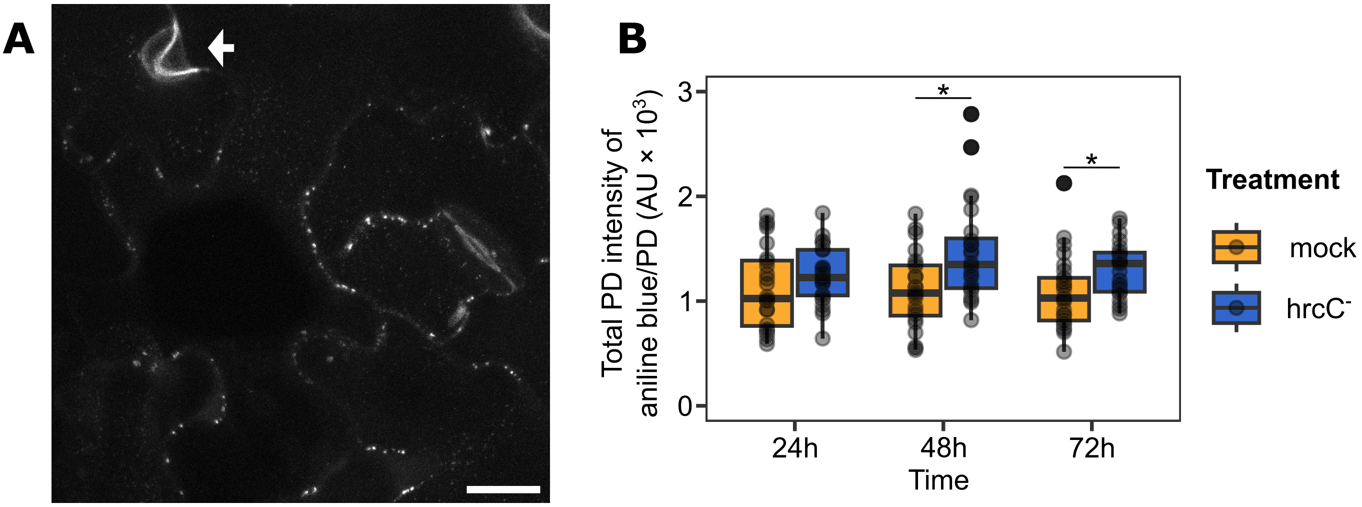

Figure EV3. ***Pseudomonas syringae*** **DC3000 mutant strain** ***hrcC⁻*** **infection in Col-0.**

(**A**) Example aniline blue-stained callose deposition at plasmodesmata as well as macroscopic callose deposits (labeled by white arrow) with the *Pseudomonas syringae hrcC⁻* mutant (*hrcC⁻*) treatment. Scale bar = 15 µm. (**B**) Quantification of aniline blue-stained plasmodesmata-associated callose in 5-week-old Col-0 plants 24 h, 48 h, and 72 h post treatment of H_2O (mock) or *hrcC⁻*. Datapoints represent the average of the total PD intensity/plasmodesmata (PD) per image, with $n = 24$–25 images from 8 biological replicates per treatment/timepoint. Bootstrap analysis indicates significant differences between mock and *hrcC⁻* treatment at 48 h and 72 h, as indicated by $^{*}p < 0.05$. For (**B**), the center line marks the median, the box indicates the upper and lower quartiles, and the whiskers show the minimum and maximum values within 1.5× interquartile range.

 © The Author(s)

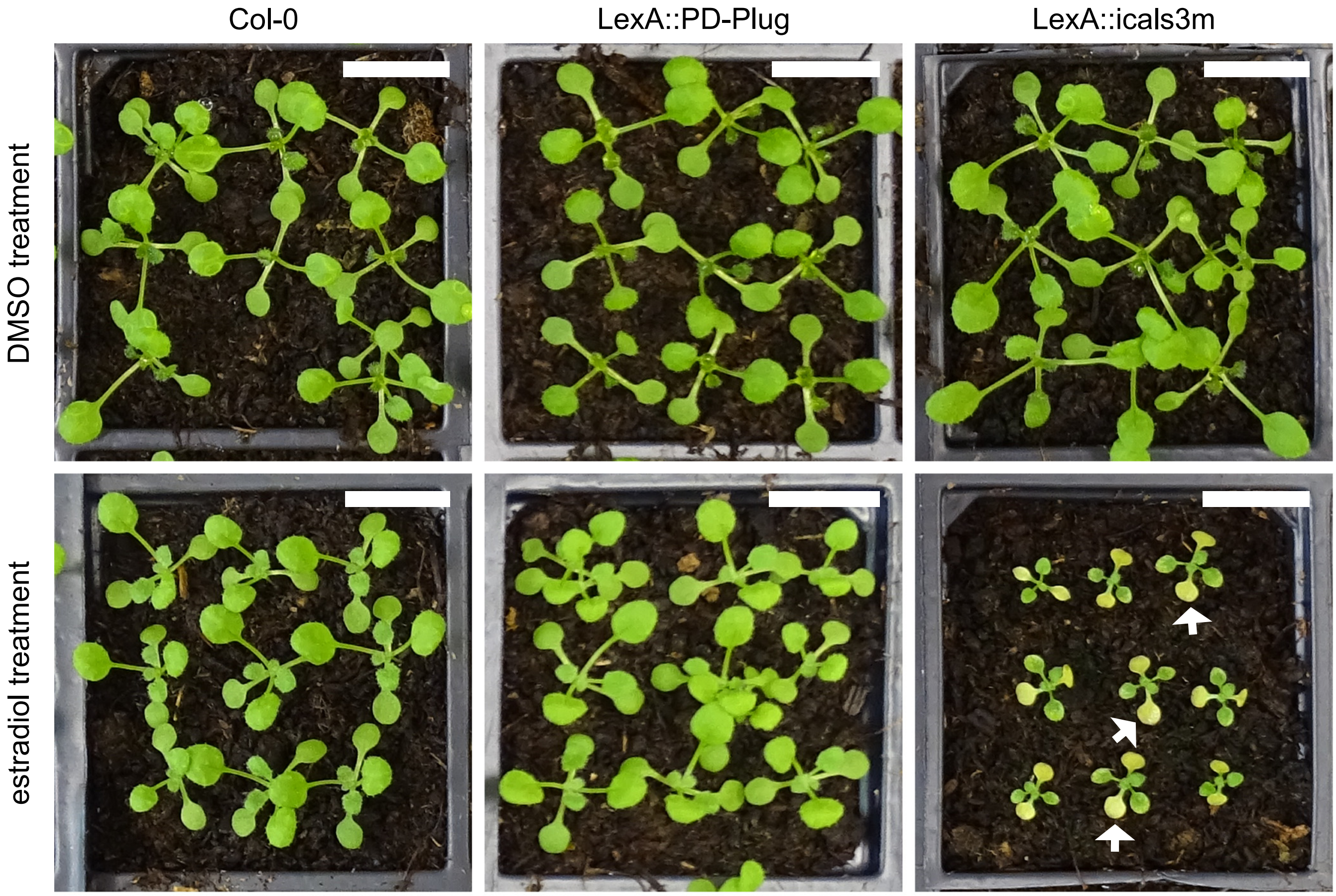

Figure EV4. Photographs of 15-day-old plants showing the growth phenotype of Col-0, LexA::PD-Plug, LexA::icals3m induced by DMSO or estradiol treatment.

White arrows indicate yellowing/senescence on leaves. Scale bar = 15 mm.

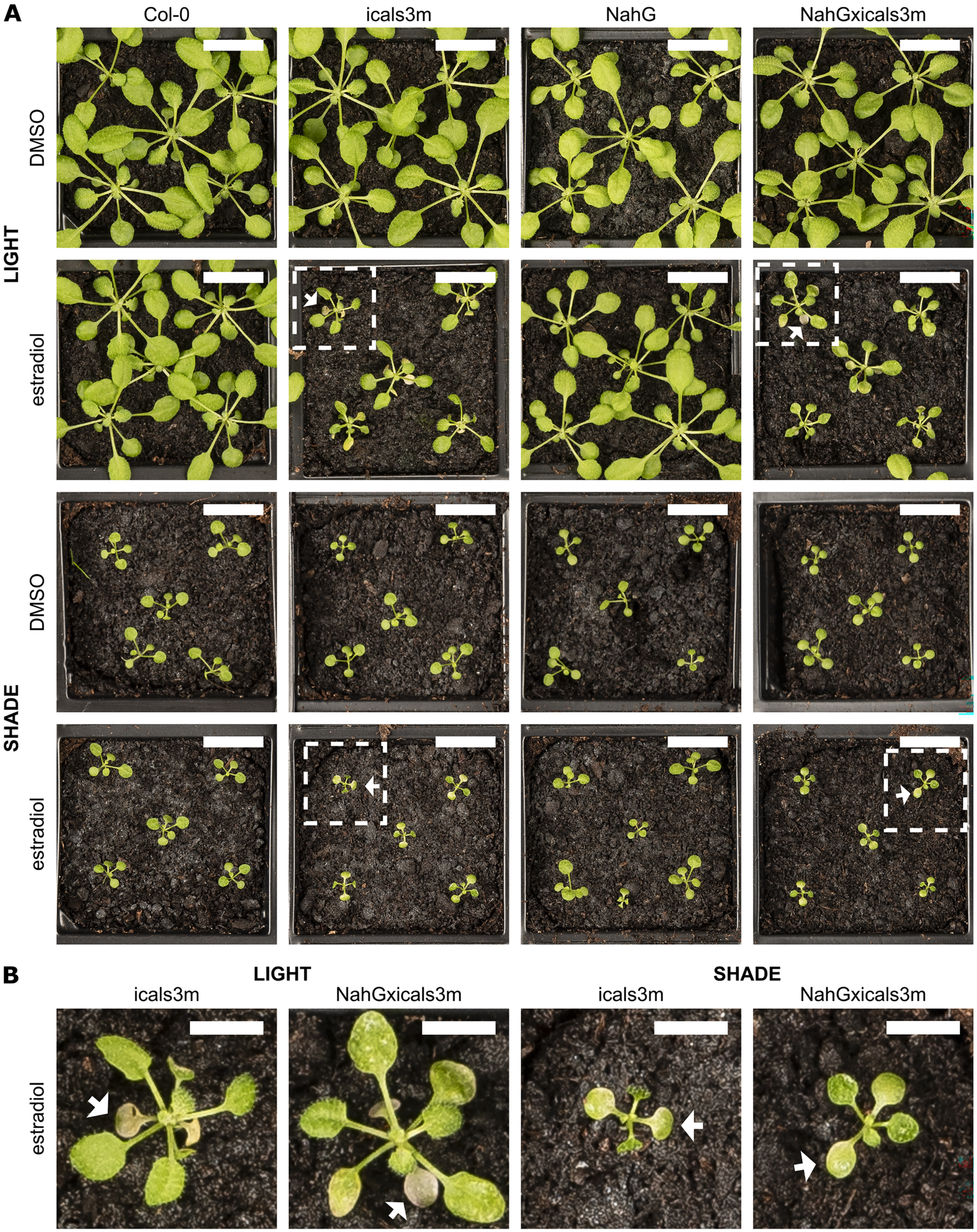
A
Col-0
icals3m
NahG
NahGxicals3m
LIGHT
DMSO
estradiol
SHADE
DMSO
estradiol
B
LIGHT
SHADE
icals3m
NahGxicals3m
icals3m
NahGxicals3m
estradiol

 © The Author(s)

Figure EV5. Photographs of 24-day-old plants showing the growth phenotype of Col-0, LexA::icals3m (icals3m), NahG and NahG×LexA::icals3m (NahG×icals3m) induced by DMSO or estradiol treatment under light or shaded conditions.

White arrows indicate example yellowing/senescence on leaves. (**B**) is zoomed in portion of (**A**) as indicated by the white square. (**A**) scale bar = 15 mm, (**B**) scale bar = 5 mm.

© The Author(s)
