# Supplementary material for: Plasmodesmal closure elicits stress responses
Source: EMBO Rep. 2026 May 2;27(12):3231–51. doi: 10.1038/s44319-026-00789-2 (PMC13303860; doi:10.1038/s44319-026-00789-2)
Supplement: Supplementary file 2 — Peer Review File [file 44319_2026_789_MOESM2_ESM.pdf]

EMBO reports

Plasmodesmal closure elicits stress responses

Estee Tee, Andrew Breakspear, Diana Papp, Hannah Thomas, Catherine Walker, Annalisa Bellandi, and Christine Faulkner

Corresponding author(s): Christine Faulkner (Christine.Faulkner@jic.ac.uk)

Review Timeline:

Transfer Date:	20th Oct 25
Editorial Decision:	19th Dec 25
Revision Received:	23rd Mar 26
Accepted:	13th Apr 26

Editor: Martina Rembold

Transaction Report: This manuscript was transferred to EMBO Reports following peer review at The EMBO Journal.

(Note: With the exception of the correction of typographical or spelling errors that could be a source of ambiguity, letters and reports are not edited. Depending on transfer agreements, referee reports obtained elsewhere may or may not be included in this compilation. Referee reports are anonymous unless the Referee chooses to sign their reports.)

Referee #1:

This manuscript addresses the question of what kind of physiological responses happen in response to artificial plasmodesmata closure in order to understand the role of plasmodesmata closure in plant immunity.

The authors used approaches where transgenic plants induce callose deposition at plasmodesmata leading to plasmodesmata closure upon estradiol treatment. They found that salicylic acid (SA) and sugar accumulation increase associated with increased resistance against the biotrophic bacterial pathogen Pseudomonas syringae and with increased susceptibility to the necrotrophic fungal pathogen Botrytis cinerea.

Plasmodesmata close during pathogen infection but its role remains uncertain. The authors tackled the significant question by using a cool approach and revealed physiological responses. While their findings are novel and significant even though it has been known that constitutive plasmodesmata closure leads to SA increase, I feel that the story is incomplete for this journal. I find two major gaps in this study that need to be deciphered in the role of plasmodesmata closure in plant immunity. The first point is the mechanism by which plants actually trigger physiological responses such as SA and sugar increase upon plasmodesmata closure. The second point is what physiological responses are relevant for pathogen resistance. While I think that the first point might be beyond the scope of this study, the second point can be experimentally addressed.

(Major comments)
It makes sense that increased SA upon plasmodesmata closure causes increased resistance against P. syringae, which is sensitive to SA-mediated immunity. In addition, increased SA can lead to decreased jasmonate (JA) signaling due to SA-JA crosstalk, which likely causes increased susceptibility to B. cinerea, which is sensitive to JA-mediated immunity. The authors can test these hypotheses by generating transgenic plants in SA and JA-deficient mutant backgrounds. If possible (maybe unrealistic), it would be great if the authors can also block sugar increase upon plasmodesmata closure by introducing some mutation. Then, they can measure SA and sugar in those conditions and perform pathogen assays. These will address the cause of changed pathogen resistance upon plasmodesmata closure and may do orders of physiological events.

(Other comments)
1. The induced callose deposition at plasmodesmata triggers plasmodesmata closure, leading to decreased symplastic flux and SA increase. However, is it possible that callose

deposition itself triggers the SA independent of plasmodesmata closure? It is probably impossible to untangle these by experiments, but the authors can discuss this possibility.
2. The authors showed that Pst DC3000 hrcC infection triggers sustained callose deposition over 3 days. Is this also associated with actual sustained plasmodesmata closure? I think that the authors can easily show this.
3. I do not recommend green-red type heatmaps as color-blinded people cannot distinguish these two colors. A recommendation is blue-yellow.
4. Fig2A. It makes sense to combine heatmaps of different time points so that temporal dynamics of transcriptional reprogramming can be seen.
5. Fig2C: Different genotypes for different genes? It is unclear from the legend.
6. FigS8: It would inform readers better if the authors show expression of those genes under typical ER stress response (from a public data set?).

(Minor comments)
- FigS1B: the label "estradiol + 9" should be corrected.
- In line 381, the sentence needs more reference figures.
- In FigS12, It would be preferable to show statistical significance.

Referee #2:

In this study, the authors specifically addressed how the closure of plasmodesmata (PD) contributes to plant immunity, distinct from other defense responses in pattern-triggered immunity (PTI). They utilized icals3m and the C-terminus of PDLP1 to promote callose deposition at the PD. The results indicate that PD closure is sufficient to induce stress-responsive genes, SA production and bacterial resistance. Additionally, it leads to sugar and starch accumulation, decreased leaf growth and enhanced susceptibility to the necrotrophic pathogen Botrytis cinerea. This is a compelling investigation that provides novel and crucial insight into the role of PD closure in plant immunity and related processes. Furthermore, since PD plays a critical role in cell-to-cell signaling, this work has the potential to attract a readership well beyond its immediate field of research. However, while the hypotheses presented are intriguing, there is a lack of solid evidence connecting PD closure to the observed phenotypic effects. The mechanisms linking these phenomena have not been genetically validated or refuted. Therefore, this work requires attention to the following concerns to ensure they are addressed adequately.

Major concerns:
1) The developed estradiol-inducible callose-PD closure systems were only tested at 24 h

post-induction. It is essential to characterize these systems in a detailed time-course study. Specifically, how quickly can callose deposition and PD closure be induced after stimulating the systems? Since "PD closure is an early reaction" as stated in L349, the authors should provide evidence or at least test how effectively the system can trace immediate effects following PD closure.
Additionally, it is important to determine whether the induction of PD closure reaches saturation at 24 h post-stimulation and if these levels are maintained consistently thereafter.
This information would aid in better interpreting the results in Fig 2.
Regarding the testing of flg22 responses in Fig 3E, it is important to clarify the rationale for assessing these responses at 72 h post-stimulation.
Furthermore, when evaluating the impacts of PD closure on various phenotypic outputs, it would be beneficial to correlate the states or levels of PD closure with the magnitude or duration of the outputs in independent transgenic lines or with different doses of estradiol.
Lastly, I am curious whether the observed heterogeneity in cell-to-cell connectivity e.g. in LexA::PD-Plug plants arises from tissue or cell type specificity. Addressing these concerns could significantly enhance the clarity and depth of the findings presented in the study.

2) The hypotheses in L283-289 need to be tested at least genetically. It is important to test whether and if so by what mechanisms SA induction and signaling contributes to to enhanced bacterial (biotrophy) resistance in LexA::icals3m and LexA::PD-Plug. Is this also through previously defined key regulators of SA-based defense, such as SID2 or NPR1? The significance of other components such as SWEET15 (L399-402) proposed will also need to be tested under the authors' conditions. The authors have not sufficiently exploited their systems and unique opportunities to clearly define key mechanistic links between PD closure and subsequent defense-related outputs.

Detailed points:
In L46-48, the basis of the statement (references) needs to be provided.

In L113-115, "5' and 3' ends" are used for nucleic acids. Replace them with N and C termini.

In Fig 1B, why do even mock (DMSO) samples look so distinct between PD-Plug and icals3m?

In response to Pst DC3000 hrcC, the time required for detectable callose deposition may include the time required for bacterial propagation. I agree with the conclusion that callose deposition can be sustained, but if bacterial inoculation is intensified e.g. by increasing

bacterial dose, dose it induce callose deposition faster?

L266-268, what is the subject of "not the cause of the increased resistance"? The sentence seems to require correction.

L293, "phenotypically" sounds odd, as pathogen resistance tested above is also phenotypic effects.

L342-345, based on the negative data, one cannot make such strong statement. It is still possible that the extent of PD closure is not sufficient in these transgenic lines to significantly cause the tested changes.

The authors should discuss the possible linkage between sugar accumulation and SA-based defense, by citing previous studies on this topic, e.g. Yamada and Mine, 2024 Science Advances and related references therein.

L408-409, this seems an over-statement. In my view, it remains to be tested whether the observed heterogeneity is inherent to PD aperture regulation or is attributable to the system used.

Referee #3:

This study investigates the impact of plasmodesmata closure on plant physiological responses and disease resistance to phytopathogens. The authors developed two inducible plant transgenic lines, by which plasmodesmata closure can be triggered through chemical treatment. Interestingly, the close of plasmodesmata initiates stress transcriptional responses, accumulates salicylic acid, alters pathogen resistance of plants and leads to sugar accumulation in the source leaf. The tools created through this study are valuable for investigating the role of plasmodesmata in various plant processes. The experiments were clearly described and results are well presented. The manuscript is logical and organized.

My specific comments:
1. The SA-related gene expression changes after estradiol treatment is fairly late (e.g., after 48h). In addition, other immune responses such as MAPK phosphorylation and ROS do not show a consistent change with SA following induced plasmodesmata closure. These seem

to support that SA induction can be an indirect effect of plasmodesmata closure. Given that plasmodesmata presumably facilitate the transport of many molecules between cells, a blockage of plasmodesmata could impact a variety of cellular processes. It's therefore not surprising that plasmodesmata closure would eventually lead to a stress response in the plant tissue. If possible, it would be much better to define a specific immune molecule/response that is transported/mediated by plasmodesmata between plant cells and investigate how plasmodesmata regulates this specific process.

2. Plasmodesmata closure makes cells in an isolated status and prevents cellular communication. Does this somehow affect systemic defense responses? For example, if an ETI-eliciting strain is inoculated on a primary leaf, how might plasmodesmata closure influence defense responses or SAR in the system leaf? The authors should design experiments to explore potential additional roles of plasmodesmata closure in plant disease processes.

3. Figure 4 presents interesting results, but lacks a clear connection to the plant defense outcomes discussed in the study. I suggest the authors either place this within a broader context of plant physiology or forge a stronger link to the plant defense aspects of the study.

We previously submitted our publication “Plasmodesmal closure elicits stress responses” (EMBOJ-2024-118897) to EMBO Journal, and the editor suggested that the study would be welcome at EMBO Reports with the following revisions:

1. Provide further insight into the roles of SA and JA in resistance against P. syringae and susceptibility to B. cinerea, as suggested by Referee 1, point 1. If possible, include genetic evidence for the involvement of previously defined key regulators of SA-based defense and/or tone down the conclusion in L283-289 (Referee 2, point 2).

2. Testing the involvement of sugar accumulation in pathogen resistance would certainly strengthen the study, in case mutations to block the sugar increase are available, but these experiments are not mandatory for potential publication in EMBO Reports.

3. Provide a time course/kinetics of PD closure and test saturation (Referee 2, point 1).

4. Address all other comments and concerns from Referee 1 and Referee 2 Address the concerns from Referee 3 in a point-by-point response and by textual changes, i.e., in an extended discussion of the limitations of the current study and potential future lines of research.'

We have performed a number of experiments to address these points to improve the manuscript story, as summarised:

To address 1, we took multiple approaches:

i) We quantified JA and found that when SA increases, JA is unchanged. We also note that despite undetectable changes to JA upon plasmodesmal closure, our transcriptomic analysis suggesting that JA responses, alongside SA responses, increase when plasmodesmata close. Therefore, our data does not support the argument that defence hormone antagonism mediates altered pathogen susceptibility.

ii) We generated a NahG × LexA::icals3m cross. As NahG is well-established to degrade salicylic acid these lines will be depleted in SA and can therefore be used to disconnect SA accumulation from sugar accumulation. The NahG × LexA::icals3m cross shows growth inhibition and senescence comparable to LexA::icals3m when plasmodesmal closure is induced suggesting SA is not the primary cause of growth inhibition or senescence (new Fig 4C). Furthermore, the NahG × LexA::icals3m cross shows enhanced resistance to *P. syringae* when plasmodesmal closure is induced, indicating SA is not the main determinant for enhanced resistance induced by plasmodesmal closure.

To address point 2 in part, we shaded the plants to reduce photosynthesis and sugar accumulation and examined the effect on growth and senescence. In shade conditions, plant growth was reduced in all genotypes, but plasmodesmal closure in both LexA::icals3m and NahG × LexA::icals3m induced further growth inhibition. Senescence was evident but not as pronounced as that observed in the light condition, again suggesting that sugar accumulation is neither the primary cause of growth inhibition or senescence.

To address point 3, we performed a time-course of plasmodesmal callose deposition in LexA::Plug and LexA::icals3m. This data in the new Supplemental Figure 4.

Our responses relating to point 4 are itemised below. Our responses (italics) follow the reviewers’ comment in normal font.

Referee #1:

This manuscript addresses the question of what kind of physiological responses happen in response to artificial plasmodesmata closure in order to understand the role of plasmodesmata closure in plant immunity.

The authors used approaches where transgenic plants induce callose deposition at plasmodesmata leading to plasmodesmata closure upon estradiol treatment. They found that salicylic acid (SA) and sugar accumulation increase associated with increased resistance against the biotrophic bacterial pathogen Pseudomonas syringae and with increased susceptibility to the necrotrophic fungal pathogen Botrytis cinerea.

Plasmodesmata close during pathogen infection but its role remains uncertain. The authors tackled the significant question by using a cool approach and revealed physiological responses. While their findings are novel and significant even though it has been known that constitutive plasmodesmata closure leads to SA increase, I feel that the story is incomplete for this journal. I find two major gaps in this study that need to be deciphered in the role of plasmodesmata closure in plant immunity. The first point is the mechanism by which plants actually trigger physiological responses such as SA and sugar increase upon plasmodesmata closure. The second point is what physiological responses are relevant for pathogen resistance. While I think that the first point might be beyond the scope of this study, the second point can be experimentally addressed.

(Major comments)

It makes sense that increased SA upon plasmodesmata closure causes increased resistance against P. syringae, which is sensitive to SA-mediated immunity. In addition, increased SA can lead to decreased jasmonate (JA) signaling due to SA-JA crosstalk, which likely causes increased susceptibility to B. cinerea, which is sensitive to JA-mediated immunity. The authors can test these hypotheses by generating transgenic plants in SA and JA-deficient mutant backgrounds.

If possible (maybe unrealistic), it would be great if the authors can also block sugar increase upon plasmodesmata closure by introducing some mutation. Then, they can measure SA and sugar in those conditions and perform pathogen assays. These will address the cause of changed pathogen resistance upon plasmodesmata closure and may do orders of physiological events.

The reviewer is correct to suggest that altered SA-JA signalling might be the cause of increased Botrytis susceptibility in induced LexA::icals3m, based on many previous observations in the literature. To further investigate whether increased SA induced by plasmodesmal closure impacted baseline JA levels, we quantified JA in estradiol treated Col-0, LexA::Plug and LexA::icals3m at the same time point we identified SA accumulation (i.e., 72h post plasmodesmal closure; L238-243). In no genetic background did estradiol cause JA levels to change suggesting that baseline JA is not responsible for the Botrytis susceptibility observed (new Supplemental Figure S14). We also noted that genes associated with JA response are induced alongside genes associated with SA response when plasmodesmal closure is induced (Fig. 2b), suggesting the possibility that JA increases occur but are below the detectable levels using whole tissue extracts and mass spectrometry (specified in L240-243). Neither of these results support defence hormone antagonsim as a reason for the phenotypes observed.

We generated a LexA::icals3m × 35S::NahG line (NahG × icals3m) in which we expect SA to be degraded when plasmodesmata close. We first used this to explore whether SA or sugars induce the dwarfing and senescence phenotypes that occur when plasmodesmata are closed (L353-365). This data is added to Fig. 4c and a new Supplemental Figure S21. Plant growth assays with the NahG × icals3m cross show impaired growth and leaf senescence when plasmodesmata are closed, suggesting SA is not the primary factor that causes these responses.

To reduce sugar content, we shaded the plants (L338-350). In these experiments, LexA::icals3m and NahG × icals3m both showed impaired growth to similar degrees when plasmodesmata are closed. Senescence was also induced in both lines, although to a lesser degree to the control light conditions, suggesting that sugar accumulation might contribute to this phenotype.

Lastly, we examined the contribution of SA to plasmodesmal closure enhanced resistance to P. syringae by performing pathoassays on the NahG × icals3m cross alongside LexA::icals3m (L366-373). This has been added to Fig. 4d. Uninduced NahG × icals3m had a higher susceptibility to P. syringae in comparison to uninduced LexA::icals3m as expected, but inducing plasmodesmal closure in both lines enhanced resistance indicating SA accumulation is not the primary cause of the increased resistance induced by plasmodesmal closure.

(Other comments)

1. The induced callose deposition at plasmodesmata triggers plasmodesmata closure, leading to decreased symplastic flux and SA increase. However, is it possible that callose deposition itself triggers the SA independent of plasmodesmata closure? It is probably impossible to untangle these by experiments, but the authors can discuss this possibility.

We observed callose deposition almost exclusively, and certainly primarily, at plasmodesmata when estradiol was the only elicitor present, as seen in Supplemental Fig. S2. Therefore, as we did not observe callose deposited elsewhere as a consistent and dominant response, we have not discussed the possibility that it is the cause of the measured phenotypes.

2. The authors showed that Pst DC3000 hrcC infection triggers sustained callose deposition over 3 days. Is this also associated with actual sustained plasmodesmata closure? I think that the authors can easily show this.

We have shown in the experiments here, as well as in our previous publications (Faulkner et al 2013, Xu et al., 2017, Cheval et al. 2020, Bellandi et al 2022, Tee et al 2023) that callose deposition at plasmodesmata is correlated with functional plasmodesmata closure. Indeed, genetic studies and electron microscopy (e.g. Vaten et al. 2011; Benitez-Alfonso et al. 2013) indicate that callose is the material that induces the physical plasmodesmal closure response. Therefore, we think it is reasonable to conclude that if there is an increase in callose at plasmodesmata, there is also an impact on plasmodesmal function.

3. I do not recommend green-red type heatmaps as color-blinded people cannot distinguish these two colors. A recommendation is blue-yellow.

We agree that red-green is not suitable for colour blind readers. However, our heat map is magenta-green for this reason.

4. Fig2A. It makes sense to combine heatmaps of different time points so that temporal dynamics of transcriptional reprogramming can be seen.

Our rationale for separating the visualisation of the data into timepoints was that our analysis was done within these timepoints based on the results of our PCA of the whole dataset (see Supplemental Fig. S6). Therefore, this representation best fits our analysis. The DEG sets in the heatmaps for each timepoint are different and therefore presentation of the temporal dynamics requires a different analysis or manual selection of all genes contained in the different datasets. We note that for the SA-associated genes in Fig. 2c, we have manually extracted the data for specific genes and present the data in a way that displays the temporal dynamics. Our data will be publicly available, and anyone can perform whatever analysis is of relevance to their interests.

5. Fig2C: Different genotypes for different genes? It is unclear from the legend.

The data is pooled for the different genotypes to represent plasmodesmal closure as a phenomenon, as described in L213-223.

6. FigS8: It would inform readers better if the authors show expression of those genes under typical ER stress response (from a public data set?).

Readers are referred to a paper that identifies ER stress genes. Every experimental design is different and therefore reconciling our data with an independent dataset is unlikely to allow clear comparison between our data and an ER stress response.

(Minor comments)

- FigS1B: the label "estradiol + 9" should be corrected.

We have removed the 9h and 90 day panels

- In line 381, the sentence needs more reference figures.

In line 399-401 we have added a reference to Fig. 2d, Fig. 4c and Fig. 1c to justify the comment relating to the greater degree of plasmodesmal closure and the associated SA and sugar quantifications.

- In FigS12, It would be preferable to show statistical significance.

The statistical significance is shown in Fig. 3b (specifics in L1183-1187), this figure simply displays the data from individual repeats and is specified in the methods (L565-569) as what is defined as a single biological replicate (i.e., each plate).

Referee #2:

In this study, the authors specifically addressed how the closure of plasmodesmata (PD) contributes to plant immunity, distinct from other defense responses in pattern-triggered immunity (PTI). They utilized icals3m and the C-terminus of PDLP1 to promote callose deposition at the PD. The results indicate that PD closure is sufficient to induce stress-responsive genes, SA production and bacterial resistance. Additionally, it leads to sugar and starch accumulation, decreased leaf growth and enhanced susceptibility to the necrotrophic pathogen Botrytis cinerea. This is a compelling investigation that provides novel and crucial insight into the role of PD closure in plant immunity and related processes. Furthermore, since PD plays a critical role in cell-to-cell signaling, this work has the potential to attract a readership well beyond its immediate field of research. However, while the hypotheses presented are intriguing, there is a lack of solid evidence connecting PD closure to the observed phenotypic effects. The mechanisms linking these phenomena have not been genetically validated or refuted. Therefore, this work requires attention to the following concerns to ensure they are addressed adequately.

Major concerns:

1) The developed estradiol-inducible callose-PD closure systems were only tested at 24 h post-induction. It is essential to characterize these systems in a detailed time-course study. Specifically, how quickly can callose deposition and PD closure be induced after stimulating the systems? Since "PD closure is an early reaction" as stated in L349, the authors should provide evidence or at least test how effectively the system can trace immediate effects following PD closure.

Additionally, it is important to determine whether the induction of PD closure reaches saturation at 24 h post-stimulation and if these levels are maintained consistently thereafter. This information would aid in better interpreting the results in Fig 2. Regarding the testing of flg22 responses in Fig 3E, it is important to clarify the rationale for assessing these responses at 72 h post-stimulation.

Furthermore, when evaluating the impacts of PD closure on various phenotypic outputs, it would be beneficial to correlate the states or levels of PD closure with the magnitude or duration of the outputs in independent transgenic lines or with different doses of estradiol. Lastly, I am curious whether the observed heterogeneity in cell-to-cell connectivity e.g. in LexA::PD-Plug plants arises from tissue or cell type specificity. Addressing these concerns could significantly enhance the clarity and depth of the findings presented in the study.

Our statement of 'PD closure is an early reaction' in L349 is provided for the context of how plasmodesmal closure is initiated in response to stress elicitors, which activate specific receptor complexes and pathways already present to stimulate callose deposition via the callose synthases. This outlines the timing of when plasmodesmal closure occurs in an immune context. Our aim was to emphasise that disentangling the impact of plasmodesmal closure from other immune responses is complex (L375-387).

We acknowledge that this rapid induction is different to our system presented here, where we specifically first transcriptionally activate constructs, which leads to the translation of proteins

capable of producing callose independent of such stress stimuli. To illustrate the dynamics of our inducible system, in parallel to the time-course expression analysis of the transgene expression over time (Supplemental Fig. S9), we have additionally performed a time-course analysis of plasmodesmal closure via plasmodesmal callose deposition in both LexA::icals3m and LexA::PD-Plug (L147-151). This data is presented in a new Supplemental Fig. S4, and shows that in both lines, plasmodesmal callose is significantly induced 24h post estradiol treatment and is maintained at 48h and 72h. Having determined that 72h post infection with Pst hrcC^{-} that plasmodesmal closure is evident (Supplemental Fig. S5), we reason that this is a physiologically relevant timepoint.

Regarding the possibility that heterogeneity is cell-type specific: we only assay GFP flux between epidermal pavement cells, so the heterogeneity we observe is not a cell-type or tissue type specific factor. At least for the LexA::PD-Plug line, we specify that despite its homogenous expression (see Supplemental Fig. S1 as indicated by mCherry expression, specified in L441-442), its variability in GFP mobility resembles heterogeneous physiological datasets (Supplemental Fig. S3).

2) The hypotheses in L283-289 need to be tested at least genetically. It is important to test whether and if so by what mechanisms SA induction and signaling contributes to to enhanced bacterial (biotrophy) resistance in LexA::icals3m and LexA::PD-Plug. Is this also through previously defined key regulators of SA-based defense, such as SID2 or NPR1?

To address the role of SA and SA signalling in the phenotypes associated with plasmodesmal closure, we made a NahG × LexA::icals3m cross. We observed that this line, like LexA::icals3m, exhibits enhanced resistance to P. syringae when plasmodesmal closure is induced, indicating that SA and downstream signalling is not the cause of induced resistance to a biotroph.

The significance of other components such as SWEET15 (L399-402) proposed will also need to be tested under the authors' conditions. The authors have not sufficiently exploited their systems and unique opportunities to clearly define key mechanistic links between PD closure and subsequent defense-related outputs.

First, we think that the reviewer's suggestion that we have not sufficiently exploited our system is unreasonable. We discuss SWEET15 in the context of SA and senescence, but considering its increased expression of this transporter does not counteract the intracellular sugar accumulation, we did not consider that it warranted further experimental investigation at this stage.

Detailed points:

In L46-48, the basis of the statement (references) needs to be provided.

We have added example references Lee et al. 2011 and Faulkner et al. 2013.

In L113-115, "5' and 3' ends" are used for nucleic acids. Replace them with N and C termini.

We have edited this section and no longer make reference to 5' or 3'.

In Fig 1B, why do even mock (DMSO) samples look so distinct between PD-Plug and icals3m?

Pavement cell bombardments produce data with high variance (see Fig S3) - each image does look different. Our data will be deposited in the bioimage archive to be evaluated by others.

In response to Pst DC3000 hrcC, the time required for detectable callose deposition may include the time required for bacterial propagation. I agree with the conclusion that callose deposition can be sustained, but if bacterial inoculation is intensified e.g. by increasing bacterial dose, dose it induce callose deposition faster?

Plasmodesmal closure occurs in response to flg22, and so it does not rely on bacterial propagation in the first instance. It certainly might be enhanced or hastened by both increased bacteria delivering increased flg22, which then activates more receptor, as well as by increased bacterial propagation that activates further responses that have a secondary effect on plasmodesmata. We appreciate the consideration and discussion, but this is far beyond the scope of our study.

L266-268, what is the subject of "not the cause of the increased resistance"? The sentence seems to require correction.

We have changed this sentence to read "To determine if plasmodesmal closure in our transgenic lines influences other PAMP responses" (L266-268).

L293, "phenotypically" sounds odd, as pathogen resistance tested above is also phenotypic effects.

We removed this sentence

L342-345, based on the negative data, one cannot make such strong statement. It is still possible that the extent of PD closure is not sufficient in these transgenic lines to significantly cause the tested changes.

I recognise why the reviewer feels that we have drawn a strong conclusion, but we aimed to simply comment that we did not see a change in expression of sugar transporters in our transcriptomic datasets. To emphasise this, we have rephrased the sentence (L334-337):

"These results indicate that while plasmodesmal closure induces detectable starch and soluble sugar accumulation, we were unable to detect a perturbation of photosynthesis or increased expression of sugar transporters."

The authors should discuss the possible linkage between sugar accumulation and SA-based defense, by citing previous studies on this topic, e.g. Yamada and Mine, 2024 Science Advances and related references therein.

Yamada and Mine study the effect of externally applied, non-metabolically active sugar. They also studied vastly different outputs other than SA accumulation. It is difficult to draw direct parallels between their study and ours, but we have made a note that they showed sugars can enhance immune signalling (L415-417).

L408-409, this seems an over-statement. In my view, it remains to be tested whether the observed heterogeneity is inherent to PD aperture regulation or is attributable to the system used.

We respectfully disagree with the reviewer. Both microprojectile bombardment and low OD agroinfiltration of fluorophores to observe cell-to-cell connectivity reveal heterogeneous degrees of plasmodesmal connectivity between cells (Crawford and Zambryski, 2001; Ohtsu et al 2024; Johnston and Faulkner, 2020; Cheval et al., 2020 plus more). Further, different levels of variance in the data (heterogeneity) are evident on a genotype specific basis and so we have no evidence to indicate that this is a technical factor rather than a biological one. Single cell sequencing demonstrates that cells within a tissue have different response profiles (e.g. Nobori et al., 2025) allowing for heterogeneity in connectivity is a reasonable inference from our data. Of course, every experimental system has its limitations, but we strongly consider that there is better evidence for cellular variation than technical variation. Further, our statement is that our data 'suggests' heterogeneity - we think this is entirely reasonable.

Referee #3:

This study investigates the impact of plasmodesmata closure on plant physiological responses and disease resistance to phytopathogens. The authors developed two inducible plant transgenic lines, by which plasmodesmata closure can be triggered through chemical treatment. Interestingly, the close of plasmodesmata initiates stress transcriptional responses, accumulates salicylic acid, alters pathogen resistance of plants and leads to sugar accumulation in the source leaf. The tools created through this study are valuable for investigating the role of plasmodesmata in various plant processes. The experiments were clearly described and results are well presented. The manuscript is logical and organized.

My specific comments:

1. The SA-related gene expression changes after estradiol treatment is fairly late (e.g., after 48h). In addition, other immune responses such as MAPK phosphorylation and ROS do not show a consistent change with SA following induced plasmodesmata closure. These seem to support that SA induction can be an indirect effect of plasmodesmata closure. Given that plasmodesmata presumably facilitate the transport of many molecules between cells, a blockage of plasmodesmata could impact a variety of cellular processes. It's therefore not surprising that plasmodesmata closure would eventually lead to a stress response in the plant tissue. If possible, it would be much better to define a specific immune molecule/response that is transported/mediated by plasmodesmata between plant cells and investigate how plasmodesmata regulates this specific process.

Identifying immune molecules/response that are transported by plasmodesmata was the aim of many research lines in the lab, run over 8 years with vast investments of money and people time. Unfortunately, despite using an expansive suite of plasmodesmal mutants and immune contexts, these studies have proven complex and while revealing several plasmodesmata-dependent aspects of response, did not reveal specific immune modules/molecules. This is why we ended up taking the approach that we have here.

Here, we have shown that plasmodesmal closure induces SA and sugar accumulation. By systematically removing them from the equation, we observed that plasmodesmal closure still induces detrimental phenotypes. Therefore, there are yet to be discovered processes that are impacted by plasmodesmal closure, and illustrates the complexity of the system. Multiple approaches and avenues of research will be required to map the full range of responses influenced by plasmodesmal closure.

2. Plasmodesmata closure makes cells in an isolated status and prevents cellular communication. Does this somehow affect systemic defense responses? For example, if an ETI-eliciting strain is inoculated on a primary leaf, how might plasmodesmata closure influence defense responses or SAR in the system leaf? The authors should design experiments to explore potential additional roles of plasmodesmata closure in plant disease processes.

We agree that the effects of plasmodesmal closure on SAR are interesting. However, our focus here was on local responses since plasmodesmata close in cells that perceive immune elicitors. To this effect, we have specified 'local defense responses' at the beginning of our

Discussion (L375), and added an extra sentence acknowledging how our tools could be used to explore SAR in the future (L412-414).

3. Figure 4 presents interesting results, but lacks a clear connection to the plant defense outcomes discussed in the study. I suggest the authors either place this within a broader context of plant physiology or forge a stronger link to the plant defense aspects of the study.

Figure 4 now contains more direct data relating to SA accumulation and whether this is a primary regulator of plasmodesmal-closure induced resistance. Further, we have discussed the role of plasmodesmal closure in physiology relating to photosynthesis and carbon accumulation.

Dear Prof. Faulkner

Thank you for the submission of your revised manuscript to EMBO reports. We have now received the full set of referee reports that is copied below.

As you will see, all referees are very positive about the study and support publication after a few remaining concerns are addressed.

Please address these remaining concerns from referee #1 and #3 and please provide a point-by-point response to these.

From the editorial side, there are also a few things that we need before we can proceed with the official acceptance of your study.

1) Please provide a .docx formatted version of the manuscript text (including legends for main figures, EV figures and tables). Please make sure that the changes are highlighted to be clearly visible. The table follows the figure legends.

2) Please remove the figures from the manuscript and upload them as individual production quality figure files as .eps, .tif, .jpg (one file per figure). See hhttps://media.springernature.com/original/springer-cms/rest/v1/content/27825798/data/v1 for more info on how to prepare your figures.

3) Your manuscript will be published as Scientific Report, which requires a combined Results and Discussion section.

4) All Materials and Methods need to be described in the main text using our 'Structured Methods' format, which is required for all research articles. According to this format, the Methods section includes a Reagents and Tools Table (listing key reagents, experimental models, software and relevant equipment and including their sources and relevant identifiers) followed by a Methods and Protocols section describing the methods using a step-by-step protocol format. The aim is to facilitate adoption of the methodologies across labs. More information on how to adhere to this format as well as a downloadable template (.docx) for the Reagents and Tools Table can be found in our author guidelines.
See https://link.springer.com/journal/44319/submission-guidelines

5) Please insert a link in the Data Availability section that directly resolves to the dataset GSE248301.
The Data Availability section currently states:
"Raw counts for bombardment data generated for this study (Fig. 1C and Supplemental Fig. S3) is available in Supplemental Data Set 1. Additional raw counts for bombardment data for Supplemental Fig. S3 are sourced from Cheval et al. 2020 and Tee et al. 2023b. RNAseq data has been deposited in the Gene Expression Omnibus under the accession code GSE248301, with further analysis included in Supplemental Data Sets as specified in-text."
This section should only refer to datasets deposited in public repositories. Therefore, the description of bombardment data, which is part of the manuscript itself should be added at another appropriate section in the manuscript. Please also note that the nomenclature "Supplemental" needs to be changed (see point 15).

6) It seems that you reused datasets generated by Cheval et al 2020 and Tee et al 2023b. If so, then please add data citations. You need to cite the paper that published the datasets and in addition, the dataset itself.
Data citations in the article text are distinct from normal bibliographical citations and should directly link to the database records from which the data can be accessed. In the main text, data citations are formatted as follows: "Data ref: Smith et al, 2001" or "Data ref: NCBI Sequence Read Archive PRJNA342805, 2017". In the Reference list, data citations must be labeled with " [DATASET]". A data reference must provide the database name, accession number/identifiers and a resolvable link to the landing page from which the data can be accessed at the end of the reference.
https://link.springer.com/journal/44319/submission-guidelines#cms-Reference-guidelines

7) The references have DOIs, which should only be used for preprints and datasets that have not been published yet. Please refer to our guide to authors regarding the correct citation format.

8) The Disclosure and competing interests statement needs to be placed after Acknowledgments.

9) Please provide the corresponding author's email address on the title page.

19) Please provide a callout for Dataset 10.

11) Regarding the Author Contributions, we now use CRediT to specify the contributions of each author in the journal submission system. Therefore, please remove the Author Contributions from the manuscript file and make sure that the author

contributions in our online manuscript tracking system are correct and up-to-date. The information you specified in the system will be automatically retrieved and typeset into the article. You can enter additional information in the free text box provided, if you wish. See also our guide to authors https://www.embopress.org/page/journal/14693178/authorguide#authorshipguidelines.

12) Please provide a complete author checklist, which you can download from our author guidelines. Please insert information in the checklist that is also reflected in the manuscript. The completed author checklist will also be part of the RPF.

13) Please complete the funding information in the online submission system and please make sure that the information in the system and in the Acknowledgments is congruent.

14) You have 14 datasets provided as one Excel file. Please upload each as a separate dataset file and the nomenclature should be corrected to Dataset EV1-Dataset EV14 in all places (each source file, legend, title in the system, callouts in the manuscript). The dataset legends need to be removed from the manuscript and instead added as a separate tab 'Legend' to each dataset.

15) The uploaded PDF with Supplementary figures and table needs a title page with the listed figures and the table and their page numbers; the file should be titled Appendix and the correct nomenclature in the file and manuscript text should be Appendix Figure S1, etc. Appendix Table S1, etc. ("Supplemental" should not be used).

16) Please address the following comments in the figure legends:
- Please provide the exact p values in the legends of figures 1A, C; 2D; 3A, B, D, E; 4A-D (unless p<0.001)
- Please indicate the statistical test used for data analysis in the legends of figures 1A, C; 2B; 3D, E
- Please note that the box plots need to be defined in terms of minima, maxima, centre, bounds of box and whiskers, and percentile in the legends of figures 1A, C; 2D, 3A, B, D, E; 4A-D

17) Appendix Figure legends:
- Figure S1 needs scale bars.
- Figure S4, S5: please define the exact p-values (unless p<0.001).
- Figure S4, S5, S8, S9, S11, S12, S14, S15, S17: please define the blox in terms of minima, maxima, centre, bounds of box and whiskers, and percentile
- The blots shown in Figure S16 are very small and should be enlarged.

18) At EMBO Press we ask authors to provide source data for the main manuscript figures. You will receive a separate email with instructions for providing source data with your revised manuscript, including how to upload and organize the files.

19) Finally, EMBO Reports papers are accompanied online by
A) a short (1-2 sentences) summary of the findings and their significance,
B) 2-3 bullet points highlighting key results and
C) a schematic summary figure that provides a sketch of the major findings (not a data image).
Please provide the summary figure as a separate file in PNG or JPG format at a size of 550x300-600 pixels (width x height). Please note that the size is rather small and that text needs to be readable at the final size. Please send us this information along with the revised manuscript.

We would also welcome the submission of cover suggestions, or motifs to be used by our Graphics Illustrator in designing a cover.

As part of the EMBO publication's Transparent Editorial Process, EMBO reports publishes online a Review Process File (RPF) to accompany accepted manuscripts. This File will be published in conjunction with your paper and will include the referee reports, your point-by-point response and all pertinent correspondence relating to the manuscript.

You are able to opt out of this by letting the editorial office know (emboreports@embo.org). If you do opt out, the Review Process File link will point to the following statement: "No Review Process File is available with this article, as the authors have chosen not to make the review process public in this case."

I look forward to seeing a revised form of your manuscript when it is ready.

Kind regards,

Martina Rembold, PhD
Senior Editor
EMBO reports

=================

Referee #1:

This manuscript shows the physiological responses induced by artificial plasmodesmata closure to elucidate the role of this process in plant immunity.

The authors have made improvements and have largely addressed the concerns raised in the previous review. Although the underlying cause(s) of enhanced resistance against the bacterial pathogen following plasmodesmata closure remain unclear, the authors have thoroughly characterized the associated physiological responses. The established experimental system represents a valuable tool for further dissecting the role of plasmodesmata in plant immunity, both by the authors and by other research groups.

One point requires additional consideration. The authors used the NahG transgene to reduce salicylic acid (SA) content. Because transgenes can be subject to silencing, I recommend that the authors measure SA levels in the NahG × icals3m transgenic plants to confirm that SA is indeed reduced. This validation is necessary before concluding that SA is not required for the enhanced resistance induced by plasmodesmata closure.

Referee #2:

The revised manuscript is improved. I have no further comments.

Referee #3:

I am pleased that the authors have adequately addressed my previous concerns.

Minor suggestions:
L72: The meaning of "normal" is unclear. Should this be "canonical"?

L254: The authors should specify whether the inoculation method was spray-inoculation or infiltration.

L262-263: The English requires correction. The headline in L247-248 should be revised in accordance with the corrected version of this sentence.

L265-266: The authors should note the uncoupling between plasmodesmal closure and MAPK/ROS activation in response to chitin (as described in L71-74). In Fig. 3C, estradiol-treated PD-Plug appears to show a slight enhancement of MAPK activation; quantification of band intensities would help substantiate (or refine) their conclusions.

L269-270: Because the experiments used leaf discs from mature leaves, this should be explicitly stated in addition to the description "mature leaves," particularly when presented alongside "(intact) seedlings."

L281-283: The logical flow needs improvement. In its current form, the statements in L281-283 do not follow smoothly from the preceding results on flg22 responses and H_2DCF-DA ROS staining.

L285-287: The basis for this statement is unclear. If the authors wish to retain it, they should test PD-closure effects at different developmental stages.
The plants shown in Fig. S18 appear to exhibit a shade-avoidance phenotype. The light conditions seem suboptimal for growth, although growth retardation in LexA::icals3m should occur irrespective of light quality.

L371-373: The logic is not convincing. To support this conclusion, the authors would need to compare NahG versus NahG icals3m after estradiol treatment to determine whether icals3m enhances resistance in the NahG background.

L433-435 and elsewhere: Can PD closure truly "isolate" cells from their neighbors? The phrasing should be reconsidered or clarified.

Thank you for your attention to our manuscript and we are pleased that the assessments are predominantly positive. We have dealt with the requests for further information and formatting changes, as well as addressing the reviewers remaining concerns. We describe our actions/edits in blue text in the list that follows:

Requests from the Editor:

1) Please provide a .docx formatted version of the manuscript text (including legends for main figures, EV figures and tables). Please make sure that the changes are highlighted to be clearly visible. The table follows the figure legends.
A new section has been added with figure legends consisting of Main figure legends, EV figure legends, Table legend, followed by the table.

2) Please remove the figures from the manuscript and upload them as individual production quality figure files as .eps, .tif, .jpg (one file per figure). See https://media.springernature.com/original/springer-cms/rest/v1/content/27825798/data/v1 for more info on how to prepare your figures.
Figures have been removed from the main manuscript and has been processed accordingly.

3) Your manuscript will be published as Scientific Report, which requires a combined Results and Discussion section.
We have combined the Results and Discussion. This required significant removal of text, but we think we have done this without losing information or significance.

4) All Materials and Methods need to be described in the main text using our 'Structured Methods' format, which is required for all research articles. According to this format, the Methods section includes a Reagents and Tools Table (listing key reagents, experimental models, software and relevant equipment and including their sources and relevant identifiers) followed by a Methods and Protocols section describing the methods using a step-by-step protocol format. The aim is to facilitate adoption of the methodologies across labs. More information on how to adhere to this format as well as a downloadable template (.docx) for the Reagents and Tools Table can be found in our author guidelines. See https://link.springer.com/journal/44319/submission-guidelines
A Reagents and Tools table has been created and will be uploaded in the manuscript tracking system.

5) Please insert a link in the Data Availability section that directly resolves to the dataset GSE248301.
The Data Availability section currently states: "Raw counts for bombardment data generated for this study (Fig. 1C and Supplemental Fig. S3) is available in Supplemental Data Set 1. Additional raw counts for bombardment data for Supplemental Fig. S3 are sourced from Cheval et al. 2020 and Tee et al. 2023b. RNAseq data has been deposited in the Gene Expression Omnibus under the accession code GSE248301, with further analysis included in Supplemental Data Sets as specified in-text." This section should only refer to datasets deposited in public repositories. Therefore, the description of bombardment data, which is part of the manuscript itself should be added at another appropriate section in the manuscript. Please also note that the nomenclature "Supplemental" needs to be changed (see point 15).

The RNAseq dataset has been deposited at ArrayExpress BioStudies (accession E-MTAB-16776), a link has been directly added after the accession code at L705-706.

Reference to raw counts of bombardment data has been removed from this section and has been included as a source data file for Fig. 1C.

For all image data including the raw images for the bombardments, the text has been changed to the following: *The original images for callose staining for Fig. 1A, Fig. EV1 and Fig. EV3 and bombardment images for Fig. 1B and Fig. 1C is available at BioStudies, accessible with the accession number of S-BIAD3028 (https://www.ebi.ac.uk/biostudies/bioimages/studies/S-BIAD3028).*

Nomenclature has been changed accordingly to point 15.

6) It seems that you reused datasets generated by Cheval et al 2020 and Tee et al 2023b. If so, then please add data citations. You need to cite the paper that published the datasets and in addition, the dataset itself. Data citations in the article text are distinct from normal bibliographical citations and should directly link to the database records from which the data can be accessed. In the main text, data citations are formatted as follows: "Data ref: Smith et al, 2001" or "Data ref: NCBI Sequence Read Archive PRJNA342805, 2017". In the Reference list, data citations must be labeled with "[DATASET]". A data reference must provide the database name, accession number/identifiers and a resolvable link to the landing page from which the data can be accessed at the end of the reference. https://link.springer.com/journal/44319/submission-guidelines#cms-Reference-guidelines

Where we specify data obtained from GFP mobility assays, we have added Data ref: before the references: *"This is reminiscent of the data obtained from GFP mobility assays of leaves treated with elicitors such as chitin and flg22 (Data ref: Cheval et al. 2020; Tee et al. 2023b)."* We have also added this in the legend (now Fig. EV2) as per the following: *"Data represented taken from Data ref: Cheval et al. (2020) for Col-0 mock and Col-0 chitin, and Data ref: Tee et al. (2023b) for PDLP1OE."*

In the reference section, we have additionally added the following references:

Cheval C, Samwald S, Johnston MG, Keijzer J de, Breakspear A, Liu X, Bellandi A, Kadota Y, Zipfel C, Faulkner C (2020) Chitin perception in plasmodesmata characterizes submembrane immune-signaling specificity in plants. Proc Natl Acad Sci U S A 117: 9621–9629 [DATASET] https://www.pnas.org/doi/10.1073/pnas.1907799117#supplementary-materials

Tee EE, Johnston MG, Papp D, Faulkner C (2023b) A PDLP-NHL3 complex integrates plasmodesmal immune signaling cascades. Proc Natl Acad Sci U S A 120: e2216397120 [DATASET] https://www.pnas.org/doi/10.1073/pnas.2216397120#supplementary-materials

7) The references have DOIs, which should only be used for preprints and datasets that have not been published yet. Please refer to our guide to authors regarding the correct citation format.
All DOIs have been removed from the reference list

8) The Disclosure and competing interests statement needs to be placed after Acknowledgments.
This has been done.

9) Please provide the corresponding author's email address on the title page.
Email address has been provided next to corresponding author's name, L15.

19) Please provide a callout for Dataset 10.
Dataset 10 has been changed to Dataset EV9, and is now referred to at L339, and in Appendix file L157 and L164-165.

11) Regarding the Author Contributions, we now use CRediT to specify the contributions of each author in the journal submission system. Therefore, please remove the Author Contributions from the manuscript file and make sure that the author contributions in our online manuscript tracking system are correct and up-to-date. The information you specified in the system will be automatically retrieved and typeset into the article. You can enter additional information in the free text box provided, if you wish. See also our guide to authors https://www.embopress.org/page/journal/14693178/authorguide#authorshipguidelines.
This section has been removed in the main manuscript.

12) Please provide a complete author checklist, which you can download from our author guidelines. Please insert information in the checklist that is also reflected in the manuscript. The completed author checklist will also be part of the RPF.
An author checklist has been completed.

13) Please complete the funding information in the online submission system and please make sure that the information in the system and in the Acknowledgments is congruent.
This will be completed.

14) You have 14 datasets provided as one Excel file. Please upload each as a separate dataset file and the nomenclature should be corrected to Dataset EV1-Dataset EV14 in all places (each source file, legend, title in the system, callouts in the manuscript). The dataset legends need to be removed from the manuscript and instead added as a separate tab 'Legend' to each dataset.
All datasets have been separated with accordingly and updated to the correct nomenclature throughout. Legends have been removed from the manuscript and is present in a separate tab with each dataset labelled accordingly.

15) The uploaded PDF with Supplementary figures and table needs a title page with the listed figures and the table and their page numbers; the file should be titled Appendix and the correct nomenclature in the file and manuscript text should be Appendix Figure S1, etc. Appendix Table S1, etc. ("Supplemental" should not be used).
A new file has been made titled Appendix with all figures and table citations updated as Appendix in the main manuscript and in appendix file. A title page with the listed figures and table has also been made, with designated page numbers.

16) Please address the following comments in the figure legends:
- Please provide the exact p values in the legends of figures 1A, C; 2D; 3A, B, D, E; 4A-D (unless $p<0.001$)

- Please indicate the statistical test used for data analysis in the legends of figures 1A, C; 2B; 3D, E
- Please note that the box plots need to be defined in terms of minima, maxima, centre, bounds of box and whiskers, and percentile in the legends of figures 1A, C; 2D, 3A, B, D, E; 4A-D

For 1A, 1C, 3D and 3E; a bootstrap analysis was utilised to statistically test the differences. This approach relies on resampling distributions, and does not generate a static p-value; a p value showing p < value is the most accurate representation of this statistical test. Therefore, for the aforementioned figures where the bootstrap analysis is used, the p value has been left to reflect this.

For 2B, the following has been specified in the figure legend: *Top 15 defense GO terms enriched over time when plasmodesmata are closed, using a Fisher's exact test cut off of p < 0.05.*

For 2D, p value has been specified in the figure legend: *Significant differences between treatment within a genotype analyzed using Mann-Whitney test denoted by ns p=0.14, ** p=0.002, or *** p < 0.001.*

For 3A and B, specific posthoc p values has been put in a Dataset EV8, and referred to in the figure legend. For 4A-D, specific posthoc p values have been put in a Dataset EV8, and referred to in the figure legend.
We chose this approach because there are 15 p-values in these comparison and we consider that the information is more easily comprehended in this format.

The following sentence has been added to each the figure legend of Fig 1A, Fig 1C, Fig 2D. Fig 3A, Fig 3B, Fig 3D, Fig 3E, Fig4 A-D: "*The center line marks the median, the box indicates the upper and lower quartiles, and the whiskers show the minimum and maximum values within 1.5× interquartile range.*"

17) Appendix Figure legends: Figure S1 needs scale bars.
- Figure S4, S5: please define the exact p-values (unless p<0.001).
- Figure S4, S5, S8, S9, S11, S12, S14, S15, S17: please define the blox in terms of minima, maxima, centre, bounds of box and whiskers, and percentile
- The blots shown in Figure S16 are very small and should be enlarged.

For Figure S1, a scale bar has been added.

See above point 16) about bootstrap analysis and p-values regarding Figure S4 and S5.

The following sentence has been added to each figure legend of Fig S4 (now Fig S2), Fig S5 (now Figure EV3), Fig S8 (now Fig S5), Fig S9 (now Fig S6), Fig S11 (now Fig S8), Fig S12 (now Fig S9), Fig S14 (Fig S11), Fig S15 (now Fig S12) and Fig S17 (now Fig S14): "*The center line marks the median, the box indicates the upper and lower quartiles, and the whiskers show the minimum and maximum values within 1.5× interquartile range.*"

Figure S16 (now Fig S13) has been enlarged.

18) At EMBO Press we ask authors to provide source data for the main manuscript figures. You will receive a separate email with instructions for providing source data with your revised manuscript, including how to upload and organize the files.

Source data has been provided for all main manuscript figures and extended figures.

19) Finally, EMBO Reports papers are accompanied online by
A) a short (1-2 sentences) summary of the findings and their significance,
B) 2-3 bullet points highlighting key results and
C) a schematic summary figure that provides a sketch of the major findings (not a data image). Please provide the summary figure as a separate file in PNG or JPG format at a size of 550x300-600 pixels (width x height). Please note that the size is rather small and that text needs to be readable at the final size. Please send us this information along with the revised manuscript.
A synopsis section has been added at the beginning of the manuscript, L17-27.

=================
Requests from Reviewers

Referee #1:

This manuscript shows the physiological responses induced by artificial plasmodesmata closure to elucidate the role of this process in plant immunity.

The authors have made improvements and have largely addressed the concerns raised in the previous review. Although the underlying cause(s) of enhanced resistance against the bacterial pathogen following plasmodesmata closure remain unclear, the authors have thoroughly characterized the associated physiological responses. The established experimental system represents a valuable tool for further dissecting the role of plasmodesmata in plant immunity, both by the authors and by other research groups.

One point requires additional consideration. The authors used the NahG transgene to reduce salicylic acid (SA) content. Because transgenes can be subject to silencing, I recommend that the authors measure SA levels in the NahG × icals3m transgenic plants to confirm that SA is indeed reduced. This validation is necessary before concluding that SA is not required for the enhanced resistance induced by plasmodesmata closure

Acknowledging the reviewer's concern is gene silencing., we chose to directly measure NahG mRNA abundance. Thus, we performed a qPCR in the NahG × icals3m transgenic plants and found similar abundance of the *NahG* transcript in NahG and the NahG x LexA::icals3m cross – see L371, and new Appendix Fig. S17. Further, the NahG × icals3m treated with DMSO showed significantly increased bacterial growth of *Pst* DC3000 than all other conditions, including LexA::icals3m DMSO (i.e., non-induced icals3m), suggesting *NahG* expression and SA reduction causes to impaired resistance to *Pst* DC3000.

Referee #2:
The revised manuscript is improved. I have no further comments.

Referee #3:
I am pleased that the authors have adequately addressed my previous concerns.

Minor suggestions
L72: The meaning of "normal" is unclear. Should this be "canonical"?

We have replaced the word 'normal' to 'canonical'

L254: The authors should specify whether the inoculation method was spray-inoculation or infiltration.
We specified in the methods section in the *Pseudomonas syringae* infection assay that we infiltrated the inoculum (see L469).

L262-263: The English requires correction. The headline in L247-248 should be revised in accordance with the corrected version of this sentence.
We have re-written this sentence to: *"This indicates that plasmodesmal closure specifically does not lead to the enhancement of resistance in all infection contexts."* This reflects the headline, 'Plasmodesmal closure differentially impacts pathogen resistance and immune responses'.

L265-266: The authors should note the uncoupling between plasmodesmal closure and MAPK/ROS activation in response to chitin (as described in L71-74). In Fig. 3C, estradiol-treated PD-Plug appears to show a slight enhancement of MAPK activation; quantification of band intensities would help substantiate (or refine) their conclusions.

We have addressed the relationship (or lack of) between PD closure, susceptibility, MAPK and ROS responses in the conclusion of this section: "Increased resistance to *Pst* DC3000 <u>mediated by plasmodesmal closure</u> is not associated with enhanced MAPK activation or, given that our pathoassays were performed on mature leaves, changes in ROS burst responses." We are extremely close to the character limit and so we hope this satisfies the reviewer.

Given the variance we observe across experimental replication in diverse experiments, we did not assess the marginal variation in band intensity as robust or significant.

We have replaced the word 'magnitude' to 'activation' to make this statement clearer, indicating that there is no strong/obvious change of MAPK activation that plasmodesmal closure induces.

L269-270: Because the experiments used leaf discs from mature leaves, this should be explicitly stated in addition to the description "mature leaves," particularly when presented alongside "(intact) seedlings."
We have made the edits as per the following: *"When we assayed for flg22-triggered MAPK phosphorylation in **leaf discs of** mature leaves and **intact** seedlings of both lines following estradiol treatment…"*

L281-283: The logical flow needs improvement. In its current form, the statements in L281-283 do not follow smoothly from the preceding results on flg22 responses and H_2DCF-DA ROS staining.

In combining the results and discussion, and to make this clearer, we have separated out the conclusions. Now, our conclusion that the role of PD closure might differ with infection lifestyles immediately follows the presentation of the pathoassay data and the conclusion regarding the lack of relationship between PD closure, susceptibility to Pst DC3000 and MAPK and ROS responses follows this data.

L285-287: The basis for this statement is unclear. If the authors wish to retain it, they should test PD-closure effects at different developmental stages.

While we think that our statement here is justified because it arose from our tests of the effects of PD closure on ROS produced at different stages of development (seedlings v mature rosette leaves), in reducing the manuscript to 25000 characters, we have removed the statement.

The plants shown in Fig. S18 appear to exhibit a shade-avoidance phenotype. The light conditions seem suboptimal for growth, although growth retardation in LexA::icals3m should occur irrespective of light quality.

We note the reviewer's concern. However, as the reviewer notes this should not affect the growth retardation that we assay for.

L371-373: The logic is not convincing. To support this conclusion, the authors would need to compare NahG versus NahG icals3m after estradiol treatment to determine whether icals3m enhances resistance in the NahG background.

We respectfully disagree. At the request of the reviewers, this experiment was undertaken to determine whether the increase in resistance we observed when plasmodesmal closure is induced is caused by the accumulation of salicylic acid. Therefore, we designed this experiment to differentially vary salicylic acid accumulation when plasmodesmata were closed and assay for a difference. For this, the LexA::icals3m line must be the genetic background that remains constant and we crossed the NahG line with the LexA::icals3m, anticipating that the NahG transgene would impair SA accumulation when the icals3m transgene was induced. Then we tested Pst D3000 susceptibility in induced and uninduced icals3m and NahG × LexA::icals3m. These data and analysis allowed us to make the following observations:

1. SA accumulation enhances defence – LexA::icals3m uninduced is more resistant than NahG × LexA::icals3m uninduced (Note that the NahG × LexA::icals3m uninduced plants are akin to NahG plants, i.e. they have impaired SA degradation with no induction of plasmodesmal closure).
2. PD closure enhances defence – LexA::icals3m induced is more resistant than LexA::icals3m uninduced.
3. PD closure enhanced defence is not dependent on SA accumulation – NahG × LexA::icals3m induced is more resistant than NahG × LexA::icals3m uninduced
4. The combined effect of PD closure and SA degradation (NahG × LexA::icals3m induced) results in susceptibility comparable to plants in which plasmodesmal closure is not induced and SA accumulation remains unperturbed (LexA::icals3m uninduced)

L433-435 and elsewhere: Can PD closure truly "isolate" cells from their neighbors? The phrasing should be reconsidered or clarified.
Because of the re-working of the manuscript to combine the results and discussion, this is no longer an issue. Our use of the term isolation only now appears in our concluding paragraph in relation to our opinion that cellular isolation is a phenomenon that is important to understand.

1st Revision - Editorial Decision 13th Apr 2026

Prof. Christine Faulkner
John Innes Centre
Cell and Developmental Biology
United Kingdom

Dear Prof. Faulkner,

I am very pleased to accept your manuscript for publication in the next available issue of EMBO reports. Thank you for your contribution to our journal.

Your manuscript will be processed for publication by EMBO Press. It will be copy edited and you will receive page proofs prior to publication. Please note that you will be contacted by Springer Nature Author Services to complete licensing and payment information.

You may qualify for financial assistance for your publication charges - either via a Springer Nature fully open access agreement or an EMBO initiative. Check your eligibility: https://link.springer.com/journal/44319/how-to-publish-with-us

Should you be planning a Press Release on your article, please get in contact with embo_production@springernature.com as early as possible in order to coordinate publication and release dates.

If you have any questions, please do not hesitate to contact the Editorial Office. Thank you for your contribution to EMBO Reports.

Yours sincerely,

Martina Rembold, PhD
Senior Editor
EMBO reports

>>> Please note that it is EMBO Reports policy for the transcript of the editorial process (containing referee reports and your response letter) to be published as an online supplement to each paper. If you do NOT want this, you will need to inform the Editorial Office via email immediately. More information is available here: https://link.springer.com/partners/embo-press/editorial-policies#Peer%20review

EMBO Press Author Checklist

Corresponding Author Name: Christine Faulkner	
Journal Submitted to: EMBO Reports	
Manuscript Number: EMBOR-2025-63004-T	

USEFUL LINKS FOR COMPLETING THIS FORM
The EMBO Journal - Author Guidelines
EMBO Reports - Author Guidelines
Molecular Systems Biology - Author Guidelines
EMBO Molecular Medicine - Author Guidelines

Reporting Checklist for Life Science Articles (updated January
This checklist is adapted from Materials Design Analysis Reporting (MDAR) Checklist for Authors. MDAR establishes a minimum set of requirements in transparent reporting in the life sciences (see Statement of Task: 10.31222/osf.io/9sm4x). Please follow the journal's guidelines in preparing your
Please note that a copy of this checklist will be published alongside your article.

Abridged guidelines for figures
1. Data
The data shown in figures should satisfy the following conditions:
- the data were obtained and processed according to the field's best practice and are presented to reflect the results of the experiments in an accurate and unbiased manner.
- ideally, figure panels should include only measurements that are directly comparable to each other and obtained with the same assay.
- plots include clearly labeled error bars for independent experiments and sample sizes. Unless justified, error bars should not be shown for technical
- if n<5, the individual data points from each experiment should be plotted. Any statistical test employed should be justified.
- Source Data should be included to report the data underlying figures according to the guidelines set out in the authorship guidelines on Data

2. Captions
Each figure caption should contain the following information, for each panel where they are relevant:
- a specification of the experimental system investigated (eg cell line, species name).
- the assay(s) and method(s) used to carry out the reported observations and measurements.
- an explicit mention of the biological and chemical entity(ies) that are being measured.
- an explicit mention of the biological and chemical entity(ies) that are altered/varied/perturbed in a controlled manner.
- the exact sample size (n) for each experimental group/condition, given as a number, not a range;
- a description of the sample collection allowing the reader to understand whether the samples represent technical or biological replicates (including how many animals, litters, cultures, etc.).
- a statement of how many times the experiment shown was independently replicated in the laboratory.
- definitions of statistical methods and measures:
  - common tests, such as t-test (please specify whether paired vs. unpaired), simple χ2 tests, Wilcoxon and Mann-Whitney tests, can be unambiguously identified by name only, but more complex techniques should be described in the methods section;
  - are tests one-sided or two-sided?
  - are there adjustments for multiple comparisons?
  - exact statistical test results, e.g., P values = x but not P values < x;
  - definition of 'center values' as median or average;
  - definition of error bars as s.d. or s.e.m.

Please complete ALL of the questions below.
Select "Not Applicable" only when the requested information is not relevant for your study.

Materials

Newly Created Materials	Information included in the manuscript?	In which section is the information available? (Reagents and Tools Table, Materials and Methods, Figures, Data Availability Section)
New materials and reagents need to be available; do any restrictions apply?	Yes	Reagents and Tools Table, Methods

Antibodies	Information included in the manuscript?	In which section is the information available? (Reagents and Tools Table, Materials and Methods, Figures, Data Availability Section)
For **antibodies** provide the following information:<br>- Commercial antibodies: RRID (if possible) or supplier name, catalogue number and or/clone number<br>- Non-commercial: RRID or citation	Yes	Reagents and Tools Table, Methods

DNA and RNA sequences	Information included in the manuscript?	In which section is the information available? (Reagents and Tools Table, Materials and Methods, Figures, Data Availability Section)
**Short novel DNA or RNA including primers, probes**: provide the sequences.	Yes	Methods

Cell materials	Information included in the manuscript?	In which section is the information available? (Reagents and Tools Table, Materials and Methods, Figures, Data Availability Section)
**Cell lines:** Provide species information, strain. Provide accession number in repository **OR** supplier name, catalog number, clone number, and/**OR** RRID.	Not Applicable	
**Primary cultures:** Provide species, strain, sex of origin, genetic modification status.	Not Applicable	
Report if the cell lines were recently **authenticated** (e.g., by STR profiling) and tested for mycoplasma contamination.	Not Applicable	

Experimental animals	Information included in the manuscript?	In which section is the information available? (Reagents and Tools Table, Materials and Methods, Figures, Data Availability Section)
**Laboratory animals or Model organisms:** Provide species, strain, sex, age, genetic modification status. Provide accession number in repository **OR** supplier name, catalog number, clone number, **OR** RRID.	Not Applicable	
**Animal observed in or captured from the field:** Provide species, sex, and age where possible.	Not Applicable	
Please detail **housing and husbandry conditions**.	Not Applicable	

Plants and microbes	Information included in the manuscript?	In which section is the information available? (Reagents and Tools Table, Materials and Methods, Figures, Data Availability Section)
**Plants:** provide species and strain, ecotype and cultivar where relevant, unique accession number if available, and source (including location for collected wild specimens).	Yes	Regaents and Tools Table, Methods
**Microbes:** provide species and strain, unique accession number if available, and source.	Yes	Regaents and Tools Table, Methods

Human research participants	Information included in the manuscript?	In which section is the information available? (Reagents and Tools Table, Materials and Methods, Figures, Data Availability Section)
If collected and within the bounds of privacy constraints report on age, sex and gender or ethnicity for all study participants.	Not Applicable	

Core facilities	Information included in the manuscript?	In which section is the information available? (Reagents and Tools Table, Materials and Methods, Figures, Data Availability Section)
If your work benefited from core facilities, was their service mentioned in the acknowledgments section?	Not Applicable	

Design

Study protocol	Information included in the manuscript?	In which section is the information available? (Reagents and Tools Table, Materials and Methods, Figures, Data Availability Section)
If study protocol has been **pre-registered, provide DOI in the manuscript**. For clinical trials, provide the trial registration number **OR** cite DOI.	Not Applicable	
Report the **clinical trial registration number** (at ClinicalTrials.gov or equivalent), where applicable.	Not Applicable	

Laboratory protocol	Information included in the manuscript?	In which section is the information available? (Reagents and Tools Table, Materials and Methods, Figures, Data Availability Section)
Provide DOI OR other citation details if **external detailed step-by-step protocols** are available.	Yes	Methods

Experimental study design and statistics	Information included in the manuscript?	In which section is the information available? (Reagents and Tools Table, Materials and Methods, Figures, Data Availability Section)
Include a statement about **sample size** estimate even if no statistical methods were used.	Yes	Figures
Were any steps taken to minimize the effects of subjective bias when allocating animals/samples to treatment (e.g. **randomization procedure**)? If yes, have they been described?	Not Applicable	
Include a statement about **blinding** even if no blinding was done.	Not Applicable	
Describe **inclusion/exclusion criteria** if samples or animals were excluded from the analysis. Were the criteria pre-established?<br><br>If sample or data points were omitted from analysis, report if this was due to attrition or intentional exclusion and provide justification.	Not Applicable	
For every figure, are **statistical tests** justified as appropriate? Do the data meet the assumptions of the tests (e.g., normal distribution)? Describe any methods used to assess it. Is there an estimate of variation within each group of data? Is the variance similar between the groups that are being statistically compared?	Yes	Figures, Methods

Sample definition and in-laboratory replication	Information included in the manuscript?	In which section is the information available? (Reagents and Tools Table, Materials and Methods, Figures, Data Availability Section)
In the figure legends: state number of times the experiment was **replicated** in laboratory.	Yes	Figures, Methods
In the figure legends: define whether data describe **technical or biological replicates**.	Yes	Figures, Methods

Ethics

Ethics	Information included in the manuscript?	In which section is the information available? (Reagents and Tools Table, Materials and Methods, Figures, Data Availability Section)
Studies involving **human participants**: State details of **authority granting ethics approval** (IRB or equivalent committee(s), provide reference number for approval.	Not Applicable	
Studies involving **human participants**: Include a statement confirming that **informed consent** was obtained from all subjects and that the experiments conformed to the principles set out in the WMA Declaration of Helsinki and the Department of Health and Human Services Belmont Report.	Not Applicable	
Studies involving **human participants:** For publication of **patient photos**, include a statement confirming that consent to publish was obtained.	Not Applicable	
Studies involving experimental **animals**: State details of **authority granting ethics approval** (IRB or equivalent committee(s), provide reference number for approval. Include a statement of compliance with ethical regulations.	Not Applicable	
Studies involving **specimen and field samples:** State if relevant **permits** obtained, provide details of authority approving study; if none were required, explain why.	Not Applicable	

Dual Use Research of Concern (DURC)	Information included in the manuscript?	In which section is the information available? (Reagents and Tools Table, Materials and Methods, Figures, Data Availability Section)
Could your study fall under dual use research restrictions? Please check biosecurity documents and list of **select agents and toxins** (CDC): https://www.selectagents.gov/sat/list.htm	Not Applicable	
If you used a select agent, is the security level of the lab appropriate and reported in the manuscript?	Not Applicable	
If a study is subject to dual use research of concern regulations, is the name of the **authority granting approval and reference number** for the regulatory approval provided in the manuscript?	Not Applicable	

Reporting

The MDAR framework recommends adoption of discipline-specific guidelines, established and endorsed through community initiatives. Journals have their own policy about requiring specific guidelines and recommendations to complement MDAR.

Adherence to community standards	Information included in the manuscript?	In which section is the information available? (Reagents and Tools Table, Materials and Methods, Figures, Data Availability Section)
State if relevant guidelines or checklists (e.g., **ICMJE, MIBBI, ARRIVE, PRISMA**) have been followed or provided.	Not Applicable	
For **tumor marker prognostic studies,** we recommend that you follow the **REMARK** reporting guidelines (see link list at top right). See author guidelines, under 'Reporting Guidelines'. Please confirm you have followed these guidelines.	Not Applicable	
For **phase II and III randomized controlled trials**, please refer to the **CONSORT** flow diagram (see link list at top right) and submit the CONSORT checklist (see link list at top right) with your submission. See author guidelines, under 'Reporting Guidelines'. Please confirm you have submitted this list.	Not Applicable	

Data Availability

Data availability	Information included in the manuscript?	In which section is the information available? (Reagents and Tools Table, Materials and Methods, Figures, Data Availability Section)
Have **primary datasets** been deposited according to the journal's guidelines (see 'Data Deposition' section) and the respective accession numbers provided in the Data Availability Section?	Yes	Methods, Data Availability Section
Were **human clinical and genomic datasets** deposited in a public access-controlled repository in accordance to ethical obligations to the patients and to the applicable consent agreement?	Not Applicable	
Are **computational models** that are central and integral to a study available without restrictions in a machine-readable form? Were the relevant accession numbers or links provided?	Not Applicable	
If publicly available data were reused, provide the respective **data citations in the reference list**.	Yes	In-text, References
